# Supplementary material for: Development of a questionnaire to evaluate patients’ awareness of cardiovascular disease risk in England’s National Health Service Health Check preventive cardiovascular programme
Source: BMJ Open. 2017 Sep 25;7(9):e014413. doi: 10.1136/bmjopen-2016-014413 (PMC5623403; doi:10.1136/bmjopen-2016-014413)
Supplement: Supplementary data [file bmjopen-2016-014413supp001.pdf]

Appendix A. 85 Item Questionnaire

Subscale	Items	Answers
Knowledge of CVD Risk and Prevention	1. Eating a lot of red meat increases heart attack and stroke risk.	True, False, Don't Know<br>T=True F=False<br>Correct Answers<br>Q1=T Q2=F Q3=T Q4=F Q5=T Q6=F Q7=F Q8=T Q9=T Q10=T Q11=F Q12=T Q13=T Q14=T Q15=T Q16=F Q17=T Q18=F
	2. Most people can tell whether or not they have high blood pressure.	
	3. You can reduce your risk of heart attack or stroke by being physically active.	
	4. 'High' blood pressure is defined as 110/80 (systolic/diastolic) or higher.	
	5. Dietary fibre lowers blood cholesterol.	
	6. The most important cause of heart attack and stroke is stress.	
	7. Trans-fats are healthier for the heart than most other kinds of fats.	
	8. Walking and gardening are considered types of exercise that can lower the risk of having a heart attack or stroke.	
	9. You can reduce your chance of developing a heart attack or stroke by eating five-a-day diet of fruits and vegetables.	
	10. Moderate physical activity of 150 minutes a week will reduce your chances of developing a heart attack or stroke.	
	11. People who quit smoking by 60 add five years to their life.	
	12. People who have diabetes are at higher risk having a heart attack or stroke.	
	13. Managing your stress levels will help you to manage your blood pressure.	
	14. HDL refers to 'good' cholesterol, and LDL refers to 'bad' cholesterol.	
	15. The healthiest exercise for the heart involves rapid breathing for a sustained period of time.	
	16. Many vegetables are high in cholesterol.	
	17. You are more likely to have a heart attack or stroke if you're overweight or obese.	
	18. Drinking alcohol has nothing to do with reducing the risk of heart attack or stroke.	
Perceived Risk and Vulnerability of CVD	19. There is a possibility that I will have a heart attack or stroke.	1 = Strongly disagree; 2 = disagree; 3 = agree; 4 = strongly agree
	20. There is a good chance I will experience a heart attack or stroke during the next 10 years.	
	21. A person who gets a heart attack or stroke has no chance of recovering.	
	22. I have a high chance of getting a heart attack or stroke because of my past behaviours.	
	23. I feel sure that I will have a heart attack or stroke.	
	24. Healthy lifestyle habits are unattainable.	
	25. It is likely that I will get a heart attack or stroke.	
	26. I am at risk for having a heart attack or stroke.	
	27. It is possible that I will have a heart attack or stroke.	
	28. I am not doing anything now that is unhealthy to my heart.	
	29. I am too young to have a heart attack or stroke.	
	30. People like me do not get a heart attack or stroke.	
	31. I am very healthy so I will not have a heart attack or stroke.	
	32. I am not worried that I might have a heart attack or stroke.	
	33. People my age are too young to have a heart attack or stroke.	

	34. People my age do not have a heart attack or stroke.	
	35. My lifestyle habits do not put me at risk for having a heart attack or stroke.	
	36. No matter what I do, if I am going to have a heart attack or stroke, I will have one.	
	37. People who do not have a heart attack or stroke are just plain lucky.	
	38. The causes of a heart attack or stroke are unknown.	
Perceived Susceptibility	39. It is likely that I will suffer from a heart attack or stroke in the future.	
	40. My chances of suffering from a heart attack or stroke in the next few years are great.	
	41. Having a heart attack or stroke is currently a possibility for me.	
	42. I feel I will suffer from a heart attack or stroke sometime during my life.	
	43. I am concerned about the likelihood of having a heart attack or stroke in the near future.	
Perceived Severity	44. Heart attacks and strokes are always fatal.	
	45. Having a heart attack or stroke will threaten my relationship with my significant other.	
	46. My whole life would change if I had a heart attack or stroke.	
	47. Having a heart attack or stroke would have a very bad effect on my sex life.	
	48. If I have a heart attack or stroke I will die within 10 years.	
Perceived Benefits	49. Increasing my exercise will decrease my chances of having a heart attack or stroke.	
	50. Eating a healthy diet will decrease my chance of having a heart attack or stroke.	
	51. Stopping smoking will reduce my chance of having a heart attack or stroke.	
	52. When I exercise I am doing something good for myself.	
	53. When I eat healthy I am doing something good for myself.	
	54. Cutting down on alcohol will decrease my chances of having a heart attack or stroke.	
Perceived Barriers	55. I do not know appropriate exercises to perform to reduce my risk of developing cardiovascular disease.	
	56. I do not know the recommended drinking limits for men or women.	
	57. I do not have time to exercise for 30 minutes a day on most days of the week.	
	58. I do not know what is considered a healthy diet that would prevent me from developing cardiovascular disease.	
	59. I will not have energy if I stop smoking.	
	60. I cannot afford to buy healthy foods.	
	61. I have other problems more important than worrying about diet and exercise.	
Self Efficacy	62. How confident are you that you know or can control the risks of having a heart attack or stroke?	1= not at all confident, 2=somewhat confident, 3= moderately confident, 4=very confident,
	63. How confident are you that you know or can maintain a healthy weight by exercising regularly?	
	64. How confident are you that you know or can stop smoking if you want to?	
	65. How confident are you that you know or can consume less alcohol?	

	66. How confident are you that you know or can control your blood pressure and/or cholesterol levels by taking your prescribed medications?	5=completely confident
	67. How confident are you that you know or can eat a healthy and balanced diet?	
Intention to Change Behaviour or Cues to Action	68. I want to stop smoking (if you do smoke).	1 = Strongly disagree; 2 = disagree; 3 = agree; 4 = strongly agree
	69. I intend to maintain a healthy weight.	
	70. I intend to be physically active within two months.	
	71. I expect to maintain a healthy weight.	
	72. I want to be physically active.	
	73. I intend to eat a healthy and balanced diet within two months.	
	74. I expect to stop smoking (if you do smoke).	
	75. I want to cut down on alcohol.	
	76. I want to maintain a healthy and balanced diet.	
	77. I intend to stop smoking within two months (if you do smoke).	
	78. I expect to eat a healthy and balanced diet.	
	79. I intend to cut down on alcohol in the next two months.	
	80. I expect to be physically active.	
	81. I expect to cut down on alcohol.	
	82. I want to eat a healthy and balanced diet.	
	83. I expect to take my medication to control my blood pressure and/or cholesterol	
	84. I want to take my medication to control my blood pressure and/or cholesterol	
	85. I intend to take my medication to control my blood pressure and/or cholesterol within two months	

Appendix B. 65 Item Questionnaire

Scale	Subscale	Items	Coding of Answers
**Knowledge**<br><br>(15 items)<br><br>Higher sum score = more knowledgeable / more correct	**CVD Risk**<br><br>**Knowledge –**<br><br>**Risk of having**<br><br>**a heart attack**<br><br>**/ stroke**<br><br>(15 items)	1. Eating a lot of red meat increases heart attack and stroke risk.	Correct Answers<br><br>Q1-T Q6-T Q11-F<br>Q2-F Q7-T Q12-T<br>Q3-T Q8-T Q13-T<br>Q4-T Q9-T Q14-T<br>Q5-T Q10-T Q15-F<br><br>T= True<br>F= False<br><br>Correct: Score = 1, Incorrect or Don't Know Score = 0.
		2. Most people can tell whether or not they have high blood pressure.	
		3. You can reduce your risk of heart attack or stroke by being physically active.	
		4. One of the main causes of heart attack and stroke is stress.	
		5. Walking and gardening are considered types of exercise that can lower the risk of having a heart attack or stroke.	
		6. You can reduce your chance of developing a heart attack or stroke by eating at least five portions of fruit and vegetables a day.	
		7. Moderate intensity activity of 2 ½ hours a week will reduce your chances of developing a heart attack or stroke.	
		8. People who have diabetes are at higher risk of having a heart attack or stroke.	
		9. Managing your stress levels will help you to manage your blood pressure.	
		10. The healthiest exercise for the heart involves rapid breathing for 10 minutes or more.	
		11. Many vegetables are high in cholesterol.	
		12. You are more likely to have a heart attack or stroke if you're overweight or obese.	
		13. Drinking high levels of alcohol can increase your cholesterol and triglyceride levels.	
		14. HDL refers to 'good' cholesterol, and LDL refers to 'bad' cholesterol.	
		15. Family history of heart disease is not a risk	

		factor for high blood pressure.	
**Perceived CVD Risk** (15 items) Composite score = sum across subscales. Higher score = higher perception of risk of having a heart attack or stroke	**Dread Risk** (7 items)	16. There is a possibility that I will have a heart attack or stroke.	Higher sum score = Higher perceived lack of control, dread, catastrophic potential and fatal consequences<br><br>1 = Strongly disagree; 2 = disagree; 3 = agree; 4 = strongly agree; N/A = 0
		17. There is a good chance I will experience a heart attack or stroke in the next 10 years.	
		18. It is likely I will have a heart attack or stroke because of my past and/or present behaviours.	
		19. I feel sure that I will have a heart attack or stroke.	
		20. It is likely that I will have a heart attack or stroke some time during my life.	
		21. I am at risk for having a heart attack or stroke some time during my life.	
		22. It is possible that I will have a heart attack or stroke within the next 10 years.	
	**Risk** (3 items)	23. I am too young to have a heart attack or stroke.	Higher sum score = Higher perceived hazards that has few, moderate, known outcomes and consequences<br><br>**Reverse coded**<br>4 = Strongly disagree; 3 = disagree; 2 = agree; 1 = strongly agree; N/A = 0
		24. People like me do not have a heart attack or stroke.	
		25. I am not worried that I might have a heart attack or stroke.	
	**Unknown Risk** (5 items)	26. I am not doing anything now that is unhealthy to my heart.	Higher sum score = Higher perceived hazards judged to be unobservable, unknown, new, and delayed in their manifestation of harm<br><br>**Reverse coded**<br>4 = Strongly disagree; 3 = disagree; 2 = agree; 1 = strongly agree; N/A = 0
		27. I am very healthy so I will not have a heart attack or stroke.	
		28. My lifestyle habits do not put me at risk for having a heart attack or stroke.	
		29. No matter what I do, if I am going to have a heart attack or stroke, I will have one.	
		30. People who do not have a heart attack or stroke are just plain lucky.	

**CVD Health Beliefs** (13 items)	**Susceptibility** (4 items)	31. It is likely that I will suffer from a heart attack or stroke in the future.	Higher average score = Higher perceived personal risk of heart attack and stroke<br><br>1 = Strongly disagree; 2 = disagree; 3 = agree; 4 = strongly agree; N/A = 0
		32. My chances of suffering from a heart attack or stroke in the next 10 years are great.	
		33. I feel I will suffer from a heart attack or stroke sometime during my life.	
		34. I am concerned about the likelihood of having a heart attack or stroke in the near future.	
	**Severity** (3 items)	35. Heart attacks and strokes are always fatal.	Higher average score = Higher perceived severity of heart attack and stroke<br><br>1 = Strongly disagree; 2 = disagree; 3 = agree; 4 = strongly agree; N/A = 0
		36. My whole life would change if I had a heart attack or stroke.	
		37. If I have a heart attack or stroke I will die within 10 years.	
	**Benefits** (4 items)	38. Eating at least five portions of fruit and vegetables a day will decrease my chances of having a heart attack or stroke.	Higher average score = Higher perceived benefits of diet, exercise, consuming less alcohol and smoking cessation for reducing risk for heart attack and stroke<br><br>**Reverse coded**<br>4 = Strongly disagree; 3 = disagree; 2 = agree; 1 = strongly agree; N/A = 0
		39. Increasing my exercise to at least 2 ½ hours a week will decrease my chances of having a heart attack or stroke.	
		40. When I exercise for at least 2 ½ hours a week I am doing something good for the health of my heart.	
		41. When I eat at least five portions of fruit and vegetables a day I am doing something good for the health of my heart.	
	**Barriers** (2 items)	42. I do not have time to exercise on most days of the week.	Higher average score = Higher perception of select barriers to engaging in heart attack and stroke risk reducing behaviours<br><br>1 = Strongly disagree; 2 = disagree; 3 = agree; 4 = strongly agree; N/A = 0
		43. I cannot afford to buy healthy foods.	
**Self**	**CVD risk**	How confident are you that you know how to or can…	Higher average score = higher perceived

**Efficacy**<br><br>(5 items)	**reduction**<br><br>**self efficacy**<br><br>(5 items)	44. Control the risks of having a heart attack or stroke.	confidence<br><br>1 = Not at all confident; 2 = somewhat confident; 3 = very confident; 4 = completely confident; N/A = 0
		45. Maintain a healthy weight by exercising at least 2½ hours a week within the next two months.	
		46. Stop smoking if you want to.	
		47. Drink within the recommended levels of alcohol.	
		48. Eat at least five portions of fruit and vegetables per day within the next two months.	
**Intention /**<br><br>**Readiness**<br><br>**to Change**<br><br>(17 items)	**Exercise**<br><br>(4 items)	49. I am not thinking about exercising for 2 ½ hours a week.	Higher average score = Higher perceived readiness for change with regard to exercise behaviour<br><br>1 = Strongly disagree; 2 = disagree; 3 = agree; 4 = strongly agree; N/A = 0
		50. I am thinking about exercising at least 2 ½ hours a week.	
		51. I intend or want to exercise at least 2 ½ hours a week.	
		52. I am ready or have started to exercise 2 ½ hours a week.	
	**Diet**<br><br>(4 items)	53. I am not thinking about eating at least five portions of fruit and vegetables a day.	Higher average score = Higher perceived readiness for change with regard to health dietary behaviour<br><br>1 = Strongly disagree; 2 = disagree; 3 = agree; 4 = strongly agree; N/A = 0
		54. I am thinking about eating at least five portions of fruit and vegetables a day.	
		55. I intend or want to eat at least five portions of fruit and vegetables a day.	
		56. I am ready or started to eat at least five portions of fruit and vegetables a day.	
	**Alcohol**<br><br>(4 items)	57. I am thinking about cutting down on alcohol.	Higher average score = Higher perceived readiness for change with regard to alcohol consumption behaviour<br><br>1 = Strongly disagree; 2 = disagree; 3 = agree; 4 = strongly agree; N/A = 0
		58. I intend or want to cut down on alcohol.	
		59. I have been cutting down on alcohol.	
		60. I am not thinking about cutting down on alcohol.	
	**Smoking**<br><br>(5 items)	61. I am thinking of stopping smoking within two months.	Higher average score = Higher perceived readiness for change with regard to smoking cessation

		62. I have reduced or stopped smoking.	behaviour<br><br>1 = Strongly disagree; 2 = disagree; 3 = agree; 4 = strongly agree; N/A = 0
		63. I intend or want to stop smoking.	
		64. If I stop smoking it will reduce my chances of having a heart attack or stroke.	
		65. I am not thinking about stopping smoking.	

Appendix C. Population characteristics of 110 NHS Health Check attendees

Population Characteristics		n	% Total
Gender	Male	51	46.4%
	Female	56	50.9%
Age group	40-49	45	40.9%
	50-59	40	36.4%
	60-74	14	12.7%
Ethnicity	White	93	84.5%
	Mixed	2	1.8%
	Asian	2	1.8%
	Black	4	3.6%
	Other	4	3.6%
*Deprivation**	IMD 1 - least deprived	14	12.7%
	IMD 2	30	27.3%
	IMD 3	12	10.9%
	IMD 4	31	28.2%
	IMD 5 - most deprived	14	12.7%
Cholesterol	Raised total cholesterol TC≥ 5 mmol/l	66	60.0%
Blood pressure	High blood pressure BP ≥ 140/90 mm Hg	28	25.5%
Body Mass Index (BMI)	Obese (BMI>=30)	26	23.6%
Physical activity	Physically inactive	22	20.0%
Smoking status	Smokers	20	18.2%
Alcohol consumption	Excessive drinkers	13	11.8%
*10 year predicted risk of CVD***	High CVD Risk (QRisk2>=20%)	5	4.5%
	Medium CVD Risk (10%=<Qrisk2<20%)	21	19.1%
	Low CVD Risk (QRisk2<10%)	85	77.3%
Mean Values & 95% Confidence Intervals	Age (95% CI)	51.52	(49.93, 53.12)
	Total Cholesterol (95% CI)	5.42	(5.19, 5.64)
	HDL Cholesterol (95% CI)	1.44	(1.36, 1.53)
	Cholesterol Ratio (TC/HDL) (95% CI)	4.12	(3.73, 4.52)
	SBP (95% CI)	129.60	(125.76, 133.44)
	DBP (95% CI)	81.63	(79.62, 83.63)
	BMI (95% CI)	27.24	(26.17, 28.31)
	Q-Risk 2 (95% CI)	6.27	(5.19, 7.34)

***Deprivation was measured using the Index of Multiple Deprivation (IMD).

**Ten year predicted risk of CVD was estimated using the Q-Risk 2 algorithm[1]

Notes: SBP = systolic blood pressure; DBP = diastolic blood pressure; CI = confidence interval; HDL = high density lipoprotein; CVD = cardiovascular disease

References

1. Hippisley-Cox J, Coupland C, Vinogradova Y, et al. Predicting cardiovascular risk in England and Wales: prospective derivation and validation of QRISK2. *BMJ*. 2008; 336.

Appendix D. The ABCD Risk Questionnaire and scoring guide

Scale	Items	Coding
**Knowledge**<br><br>Higher sum score = more knowledgeable / more correct about having a heart attack or stroke	1. One of the main causes of heart attack and stroke is stress.<br><br>2. Walking and gardening are considered types of exercise that can lower the risk of having a heart attack or stroke.<br><br>3. Moderately intense activity of 2 ½ hours a week will reduce your chances of having a heart attack or stroke.<br><br>4. People who have diabetes are at higher risk of having a heart attack or stroke.<br><br>5. Managing your stress levels will help you to manage your blood pressure.<br><br>6. Drinking high levels of alcohol can increase your cholesterol and triglyceride levels.<br><br>7. HDL refers to 'good' cholesterol, and LDL refers to 'bad' cholesterol.<br><br>8. A family history of heart disease is not a risk factor for high blood pressure.	Correct Answers:<br><br>Q1-T<br><br>Q2-T<br><br>Q3-T<br><br>Q4-T<br><br>Q5-T<br><br>Q6-T<br><br>Q7-T<br><br>Q8-F<br><br>T= True<br>F= False<br><br>Correct: Score = 1,<br>Incorrect or Don't Know: Score = 0.
**Perceived Risk of Heart**	9. I feel I will suffer from a heart attack or stroke sometime during my life.	1=Strongly disagree; 2 = disagree; 3 = agree; 4 = strongly agree; N/A = 0
**Attack/Stroke**	10. It is likely that I will suffer from a heart attack or stroke in the future.	1=Strongly disagree; 2 = disagree; 3 = agree; 4 = strongly agree; N/A = 0
Higher sum score =	11. It is likely that I will have a heart attack or stroke some time during my life.	1=Strongly disagree; 2 = disagree; 3 = agree; 4 = strongly agree; N/A = 0
higher perception of risk of having a heart attack or	12. There is a good chance I will experience a heart attack or stroke in the next 10 years.	1=Strongly disagree; 2 = disagree; 3 = agree; 4 = strongly agree; N/A = 0
stroke	13. My chances of suffering from a heart attack or stroke in the next 10 years are great.	1=Strongly disagree; 2 = disagree; 3 = agree; 4 = strongly agree; N/A = 0
**Perceived Risk of**	14. It is likely I will have a heart attack or stroke because of my past and/or present behaviours.	1=Strongly disagree; 2 = disagree; 3 = agree; 4 = strongly agree; N/A = 0

Scale	Items	Coding
**Heart Attack/Stroke**	15. I am not worried that I might have a heart attack or stroke.	**Reverse coded** 4=Strongly disagree; 3 = disagree; 2 = agree; 1 = strongly agree; N/A = 0
	16. I am concerned about the likelihood of having a heart attack or stroke in the near future.	1=Strongly disagree; 2 = disagree; 3 = agree; 4 = strongly agree; N/A = 0
**Perceived Benefits and Intentions to Change** Higher average score = Higher perceived benefits of diet and exercise and higher perceived readiness for change in regards to exercise behaviour	17. I am thinking about exercising at least 2½ hours a week.	1=Strongly disagree; 2 = disagree; 3 = agree; 4 = strongly agree; N/A = 0
	18. I intend or want to exercise at least 2½ hours a week.	1=Strongly disagree; 2 = disagree; 3 = agree; 4 = strongly agree; N/A = 0
	19. When I exercise for at least 2½ hours a week I am doing something good for the health of my heart.	1=Strongly disagree; 2 = disagree; 3 = agree; 4 = strongly agree; N/A = 0
	20. I am confident that I can maintain a healthy weight by exercising at least 2½ hours a week within the next two months.	1=Strongly disagree; 2 = disagree; 3 = agree; 4 = strongly agree; N/A = 0
	21. I am not thinking about exercising for 2 ½ hours a week.	**Reverse coded** 4=Strongly disagree; 3 = disagree; 2 = agree; 1 = strongly agree; N/A = 0
	22. When I eat at least five portions of fruit and vegetables a day I am doing something good for the health of my heart.	1=Strongly disagree; 2 = disagree; 3 = agree; 4 = strongly agree; N/A = 0
	23. Increasing my exercise to at least 2½ hours a week will decrease my chances of having a heart attack or stroke.	1=Strongly disagree; 2 = disagree; 3 = agree; 4 = strongly agree; N/A = 0
**Healthy Eating Intentions** Higher average score = Higher perceived readiness for change with regard to health dietary behaviour	24. I am confident that I can eat at least five portions of fruit and vegetables per day within the next two months.	1=Strongly disagree; 2 = disagree; 3 = agree; 4 = strongly agree; N/A = 0
	25. I am thinking about eating at least five portions of fruit and vegetables a day.	1=Strongly disagree; 2 = disagree; 3 = agree; 4 = strongly agree; N/A = 0
	26. I am not thinking about eating at least five portions of fruit and vegetables a day.	**Reverse coded** 4=Strongly disagree; 3 = disagree; 2 = agree; 1 = strongly agree; N/A = 0

The Attitudes and Beliefs about Cardiovascular Disease (ABCD) Risk Questionnaire is licensed under the Creative Commons Attribution 4.0 International License.

Appendix E. Correlations of Factors of the ABCD Risk Questionnaire with Predicted CVD Risk using Spearman's Rho among 110 NHS Health Check Attendees

		KNOWLEDGE	PERCEIVED RISK	PERCEIVED BENEFITS	HEALTHY INTENTIONS	IMD2010_Quintile	BMI	QRISK2	QRISK2_L_M_H
KNOWLEDGE	Correlation Coefficient	1.000	-.124	-.148	-.106	-.002	-.225*	-.007	-.063
	Sig. (2-tailed)	.	.236	.175	.319	.986	.021	.941	.522
	N	107	93	86	91	99	105	104	104
PERCEIVED RISK	Correlation Coefficient	-.124	1.000	-.195	-.188	.239*	.389**	.220*	.173
	Sig. (2-tailed)	.236	.	.080	.088	.025	.000	.036	.102
	N	93	95	82	84	87	92	91	91
PERCEIVED BENEFITS	Correlation Coefficient	-.148	-.195	1.000	.533**	-.287**	-.068	-.118	-.232*
	Sig. (2-tailed)	.175	.080	.	.000	.009	.538	.284	.033
	N	86	82	88	83	81	85	84	84
HEALTHY INTENTIONS	Correlation Coefficient	-.106	-.188	.533**	1.000	-.261*	.084	-.072	-.116
	Sig. (2-tailed)	.319	.088	.000	.	.016	.430	.504	.279
	N	91	84	83	93	85	90	89	89

		KNOWLEDGE	PERCEIVED RISK	PERCEIVED BENEFITS	HEALTHY INTENTIONS	IMD2010_Quintile	BMI	QRISK2	QRISK2_L_M_H
IMD2010_Quintile	Correlation Coefficient	-.002	.239*	-.287**	-.261*	1.000	-.008	.009	.017
	Sig. (2-tailed)	.986	.025	.009	.016	.	.938	.931	.870
	N	99	87	81	85	101	101	100	100
BMI	Correlation Coefficient	-.225*	.389**	-.068	.084	-.008	1.000	.020	.028
	Sig. (2-tailed)	.021	.000	.538	.430	.938	.	.839	.777
	N	105	92	85	90	101	107	106	106
QRISK2	Correlation Coefficient	-.007	.220*	-.118	-.072	.009	.020	1.000	.694**
	Sig. (2-tailed)	.941	.036	.284	.504	.931	.839	.	.000
	N	104	91	84	89	100	106	106	106
QRISK2_L_M_H	Correlation Coefficient	-.063	.173	-.232*	-.116	.017	.028	.694**	1.000
	Sig. (2-tailed)	.522	.102	.033	.279	.870	.777	.000	.
	N	104	91	84	89	100	106	106	106

* Correlation is significant at the 0.05 level (2-tailed). ** Correlation is significant at the 0.01 level (2-tailed).

QRisk2_L_M_H categorizes predicted CVD risk from Low CVD Risk (QRISK2<10%), to Medium CVD Risk (10%<=QRISK2<20%), to High CVD Risk (QRISK2>=20%).

IMD2010Quintile categorizes deprivation from 1=least deprived to 5=most deprived.
