# Supplementary material for: Development of a questionnaire to evaluate patients’ awareness of cardiovascular disease risk in England’s National Health Service Health Check preventive cardiovascular programme
Source: BMJ Open. 2017 Sep 25;7(9):e014413. doi: 10.1136/bmjopen-2016-014413 (PMC5623403; doi:10.1136/bmjopen-2016-014413)
Supplement: Author's manuscript [file bmjopen-2016-014413.draft_revisions.pdf]

BMJ Open is committed to open peer review. As part of this commitment we make the peer review history of every article we publish publicly available.

When an article is published we post the peer reviewers' comments and the authors' responses online. We also post the versions of the paper that were used during peer review. These are the versions that the peer review comments apply to.

The versions of the paper that follow are the versions that were submitted during the peer review process. They are not the versions of record or the final published versions. They should not be cited or distributed as the published version of this manuscript.

BMJ Open is an open access journal and the full, final, typeset and author-corrected version of record of the manuscript is available on our site with no access controls, subscription charges or pay-per-view fees (http://bmjopen.bmj.com).

If you have any questions on BMJ Open's open peer review process please email editorial.bmjopen@bmj.com

BMJ Open

Development of a tool to evaluate the effectiveness of National Health Service Health Check cardiovascular disease risk assessment: a questionnaire examining patients' awareness of risk

Journal:	*BMJ Open*
Manuscript ID	bmjopen-2016-014413
Article Type:	Research
Date Submitted by the Author:	22-Sep-2016

Complete List of Authors:	Woringer, Maria; Imperial College London Department of Primary Care and Public Health, 3rd Floor Reynolds Building Charing Cross Campus St Dunstans Road, W6 8RP<br>Nielsen, Jessica; City University London, School of Arts and Social Sciences, Department of Psychology, City University London Northampton Square, EC1V 0HB<br>Zibarras, Lara; City University London, School of Arts and Social Sciences, Department of Psychology, City University London Northampton Square, EC1V 0HB<br>Evason, Julie; Health Diagnostics Ltd, Chatham House, Dee Hills Park, CH3 5AR<br>Kassianos, Angelos; University College London, Department of Applied Health Research<br>Harris, Matthew; Imperial College London, Institute of Global Health Innovation, Level 1, Faculty Building South Kensington Campus, SW7 2NA ; Imperial College London Department of Primary Care and Public Health, 3rd Floor Reynolds Building Charing Cross Campus St Dunstans Road, W6 8RP<br>Majeed, Azeem; Imperial College London, Primary Care & Public Health, 3rd Floor Reynolds Building Charing Cross Campus St Dunstans Road, W6 8RP<br>Soljak, Michael; Imperial College London, Primary Care & Public Health, 3rd Floor Reynolds Building Charing Cross Campus St Dunstans Road, W6 8RP

**Primary Subject Heading**:	Communication
Secondary Subject Heading:	General practice / Family practice, Health services research
Keywords:	QUALITATIVE RESEARCH, PREVENTIVE MEDICINE, PRIMARY CARE, PUBLIC HEALTH

SCHOLARONE™ Manuscripts

For peer review only

Development of a tool to evaluate the effectiveness of National Health Service Health Check cardiovascular disease risk assessment: a questionnaire examining patients' awareness of risk

Author Names and Affiliations:

Ms. Maria Woringer (a)

Dr. Jessica Jones Nielsen (b)

Dr. Lara Zibarras (b)

Ms. Julie Evason (c)

Dr. Angelos P. Kassianos (d)

Dr. Matthew Harris (e)

Professor Azeem Majeed (a)

Dr. Michael Soljak (a)

Affiliations :

a) Department of Primary Care and Public Health, Imperial College London

b) Department of Psychology, City University London

c) Health Diagnostics Ltd, Chester, UK

d) Department of Applied Health Research, University College London

e) Institute of Global Health Innovation, Imperial College London

Word Count (excluding title page, declarations, abstract, keywords, strengths and limitations and references): 3,990

Author responsible for correspondences concerning the manuscript:

Maria Woringer; Imperial College London, Department of Primary Care and Public Health, Charing Cross Campus, The Reynolds Building, St Dunstan's Road, London W6 8RP, UK

m.woringer@imperial.ac.uk; Tel: +44(0)2075940789

For peer review only - http://bmjopen.bmj.com/site/about/guidelines.xhtml

For peer review only

List of Declarations:

Dissemination of Study Findings

The work on the development and validation of the patient questionnaire was presented as a poster titled "Development and Validation of the Attitudes and Beliefs about Cardiovascular Disease (ABCD) Risk Survey" at the NHS Health Check 2015 – Improvement through Collaboration conference in Leeds, England on 26 February, 2015. In addition, an abstract titled "Development and validation of a patient survey to assess the effectiveness of cardiovascular disease screening" was selected for oral presentation at the First International Conference of Public Health, Primary Care and Congress of Person Centred Medicine on October 29, 2015 and accepted for publication in the International Journal of Person Centred Medicine. This submission is not under consideration by any other journal. All authors have approved the manuscript and this submission.

Permissions

Favourable ethical opinion for the study - "Patient Evaluation of the NHS Health Check programme to Investigate the Programme's Effectiveness in Communicating CVD Risk" was obtained from the NRES Committee London – City & East reference number 13/LO/1885.

Study participants gave their written informed consent to participate in the study and to share their results and medical data.

The Attitudes and Beliefs about Cardiovascular Disease (ABCD) Risk Questionnaire is licensed under the Creative Commons Attribution-NonCommercial-NoDerivatives 4.0 International License.

Funding

This work was supported by the National Institute for Health Research (NIHR) Diagnostics Evidence Co-operatives (DEC) / Collaboration for Leadership in Applied Health Research and Care (CLAHRC) grant.

Data Sharing Agreement

No additional data available.

Competing Interests

None.

Contributors

MW, AM, MS, and HW designed the study, JE supplied the data. JJN designed the validation instrument, LZ performed the psychometric analysis. JE, AK, MH and AM reviewed the validation instrument's face and content validity. All authors discussed data analyses and interpreted the results. MW wrote the first draft of the manuscript. All authors critically revised and approved the final manuscript. MW had full access to all the data used in the study and takes responsibility for the integrity of the data and the accuracy of the data analysis. MW is the guarantor.

For peer review only - http://bmjopen.bmj.com/site/about/guidelines.xhtml

ABSTRACT

Background

The National Health Service (NHS) Health Check is a CVD risk assessment and management programme in England aiming to increase CVD risk awareness among people at increased risk of CVD. There is no tool to assess the effectiveness of the programme in communicating CVD risk to patients.

Aims

The aim of this paper was to develop a questionnaire examining patients' CVD risk awareness for use in health service research evaluations of the NHS Health Check programme.

Methods

We developed an 85 item questionnaire to determine patients' views of their risk of CVD. The questionnaire was based on a review of the relevant literature. After review by an expert panel and focus group discussion, 22 items were dropped and 2 new items were added. The resulting 65 item questionnaire with satisfactory content validity (content validity indices >=0.80) and face validity was tested on 110 NHS Health Check attendees in primary care in a cross sectional study between May and July 2014.

Results

Following analyses of data, we reduced the questionnaire from 65 to 26 items. The 26 item questionnaire constitutes 4 scales: Knowledge of CVD Risk and Prevention, Perceived Risk of Heart Attack/Stroke, Perceived Benefits and Intention to Change Behaviour and Healthy Eating Intentions. Perceived Risk (Cronbach's α = 0.85) and Perceived Benefits and Intention to Change Behaviour (Cronbach's α = 0.82) have satisfactory reliability (Cronbach's α >=0.70). Healthy Eating Intentions (Cronbach's α = 0.56) is below minimum threshold for reliability but acceptable for a three item scale.

Conclusions

The final questionnaire, with satisfactory reliability and validity, is recommended for use in evaluating the effectiveness of the Health Check programme in communicating CVD risk to patients.

Word Count: 276

Keywords: cardiovascular disease, primary prevention, risk assessment, questionnaire

Strengths and limitations of this study

- ABCD Risk Questionnaire – short, validated questionnaire measuring CVD risk awareness
- Questionnaire guided by literature review, expert panel, patient focus group and analysis of data
- Easy to understand questions for both patients and clinicians
- Patient focus group consisting of 6 individuals not representative of the target population
- Questionnaire tested on 110 individuals representative of the target population

For peer review only - http://bmjopen.bmj.com/site/about/guidelines.xhtml

- Additional studies with larger samples needed to confirm questionnaire reliability and validity

INTRODUCTION

Cardiovascular disease (CVD) is a major cause of disability and premature mortality worldwide accounting for a third of deaths annually in England.[1, 2] Ninety per cent of CVD cases are associated with modifiable lifestyle factors.[3, 4] Despite substantial reductions in mortality, modifiable risk factors contributed to only 34% of the overall decline in CVD mortality in England between 2000-2007.[5] Further gains could be made by promoting healthier lifestyle changes. CVD contributes considerably to the rising cost of healthcare and is estimated to cost the NHS and UK economy £30 billion annually. [1] In 2010 / 2011 there were 1.4 million hospital admissions related to CVD of which 60% were for people younger than 75 years of age and more than half were as an emergency. Preventing long term illness and disability associated with CVD is important for improving health while reducing healthcare costs.[6]

The National Health Service (NHS) Health Check programme may be important for preventing the premature onset of disease while reducing healthcare costs associated with CVD by identifying individuals at increased risk of CVD, raising their awareness of CVD risk and helping them manage their risk. [7-10] The NHS Health Check programme is a CVD risk assessment and management programme in England launched by the Department of Health in April 2009.[7] The programme aims to prevent heart disease, stroke, diabetes and kidney disease whilst reducing health inequalities.[8] Eligibility criteria is being 40-74 years old and free of vascular disease diagnosis. All participants are offered general lifestyle advice. People at high risk of CVD are offered statins, lifestyle advice and behaviour change support in relation to physical activity, smoking cessation, safe alcohol consumption and healthy diet. The programme has a potential to prevent 1,600 heart attacks and strokes, 650 premature deaths, and over 4,000 new cases of diabetes each year.[9] Projected programme cost is £180-£243 million/year at full implementation with estimated cost per quality adjusted life year (QALY) being £3,000.[10]

In order to adopt healthy lifestyle behaviours related to diet, exercise, smoking and alcohol consumption, the general population must be aware of CVD risk.[11] In the context of the NHS Health Check Programme, CVD risk awareness refers to the accuracy of perceived risk of CVD against predicted CVD risk, general knowledge of CVD and what one can do to lower predicted CVD risk. Whereas predicted CVD risk refers to one's chance of experiencing a heart attack or stroke,[12] perceived risk of CVD refers to a person's perception of their CVD risk. While as many as 40% of the general population underestimate their CVD risk, 20% overestimate their risk.[13] False reassurance may lead to adoption and or maintenance of unhealthy behaviours contributing to the premature onset of CVD. Low CVD risk awareness is reported among men, inner city residents, and people of lower socioeconomic status. [11 14 15] Although the NHS Health Check programme was shown to have modest reductions in predicted 10 year risk of CVD,[16] it is not known whether or not the Health Check results in improved CVD risk awareness.

Since the aim of a CVD risk assessment and management programme is to identify and empower individuals (who are either at increased CVD risk or have one or more risk factors for CVD) to make important lifestyle choices or changes to prevent the premature onset of CVD, the effectiveness of such a programme cannot be ascertained without accurately and reliably assessing patient views.

For peer review only - http://bmjopen.bmj.com/site/about/guidelines.xhtml

Although a number of validated questionnaires were developed to measure knowledge, perceptions of CVD or intention to change behaviour,[15-17] no short, validated questionnaire was developed to assess CVD risk awareness using all of these scales. Until now studies examining the accuracy of perceived risk and knowledge of CVD relied on tools that were not validated.[17] The problem with using non-validated tools is that the questions may not accurately and reliably capture individuals' views or measure what they intend to measure. The aim of this work was to develop a questionnaire with satisfactory face, content validity and reliability to assess the effectiveness of the NHS Health Check programme in raising patients' awareness of CVD risk.

METHODS

The first phase of development of the questionnaire was guided by a literature review, an expert panel and a patient focus group. At each stage of questionnaire development, the number of items was reduced (see Figure 1).

Figure 1 Flowchart of Phase I of Questionnaire Development

The second phase of questionnaire development was guided by an analysis of data from 110 NHS Health Check attendees who completed the 65 item questionnaire. The number of questionnaire items was further reduced (see Figure 2).

Figure 2 Flowchart of Phase II of Questionnaire Development

Phase I of Questionnaire Development

Construction of draft questionnaire by review of relevant literature

We performed an extensive literature review pertaining to CVD risk awareness between December 2013 and January 2014 in the areas of disease knowledge, risk perception, intention to change and self-efficacy related to CVD and HBM to guide initial item development. PubMed and PsycINFO databases and Google Scholar Articles were utilised to search for existing instruments that measure perception of CVD risk, CVD knowledge and self-efficacy with no limits on the year of publication. The following key words were used to identify the relevant literature: "cardiovascular disease" "heart disease" " knowledge" "risk" "test" "questionnaire" "scale" "assessment" "self-efficacy" "perception" "health belief model". Questionnaires were considered if they addressed CVD risk awareness, reported moderate to high scores of reliability and validity in population studies and had suitable wording and level of understanding. Questionnaires were excluded if they pertained to individuals under the age of 15 as these people would not be eligible to receive an NHS Health Check, focused on risk unrelated to heart attack or stroke, and were not written in English.

Although a number of questionnaires were found measuring different aspects of CVD risk awareness such as heart disease knowledge, perception of CVD risk, perceived susceptibility and severity of CVD and benefits and barriers to adopting healthy behaviours,[18-20] no single questionnaire encompassed them all. Initial item development was guided by the Health Belief Model (HBM)[21] and the Transtheoretical Model (TTM).[22] According to HBM, individuals who have accurate knowledge of CVD and perceived susceptibility to and consequences of the disease, and are aware of the benefits of taking preventive measures are more likely to make important lifestyle choices to prevent the

For peer review only - http://bmjopen.bmj.com/site/about/guidelines.xhtml

onset of disease.[23] The TTM describes behavioural change as a staged process over time including pre-contemplation, contemplation, preparation, action and maintenance.[22] Sixty five items were selected using validated questionnaires addressing CVD knowledge, and the main constructs of HBM such as perceived susceptibility, perceived severity, perceived benefits of changing behaviours, and perceived barriers to making changes.[18-20] In addition 23 new items were generated to identify perceived levels of readiness to engage in CVD risk reduction behaviours (using TTM) and self-efficacy (confidence in ability to change health behaviour) in relation to exercise, diet, smoking cessation and decreasing alcohol consumption.[24, 25] These items were based on behaviour specific recommendations of the NHS Health Check programme such as stopping smoking, consuming no more than 14 units of alcohol a week, eating at least five portions of fruit and vegetables a day and exercising at least 150 minutes per week.[26-28] The resulting 85 item questionnaire is in Appendix A.

Modification of questionnaire by expert panel to obtain satisfactory content validity

A panel of experts in the areas of CVD, health psychology, public health, psychometrics and questionnaire development and medicine were asked to evaluate each item and the total 85 item questionnaire for content validity in February 2014. Experts assessed content validity of the questionnaire by examining whether the items were representative of the content they were intended to measure.[29] Items were examined for representatives of the scale domain, appropriateness and relevance. The content validity index (CVI), a widely used technique in scale development determined item and questionnaire clarity, homogeneity, and relevance on a 4-point Likert scale (ranging from 1 = *an irrelevant item* to 4 = *an extremely relevant item*).[30, 31] A CVI of ≥ 0.80 is recommended. [32, 33] Experts were asked the following questions: "*Do these items belong together in the subscale?*" and "*Does each item belong in the set?*" For ratings of content validity, experts were asked whether the subscale definition and label fitted the set of items presented; whether each item belonged with the label and definition; and whether each item was unique in its contribution to the subscale.

Modification of questionnaire by patient focus group to obtain satisfactory face validity

Researchers facilitated a patient focus group to assess the face validity of the 69 item questionnaire resulting from the expert review. Face validity is assessed by end users deciding whether the questionnaire appears to measure what the researchers who developed it claim.[33] A convenience sample of six individuals was recruited on March 4, 2014 from the County Durham and Darlington National Health Service Foundation Trust. Eligibility criteria was being aged 40-74 years and being free of known vascular disease. The focus group consisted of six white females between 50-64 years of age. The majority of participants had postgraduate education and worked in a health-related field. Participants were asked to complete the 69 item questionnaire as well as to provide feedback on whether the items correctly measured the intended scales, appropriately stated the intent of the questionnaire, and matched the individual's situations.[32, 33] In addition, participants were asked to respond to questions about clarity, content, appropriateness, format, biases of questions and presentation of information. The resulting 65 item questionnaire is in Appendix B.

Phase II of Questionnaire Development

Modification of questionnaire to have satisfactory reliability

For peer review only - http://bmjopen.bmj.com/site/about/guidelines.xhtml

A 65 item questionnaire was administered to 110 NHS Health Check attendees immediately after their consultation between May and July 2014 in a cross sectional study in England. The aim was to determine the content, the scale structure and the reliability of a questionnaire in its final form.

Study Population

Eligibility criteria was completion of an NHS Health Check, being aged 40-74 years and free of known vascular disease. Of 110 study participants, 15 individuals were recruited from a London general practice and 95 from local community venues in Durham. During the NHS Health Check, nurses collected clinical risk factor data, informed study participants about their CVD risk and administered the 65 item questionnaire.

Data Analysis

To select appropriate items to constitute a scale, individual items were assessed during item analysis, item facility and item discrimination.[34] To determine the factorial structure of the questionnaire and which items together constituted particular scales, an Exploratory Factor Analysis (EFA) - a widely used technique in scale development was performed.[30, 35] The reliability of factors constituting particular scales was assessed using Cronbach's alpha coefficient. [36, 37] Reliability refers to consistency, reproducibility and agreement of a scale.[38]

In order to improve the quality of a scale and increase its reliability, individual items were assessed. Items with reverse scoring were re-coded to conform to the conceptual direction of the scales.[37] Each individual item was then examined for distortions in the pattern of responding known as skew and kurtosis.[33] Item facility examined whether items were answered in the same way by everyone by checking whether the facility index approached extreme scores or had a low standard deviation.[34] Items were assessed in terms of discriminating between participants' responses to the questionnaire's scales (Knowledge, Perceived CVD Risk, CVD Health Beliefs, Intentions / Readiness to Change and Self Efficacy). Discrimination was measured by item-total correlation with item correlating below 0.2 or any negative correlations resulting in deletion of items. In addition, discrimination was measured by the inter-item correlation within each scale resulting in deletion of items correlating with other items ≥0.60.[18, 34]

A Kaiser-Meyer-Olkin (KMO) measure of sampling adequacy and a Bartlett's test of sphericity were assessed to ensure that items were appropriate for EFA.[39] Next EFA was performed to define the scales of the questionnaire which share a similar underlying construct. Parallel Analysis was used to determine the optimum number of factors to be extracted using Principal Components Analysis (PCA) with a Varimax rotation. [34, 39, 40] PCA is a data reduction technique used to explain correlations among sets of items or variables as a few conceptually meaningful factors.[41] Compared to other available methods, Parallel Analysis using PCA was shown to be the best method of extracting factors and is appropriate when applied to data conforming to the formal factor analytic model.[39, 40] Iterations of EFA were carried out to identify core constituent items in each factor. Cross-loading items or items with loading <=0.50 were removed at each iteration.[39] Internal consistency reliability of resulting factors was assessed using Cronbach's α coefficients with α>=0.70 indicating good reliability.[32,36, 37]

RESULTS

For peer review only - http://bmjopen.bmj.com/site/about/guidelines.xhtml

Construction of a draft questionnaire by review of relevant literature

We developed an 85 item questionnaire based on the theoretical framework, NHS guidelines and other validated questionnaires relating to heart disease.[18-20] The 85 item questionnaire had 8 subscales measuring *Knowledge of CVD Risk and Prevention* (18 items), *Perceived Risk and Vulnerability of CVD* (20 items), *Perceived Susceptibility* (5 items), *Perceived Severity* (5 items), *Perceived Benefits* (6 items), *Perceived Barriers* (7 items), *Self-Efficacy* (6 items), and *Intention to Change Behaviour* (18 items). *Knowledge of CVD Risk and Prevention* subscale items were measured using the following categories: *True, False*, and *Don't Know*. *Self-Efficacy* subscale items were measured using 5 point Likert scale ranging from *1=not at all confident* to *5=completely confident*. *Perceived Severity, Perceived Benefits, Perceived Barriers and Intention to Change Behaviour* subscale items were measured using a 4 point Likert scale ranging from *1=strongly disagree* to *4=strongly agree*. The reading level of the questionnaire was at Year 7.

Modification of questionnaire by expert panel to obtain satisfactory content validity

The expert panel concluded that out of the 85 items, 69 met the CVI>=0.80 criterion and were retained. In addition, the wording of a number of questions was revised to improve clarity. Diet and exercise were defined more precisely using frequency and duration. Response options of Self-Efficacy items were changed from a five point Likert scale to a four point Likert scale for consistency with the rest of the questionnaire. Questions pertaining to smoking and drinking were rephrased to apply to smokers and drinkers (see Table 1).

Table 1 Sample item wording modifications obtained through an expert panel

**Original item(s)**	**Expert comments**	**Final item**
The most important cause of heart attack and strike is stress.	Revise to "one of the most important…" Substitute the word "important" with "main."	One of the main causes of heart attack and stroke is stress.
I have a high chance of getting a heart attack or stroke because of my past behaviours.	Add "and/or present behaviours."	I have a high chance of getting a heart attack or stroke because of my past and/or present behaviours.
Increasing my exercise will decrease my chances of having a heart attack or stroke.	Define amount of exercise.	Increasing my exercise to at least 30 minutes a day will decrease my chances of having a heart attack or stroke.
Eating a healthy diet will	Define a healthy diet.	Eating at least five portions of fruit and vegetables a day will

For peer review only - http://bmjopen.bmj.com/site/about/guidelines.xhtml

Original item(s)	Expert comments	Final item
decrease my chance of having a heart attack or stroke.		decrease my chances of having a heart attack or stroke.
When I exercise I am doing something good for myself.	Define exercise consistently. Make the statement more specific about the heart.	When I exercise for 30 minutes a day I am doing something good for the health of my heart.
How confident are you that you know or can…? questions answered using a 5-point Likert scale: "not at all confident, somewhat confident, moderately confident, very confident, completely confident."	Use a 4-point Likert to maintain consistency.	Five point Likert scale changed to a 4 point Likert scale: "not at all confident, somewhat confident, very confident, completely confident."
How confident are you that you know how or can stop smoking if you want to?	Instead of saying "…that you know or can" say "that you know how to or can…" Add in parentheses "if you smoke."	How confident are you that you know how to or can stop smoking if you want to (if you smoke)?
I want to cut down on alcohol. I intend to cut down on alcohol in the next two months.	Conceptual overlap between want to and intend to. Add in parentheses "if you drink alcohol."	I intend or want to cut down on alcohol (if you drink alcohol).

Modification of questionnaire by patient focus group to obtain satisfactory face validity

As a result of the focus group review of the 69 item questionnaire, six items were removed, two items were added and a number of items were modified leaving a final total of 65 items with satisfactory face validity. A not applicable category was added to 50 items while the response categories to Knowledge subscale items remained unchanged. Exercise was redefined in 8 items from 150 minutes a week and 30 minutes a day to 2.5 hours a week. A negatively framed question was reframed positively (see Table 2).

Table 2 Sample item wording modifications and additions through the patient focus group

Original item	Participant comments	Final item
Moderate physical activity	2.5 hours a week is better than	Moderate physical activity of

For peer review only - http://bmjopen.bmj.com/site/about/guidelines.xhtml

Original item	Participant comments	Final item
of 150 minutes a week will reduce your chances of developing a heart or stroke.	150 minutes.	2.5 hours a week will reduce your chances of developing a heart or stroke.
Drinking alcohol has nothing to do with reducing the risk of heart attack or stroke.	Question is negatively stated.	Drinking high levels of alcohol can increase your cholesterol and triglyceride levels.
Missing question	Need to include family history of disease to account for genetic predisposition.	A family history of hypertension is not a risk factor for high blood pressure.
Missing question	Benefits of not smoking?	If I stopped smoking it will reduce my chances of having a heart attack or stroke.
Increasing my exercise for 30 minutes a day will decrease my chances of having a heart attack or stroke.	Two and a half hours a week is better than 30 minutes a day.	Increasing my exercise to at least 2 ½ hours a week will decrease my chances of having a heart attack or stroke.
I have reduced or stopped smoking (if you smoke). "strongly disagree, disagree, agree, and strongly agree."	Remove (if you smoke). Add a "not applicable" box.	I have reduced or stopped smoking. "strongly disagree, disagree, agree, and strongly agree, not applicable."
How confident are you that you know how to or can consume recommended levels of alcohol (if you drink alcohol)? "not at all confident, somewhat confident, very confident and completely confident."	Remove (if you drink alcohol). Add a "not applicable" box.	How confident are you that you know how to or can drink within the recommended levels of alcohol? "not at all confident, somewhat confident, very confident and completely confident, not applicable."

Modification of questionnaire to have satisfactory reliability

The 65 item questionnaire that resulted from content and face validity assessments, was administered to 110 NHS Health Check attendees immediately after their NHS Health Check

For peer review only - http://bmjopen.bmj.com/site/about/guidelines.xhtml

consultation. The majority of study participants were White (84.5%), younger than 60 (77.3%) and had at least one or more CVD risk factors. Using the Index of Multiple Deprivation, a relative measure of deprivation across seven distinct domains including income, employment, health and disability, education skills and training, barriers to housing and services, living environment and crime, [42] people in the two most deprived fifths were 40.0% of the study population. See Appendix C for study population characteristics. The responses to the questionnaire were analysed as individual items during item analysis, item facility and item discrimination. In addition, the scale structure and reliability of resulting scales were assessed.

No items were removed during item analysis and item facility. During item discrimination assessment using item-total correlation, seven items in the Knowledge scale, four items in Perceived CVD Risk, three items in CVD Health Benefits, three items in Intention and or Readiness to Change were deleted due to item-total correlations falling below 0.2. [33] During item discrimination assessment using inter-item correlation, two items in Perceived CVD Risk and three items in Intentions / Readiness to Change were removed as these items correlated greater than 0.6 with other items.[33] Although there were two items that correlated above 0.6 in CVD Risk Reduction Self Efficacy, these remained in the questionnaire as the items were qualitatively different: *Stop smoking if you want to* and *Control the risks of having a heart attack or stroke.* In total, 22 items were removed during item discrimination analysis, leaving 43 items which had good item facility and discrimination.

Of the 43 remaining items, 8 items of the "Knowledge" scale with *"true"* or *"false"* scoring could not entered into EFA. Of the 35 items scored on a four point Likert scale, four items pertaining to smoking were deleted as they had a high proportion of missing responses (69-80%). The resulting 31 items had a Kaiser-Meyer-Olkin (KMO) measure of sampling adequacy of 0.32 and a significant Bartlett's test of sphericity (1020.50, p <.001), indicating that these data were appropriate for EFA.[39] After 12 iterations of EFA, 20 items loaded above 0.50 on the factors and there were no cross-loadings indicating good factor structure (see Table 3). Internal consistency reliability of factor structure was measured using Cronbach's α. Factor 1 (8 items): (Perceived Risk of Heart Attack/Stroke) had α =.85. Factor 2 (7 items): (Perceived Benefits & Intentions to Change) had α =.82. Factor 3 (3 items): (Healthy Eating Intentions) had α =.56. Factor 4 (2 items): (Intentions towards Alcohol) had α =-0.16. Although Healthy Eating Intentions α = 0.56 is below the minimum threshold (0.70) for reliability, this is acceptable for a three item scale.[34] The intention toward alcohol factor had two items with such low reliability (α = -0.16) that they could not be considered a separate factor and were removed. A thirteenth EFA iteration confirmed the factor loadings and reliabilities reported above. Hence the parallel analysis indicated that three factors should be retained. [39] The three factor model accounted for 57.61% of the total explained variance.

Table 3 Factor structure of the ABCD Risk Questionnaire

	Components

For peer review only - http://bmjopen.bmj.com/site/about/guidelines.xhtml

	Factor 1 Perceived Risk of Heart Attack / Stroke	Factor 2 Perceived Benefits & Intentions to Change	Factor 3 Healthy Eating Intentions
It is likely that I will suffer from a heart attack or stroke in the future.	.844		
It is likely that I will have a heart attack or stroke some time during my life.	.816		
I feel I will suffer from a heart attack or stroke sometime during my life.	.809		
There is a good chance I will experience a heart attack or stroke in the next 10 years.	.752		
I am not worried that I might have a heart attack or stroke.	.705		
My chances of suffering from a heart attack or stroke in the next 10 years are great.	.687		
It is likely I will have a heart attack or stroke because of my past and/or present behaviours.	.639		
I am concerned about the likelihood of having a heart attack or stroke in the near future.	.575		
I am thinking about exercising at least 2½ hours a week.		.826	
I intend or want to exercise at least 2½ hours a week.		.792	
When I exercise for at least 2½ hours a week I am doing something good for the health of my heart.		.735	
Maintain a healthy weight by exercising at least 2½ hours a week within the next two months.		.658	

For peer review only - http://bmjopen.bmj.com/site/about/guidelines.xhtml

	Components		
	Factor 1 Perceived Risk of Heart Attack / Stroke	Factor 2 Perceived Benefits & Intentions to Change	Factor 3 Healthy Eating Intentions
I am not thinking about exercising for 2 ½ hours a week.		.656	
When I eat at least five portions of fruit and vegetables a day I am doing something good for the health of my heart.		.642	
Increasing my exercise to at least 2½ hours a week will I decrease my chances of having a heart attack or stroke.		.557	
Eat at least five portions of fruit and vegetables per day within the next two months.			.830
I am thinking about eating at least five portions of fruit and vegetables a day.			.772
I am not thinking about eating at least five portions of fruit and vegetables a day.			.731
Note: Factor loadings and commonalities are reported following an EFA using Principal Component Analysis with Varimax rotation.			

The EFA revealed three scales: Perceived Risk of Heart Attack / Stroke, Perceived Benefits and Intentions to Change and Healthy Eating Intentions. A fourth scale assessing Knowledge of CVD Risk and Prevention (not entered into EFA) was added back to the questionnaire following EFA (see Figure 2). Hence the final questionnaire included 26 items grouped into four scales: Knowledge of CVD Risk and Prevention (8 items), Perceived Risk of Heart Attack/Stroke (7 items), Perceived Benefits and Intention to Change Behaviour (7 items) and Healthy Eating Intentions (3 items). In the resulting 26 item questionnaire, two items were changed from questions "How confident are you that you know how to or can…" to statements of agreement "I am confident that I can" so as to be answered using the same Likert scale. The ABCD Risk Questionnaire with a scoring guide for each scale is reported in Appendix D.

DISCUSSION

For peer review only - http://bmjopen.bmj.com/site/about/guidelines.xhtml

To the best of our knowledge this is the first study that describes the development of a short, validated questionnaire examining CVD risk awareness among the NHS Health Check attendees. Satisfactory content and face validity as well as reliability of the ABCD Risk Questionnaire suggests that the tool performs well. It may be used for evaluating the accuracy of perceived CVD risk, general knowledge of CVD and intention to change behaviour in regards to diet and exercise. Administering the ABCD Risk Questionnaire to NHS Health Check attendees may establish the effectiveness of communicating CVD risk to patients and motivating them to change behaviour. The accuracy of perceived CVD risk may be obtained by comparing the responses to questions in the Perceived Risk of Heart Attack / Stroke scale against clinical records indicating predicted CVD risk. General knowledge of CVD may be assessed by examining the responses to questions in the Knowledge of CVD Risk and Prevention scale. Intention to modify diet and exercise may be evaluated using Perceived Benefits and Intentions to Change and Healthy Eating Intentions scale. If NHS Health Check recommendations change over time, the ABCD Risk Questionnaire may need to be updated.

Critics of the NHS Health Check programme point to the lack of its evidence base. [43, 44] The majority of evaluations focused on implementation aspects such as coverage and uptake, statin prescribing and measuring clinical outcomes such as new diagnoses and CVD risk factor reduction. [16, 45-49] As there was no instrument measuring CVD risk awareness, no studies examined the programme's effectiveness of communicating CVD risk to patients. CVD risk presentation was shown to increase the accuracy of perceived risk by 10%. When risk information is repeated this leads to small but significant reductions in predicted CVD risk.[17] A national study showed modest reductions in 10 year predicted CVD risk among NHS Health Check attendees in the first four years.[16] An important limitation of using predicted ten year risk of CVD is the under-estimation of CVD risk among women and younger people. Modest reductions in predicted 10 year risk among women and younger people may translate into major reductions in lifetime risk of CVD.[50] Basing the programme's value solely on its potential to reduce predicted CVD risk in the near term may underrate the programme and lead to its demise. More research is needed to establish the effectiveness of the programme in increasing Health Check attendees' awareness of CVD risk and its impact on predicted lifetime CVD risk.

The ABCD Risk Questionnaire was developed and tested on a non-risk stratified population as the NHS Health Check programme is administered to all eligible people free of vascular disease diagnosis irrespective of their level of CVD risk. The relatively low proportion of high CVD risk (4.5%) and smokers (18.2%) among the 110 NHS Health Check attendees is reflective of the population that took up the NHS Health Check programme between 2009-2014. [45] Questions on smoking and drinking were progressively eliminated as they did not apply to the majority of the study participants. As questions on diet and exercise pertained to all people regardless of their level of CVD risk, such questions that reliably distinguished between study participants were selected for inclusion.

As the ABCD Risk Questionnaire was developed using both an expert panel and a patient focus group, the questions ought to be relatively easy to understand for both patients and clinicians. However a possible limitation to face validity is that the patient focus group evaluating the 69 item questionnaire was not representative of the target population. Whereas the NHS Health Check programme is administered to both men and women, the focus group consisted only of women. Furthermore as these women had postgraduate education and worked in a health related field, they

For peer review only - http://bmjopen.bmj.com/site/about/guidelines.xhtml

may have had higher health literacy than the general population eligible for the NHS Health Check programme. Nonetheless the study population that completed the 65 item questionnaire was not limited to women and had a large proportion of deprived individuals. Since higher levels of deprivation are partly due to having less education,[42] questionnaire development was not limited to people with higher education. Furthermore since population risk factors among the 110 Health Check attendees were in line with those reported among Health Check attendees in a national study,[45] the questionnaire was tested on individuals who were representative of the target population.

Additional studies should be conducted with larger samples to confirm the reliability and validity of the questionnaire. It would be useful to replicate the factor analytic process on an independent, larger sample to confirm the generalizability of these findings.[37]

CONCLUSIONS

The ABCD Risk Questionnaire showed evidence of satisfactory reliability and validity, is brief and easy to use. By capturing patients' views on CVD risk awareness during an NHS Health Check consultation, the questionnaire can be used to evaluate the effectiveness of conveying important information to patients. Clinicians administering the questionnaire to patients may help to establish whether the programme is effective in empowering patients to make informed lifestyle choices about their health.

References

1. World Health Organisation. Prevention of Cardiovascular Disease - Guidelines for assessment and management of cardiovascular risk, 2007. http://www.who.int/cardiovascular_diseases/guidelines/Full%20text.pdf (accessed 08 March 2015)
2. Murray CJL, Richards MA, Newton JN, et al. UK health performance: findings of the Global Burden of Disease Study 2010. *The Lancet*. 2013; 381: 997-1020.
3. Barton GR, Goodall M, Bower P, Woolf S, Capewell S and Gabbay MB. Increasing heart-health lifestyles in deprived communities: economic evaluation of lay health trainers. *Journal of Evaluation in Clinical Practice*. 2012; 18: 835-40.
4. Yusuf S, Hawken S, Ôunpuu S, et al. Effect of potentially modifiable risk factors associated with myocardial infarction in 52 countries (the INTERHEART study): case-control study. *The Lancet*. 2004; 364: 937-52.
5. Bajekal M, Scholes S, Love H, et al. Analysing Recent Socioeconomic Trends in Coronary Heart Disease Mortality in England, 2000–2007: A Population Modelling Study. *PLoS Med*. 2012; 9: e1001237.
6. The National Institute for Health and Care Excellence (NICE). Services for the prevention of cardiovascular disease - NICE commissioning guides [CMG45], 2012. ttps://www.nice.org.uk/guidance/cmg45/chapter/commissioning-services-for-the-prevention-of-cardiovascular-disease (accessed 12 June 2015)
7. Department of Health. Putting prevention first- vascular checks: risk assessment and management - next steps guidance for primary care trusts, 2009. http://www.dh.gov.uk/en/Publicationsandstatistics/Publications/PublicationsPolicyAndGuidance/DH_090277 (accessed 7 July 2016).

For peer review only - http://bmjopen.bmj.com/site/about/guidelines.xhtml

8. Public Health England. NHS Health Check Best Practice Guidance 2015, 2015. http://www.healthcheck.nhs.uk/commissioners_and_healthcare_professionals/national_guidance/ (accessed 6 March 2015)
9. Public Health England. NHS Health Check: Our Approach to the Evidence, 2013. https://www.gov.uk/government/uploads/system/uploads/attachment_data/file/224537/NHS_Health_Check_our_approach_to_the_evidence_v2.pdf;(accessed 12 June 2015).
10. Department of Health. Vascular Checks: Economic Modelling for Vascular Checks, 2008. http://www.dh.gov.uk/en/Publicationsandstatistics/Publications/PublicationsPolicyAndGuidance/DH_085869 (accessed 12 June 2015).
11. Homko CJ, Santamore WP, Zamora L, et al. Cardiovascular Disease Knowledge and Risk Perception Among Underserved Individuals at Increased Risk of Cardiovascular Disease. *Journal of Cardiovascular Nursing*. 2008; 23: 332-7
12. Collins GS and Altman DG. Predicting the 10 year risk of cardiovascular disease in the United Kingdom: independent and external validation of an updated version of QRISK2. *BMJ*. 2012; 344.
13. Webster R and Heeley E. Perceptions of risk: understanding cardiovascular disease. *Risk management and healthcare policy*. 2010; 3: 49-60.
14. Kirkland SA, MacLean DR, Langille DB, Joffres MR, MacPherson KM and Andreou P. Knowledge and awareness of risk factors for cardiovascular disease among Canadians 55 to 74 years of age: results from the Canadian Heart Health Surveys, 1986-1992. *CMAJ : Canadian Medical Association journal = journal de l'Association medicale canadienne*. 1999; 161: S10-6.
15. Potvin L, Richard L and Edwards AC. Knowledge of cardiovascular disease risk factors among the Canadian population: relationships with indicators of socioeconomic status. *CMAJ : Canadian Medical Association journal = journal de l'Association medicale canadienne*. 2000; 162: S5-11.
16. Chang KC, Lee JT, Vamos EP, et al. Impact of the National Health Service Health Check on cardiovascular disease risk: a difference-in-differences matching analysis. *CMAJ : Canadian Medical Association journal = journal de l'Association medicale canadienne*. 2016.
17. Sheridan SL, Viera AJ, Krantz MJ, et al. The Effect of Giving Global Coronary Risk Information to Adults: A Systematic Review. *Archives of Internal Medicine*. 2010; 170: 230-9.
18. Bergman HE, Reeve BB, Moser RP, Scholl S and Klein WMP. Development of a Comprehensive Heart Disease Knowledge Questionnaire. *American Journal of Health Education*. 2011; 42: 74-87.
19. Ammouri AA and Neuberger G. The Perception of Risk of Heart Disease Scale: development and psychometric analysis. *J Nurs Meas*. 2008; 16: 83-97.
20. Tovar EG, Rayens MK, Clark M and Nguyen H. Development and psychometric testing of the Health Beliefs Related to Cardiovascular Disease Scale: preliminary findings. *Journal of advanced nursing*. 2010; 66: 2772-84.
21. Strecher V.J. R, I.M. *The Health Belief Model. In Health Behavior and Health Education, Theory, Research and Practice (Glanz, K., Lewis, F.M., Rimer, B.K. eds)*. San Francisco: Jossey-Bass, 1997.
22. Prochaska JO, Redding, C.A., Evers, K.E. . *The Transtheoretical Model and Stages of Change. In Health Behavior and Health Education, Theory, Research and Practice (Glanz, K., Lewis, F.M., Rimer, B.K. eds).* . San Francisco: Jossey-Bass, 1997.
23. Hsu HY and Gallinagh R. The relationships between health beliefs and utilization of free health examinations in older people living in a community setting in Taiwan. *Journal of advanced nursing*. 2001; 35: 864-73.
24. Prochaska JO, DiClemente CC and Norcross JC. In search of how people change. Applications to addictive behaviors. *Am Psychol*. 1992; 47: 1102-14.
25. Prochaska JO, Norcross JC and DiClemente CC. *Changing for Good: A Revolutionary Six-Stage Program for Overcoming Bad Habits and Moving Your Life Positively Forward.* New York, NY Avon Books, 1994.

For peer review only - http://bmjopen.bmj.com/site/about/guidelines.xhtml

26. Department of Health. Physical Activity Guidelines for Adults (19-64 Years), 2011. http://www.nhs.uk/Livewell/fitness/Documents/adults-19-64-years.pdf (accessed 23 April 2015).
27. Department of Health. Improve Your NHS Health Check Numbers, 2014. http://www.nhs.uk/Conditions/nhs-health-check/Pages/Take-action-to-improve-your-health.aspx (accessed 23 April 2015).
28. Department of Health. Five a Day, 2013. http://www.nhs.uk/livewell/5aday/Pages/ADAYhome.aspx (accessed 23 April, 2015).
29. Andrew S and Halcomb EJ. *Mixed Methods Research for Nursing and the Health Sciences*. Blackwell Publishing Ltd, 2009.
30. Chan CW. Perceptions of coronary heart disease: the development and psychometric testing of a measurement scale. *Psychol Health Med*. 2014; 19: 159-68.
31. Wells KJ, Arevalo M, Meade CD, et al. Development and validation of the biobanking attitudes and knowledge survey (BANKS). *Cancer Epidemiol Biomarkers Prev*. 2014; 23: 374-82.
32. Waltz CF, Strickland OL and Lenz ER. *Measurement in Nursing Research*. Second Edition ed. Philadelphia: F.A. Davis Company, 1991.
33. Waltz CF, Strickland OL and Lenz ER. *Measurement in Nursing and Health Research* Fourth Edition ed. New York: Springer Publishing Company, 2010.
34. Rust J and Golombok S. *Modern psychometrics: The science of psychological assessment.* Second Edition ed. London: Routledge, 1999.
35. Torres L. Initial Development and Validation of the Mexican Intercultural Competence Scale. *Journal of Latina/o psychology*. 2013; 1: 171-81.
36. Nunnally JC and Bernstein IH. *Psychometric theory*. Third Edition ed. New York: McGraw-Hill Higher, INC, 1994.
37. DeVellis RF. *Scale Development Theory and Applications*. Third Edition ed. Los Angelos / London / New Delhi / Singapore / Washington DC: SAGE, 2012.
38. Aldridge V, Wade A and Koutoumanou E. Validity and Reliability University College London, Institute of Child Health2015.
39. Ferguson E and Cox T. Exploratory Factor Analysis: A Users'Guide. *International Journal of Selection and Assessment*. 1993; 1: 84-94.
40. Zwick WR and Velicer WF. Comparison of five rules for determining the number of components to retain. *Psychological Bulletin*. 1986; 99: 432-42.
41. Gray-Donald K, O'Loughlin J, Richard L and Paradis G. Validation of a short telephone administered questionnaire to evaluate dietary interventions in low income communities in Montreal, Canada. *J Epidemiol Community Health*. 1997; 51: 326-31.
42. Department for Communities and Local Government. The English Indices of Deprivation 2010 - Neighborhoods Statistical Release, 2011. https://www.gov.uk/government/uploads/system/uploads/attachment_data/file/6871/1871208.pdf (accessed 16 December 2014).
43. Krogsbøll Lasse T, Jørgensen Karsten J, Grønhøj Larsen C and Gøtzsche Peter C. General health checks in adults for reducing morbidity and mortality from disease. *Cochrane Database of Systematic Reviews*: (2012).
44. Capewell S and Graham H. Will cardiovascular disease prevention widen health inequalities? *PLoS Med*. 2010; 7: e1000320.
45. Chang KC-M, Soljak M, Lee JT, et al. Coverage of a national cardiovascular risk assessment and management programme (NHS Health Check): Retrospective database study. *Preventive Medicine*.
46. Artac M, Dalton ARH, Majeed A, Car J and Millett C. Effectiveness of a national cardiovascular disease risk assessment program (NHS Health Check): Results after one year. *Preventive Medicine*. 2013.
47. Artac M, Dalton ARH, Majeed A, Car J, Huckvale K and Millett C. Uptake of the NHS Health Check programme in an urban setting. *Family Practice*. 2013; 30: 426-35.

For peer review only

For peer review only - http://bmjopen.bmj.com/site/about/guidelines.xhtml

48. Dalton ARH, Bottle A, Okoro C, Majeed A and Millett C. Uptake of the NHS Health Checks programme in a deprived, culturally diverse setting: cross-sectional study. *J Public Health (Oxf)*. 2011; 33: 422-9.
49. Robson J, Dostal I, Sheikh A, et al. The NHS Health Check in England: an evaluation of the first 4 years. *BMJ Open*. 2016; 6: e008840.
50. JBS 3 Joint British Societies for the Prevention of Cardiovascular Disease. Lifetime Risk, 2014. http://www.jbs3risk.com/pages/lifetime_risk.htm (accessed 9 August 2016).

For peer review only

For peer review only - http://bmjopen.bmj.com/site/about/guidelines.xhtml

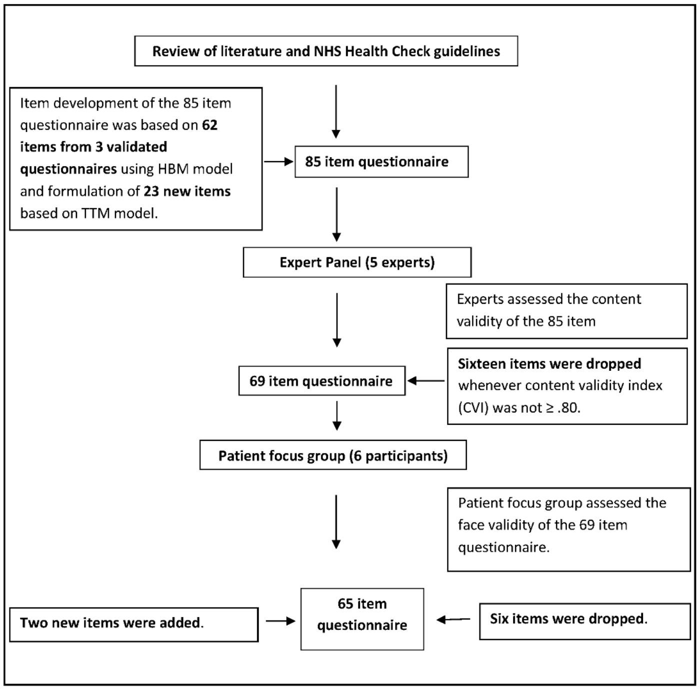

210x297mm (300 x 300 DPI)

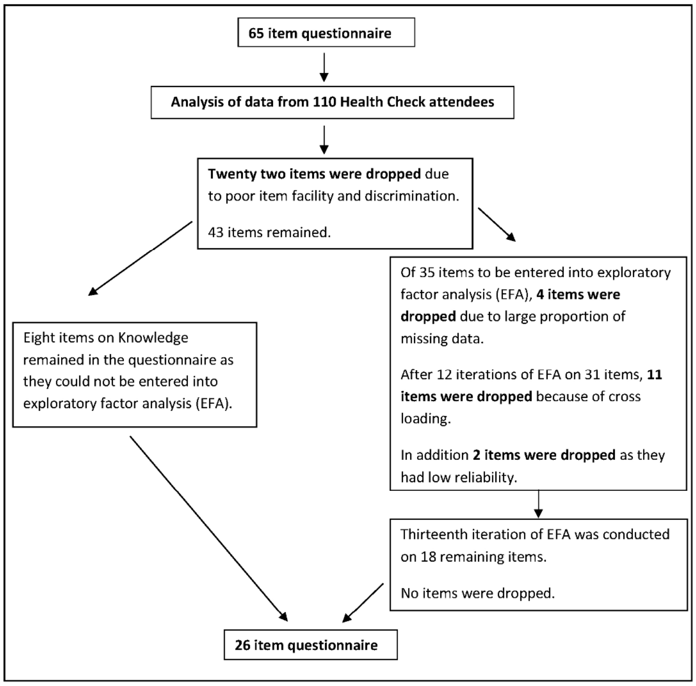

210x297mm (300 x 300 DPI)

Appendix A: 85 Item Questionnaire

Subscale	Items	Answers
Knowledge of CVD Risk and Prevention	1. Eating a lot of red meat increases heart attack and stroke risk.	True, False, Don't Know<br>T=True F=False<br>Correct Answers<br>Q1=T Q2=F Q3=T<br>Q4=F Q5=T Q6=F<br>Q7=F Q8=T Q9=T<br>Q10=T Q11=F<br>Q12=T Q13=T<br>Q14=T Q15=T<br>Q16=F Q17=T<br>Q18=F
	2. Most people can tell whether or not they have high blood pressure.	
	3. You can reduce your risk of heart attack or stroke by being physically active.	
	4. ‘High’ blood pressure is defined as 110/80 (systolic/diastolic) or higher.	
	5. Dietary fibre lowers blood cholesterol.	
	6. The most important cause of heart attack and stroke is stress.	
	7. Trans-fats are healthier for the heart than most other kinds of fats.	
	8. Walking and gardening are considered types of exercise that can lower the risk of having a heart attack or stroke.	
	9. You can reduce your chance of developing a heart attack or stroke by eating five-a-day diet of fruits and vegetables.	
	10. Moderate physical activity of 150 minutes a week will reduce your chances of developing a heart or stroke.	
	11. People who quit smoking by 60 add five years to their life.	
	12. People who have diabetes are at higher risk having a heart attack or stroke.	
	13. Managing your stress levels will help you to manage your blood pressure.	
	14. HDL refers to ‘good’ cholesterol, and LDL refers to ‘bad’ cholesterol.	
	15. The healthiest exercise for the heart involves rapid breathing for a sustained period of time.	
	16. Many vegetables are high in cholesterol.	
	17. You are more likely to have a heart attack or stroke if you’re overweight or obese.	
	18. Drinking alcohol has nothing to do with reducing the risk of heart attack or stroke.	
Perceived Risk and Vulnerability of CVD	19. There is a possibility that I will have a heart attack or stroke.	1 = Strongly disagree; 2 = disagree; 3 = agree; 4 = strongly agree
	20. There is a good chance I will experience a heart attack or stroke during the next 10 years.	
	21. A person who gets a heart attack or stroke has no chance of recovering.	
	22. I have a high chance of getting a heart attack or stroke because of my past behaviours.	
	23. I feel sure that I will have a heart attack or stroke.	
	24. Healthy lifestyle habits are unattainable.	
	25. It is likely that I will get a heart attack or stroke.	
	26. I am at risk for having a heart attack or stroke.	
	27. It is possible that I will have a heart attack or stroke.	
	28. I am not doing anything now that is unhealthy to my heart.	
	29. I am too young to have a heart attack or stroke.	
	30. People like me do not get a heart attack or stroke.	
	31. I am very healthy so I will not have a heart attack or stroke.	
	32. I am not worried that I might have a heart attack or stroke.	
	33. People my age are too young to have a heart attack or stroke.	

For peer review only

	34. People my age do not have a heart attack or stroke.	
	35. My lifestyle habits do not put me at risk for having a heart attack or stroke.	
	36. No matter what I do, if I am going to have a heart attack or stroke, I will have one.	
	37. People who do not have a heart attack or stroke are just plain lucky.	
	38. The causes of a heart attack or stroke are unknown.	
Perceived Susceptibility	39. It is likely that I will suffer from a heart attack or stroke in the future.	
	40. My chances of suffering from a heart attack or stroke in the next few years are great.	
	41. Having a heart attack or stroke is currently a possibility for me.	
	42. I feel I will suffer from a heart attack or stroke sometime during my life.	
	43. I am concerned about the likelihood of having a heart attack or stroke in the near future.	
Perceived Severity	44. Heart attacks and strokes are always fatal.	
	45. Having a heart attack or stroke will threaten my relationship with my significant other.	
	46. My whole life would change if I had a heart attack or stroke.	
	47. Having a heart attack or stroke would have a very bad effect on my sex life.	
	48. If I have a heart attack or stroke I will die within 10 years.	
Perceived Benefits	49. Increasing my exercise will decrease my chances of having a heart attack or stroke.	
	50. Eating a healthy diet will decrease my chance of having a heart attack or stroke.	
	51. Stopping smoking will reduce my chance of having a heart attack or stroke.	
	52. When I exercise I am doing something good for myself.	
	53. When I eat healthy I am doing something good for myself.	
	54. Cutting down on alcohol will decrease my chances of having a heart attack or stroke.	
Perceived Barriers	55. I do not know appropriate exercises to perform to reduce my risk of developing cardiovascular disease.	
	56. I do not know the recommended drinking limits for men or women.	
	57. I do not have time to exercise for 30 minutes a day on most days of the week.	
	58. I do not know what is considered a healthy diet that would prevent me from developing cardiovascular disease.	
	59. I will not have energy if I stop smoking.	
	60. I cannot afford to buy healthy foods.	
	61. I have other problems more important than worrying about diet and exercise.	
Self Efficacy	62. How confident are you that you know or can control the risks of having a heart attack or stroke.	1= not at all confident, 2=somewhat confident, 3= moderately confident, 4=very confident,
	63. How confident are you that you know or can maintain a healthy weight by exercising regularly.	
	64. How confident are you that you know or can stop smoking if you want to.	
	65. How confident are you that you know or can consume less alcohol.	

For peer review only

For peer review only - http://bmjopen.bmj.com/site/about/guidelines.xhtml

	66. How confident are you that you know or can control your blood pressure and/or cholesterol levels by taking your prescribed medications.	5=completely confident
	67. How confident are you that you know or can eat a healthy and balanced diet.	
Intention to Change Behaviour or Cues to Action	68. I want to stop smoking (if you do smoke).	1 = Strongly disagree; 2 = disagree; 3 = agree; 4 = strongly agree
	69. I intend to maintain a healthy weight.	
	70. I intend to be physically active within two months.	
	71. I expect to maintain a healthy weight.	
	72. I want to be physically active.	
	73. I intend to eat a healthy and balanced diet within two months.	
	74. I expect to stop smoking (if you do smoke).	
	75. I want to cut down on alcohol.	
	76. I want to maintain a healthy and balanced diet.	
	77. I intend to stop smoking within two months (if you do smoke).	
	78. I expect to eat a healthy and balanced diet.	
	79. I intend to cut down on alcohol in the next two months.	
	80. I expect to be physically active.	
	81. I expect to cut down on alcohol.	
	82. I want to eat a healthy and balanced diet.	
	83. I expect to take my medication to control my blood pressure and/or cholesterol	
	84. I want to take my medication to control my blood pressure and/or cholesterol	
	85. I intend to take my medication to control my blood pressure and/or cholesterol within two months	

For peer review only

Appendix B: 65 Item Questionnaire

Scale	Subscale	Items	Coding of Answers
**Knowledge**<br><br>(15 items)<br><br>Higher sum score = more knowledgeable / more correct	**CVD Risk Knowledge – Risk of having a heart attack / stroke**<br><br>(15 items)	1. Eating a lot of red meat increases heart attack and stroke risk.	Correct Answers<br><br>Q1-T Q6-T Q11-F<br>Q2-F Q7-T Q12-T<br>Q3-T Q8-T Q13-T<br>Q4-T Q9-T Q14-T<br>Q5-T Q10-T Q15-F<br><br>T= True<br>F= False<br><br>Correct: Score = 1, Incorrect or Don't Know Score = 0.
		2. Most people can tell whether or not they have high blood pressure.	
		3. You can reduce your risk of heart attack or stroke by being physically active.	
		4. One of the main causes of heart attack and stroke is stress.	
		5. Walking and gardening are considered types of exercise that can lower the risk of having a heart attack or stroke.	
		6. You can reduce your chance of developing a heart attack or stroke by eating at least five portions of fruit and vegetables a day.	
		7. Moderate intensity activity of 2 ½ hours a week will reduce your chances of developing a heart attack or stroke.	
		8. People who have diabetes are at higher risk of having a heart attack or stroke.	
		9. Managing your stress levels will help you to manage your blood pressure.	
		10. The healthiest exercise for the heart involves rapid breathing for 10 minutes or more.	
		11. Many vegetables are high in cholesterol.	
		12. You are more likely to have a heart attack or stroke if you're overweight or obese.	
		13. Drinking high levels of alcohol can increase your cholesterol and triglyceride levels.	
		14. HDL refers to 'good' cholesterol, and LDL refers to 'bad' cholesterol.	
		15. Family history of heart disease is not a risk	

For peer review only - http://bmjopen.bmj.com/site/about/guidelines.xhtml

		factor for high blood pressure.	
**Perceived CVD Risk** (15 items)<br><br>Composite score = sum across subscales. Higher score = higher perception of risk of having a heart attack or stroke	**Dread Risk** (7 items)	16. There is a possibility that I will have a heart attack or stroke.	Higher sum score = Higher perceived lack of control, dread, catastrophic potential and fatal consequences<br><br>1 = Strongly disagree; 2 = disagree; 3 = agree; 4 = strongly agree; N/A = 0
		17. There is a good chance I will experience a heart attack or stroke in the next 10 years.	
		18. It is likely I will have a heart attack or stroke because of my past and/or present behaviours.	
		19. I feel sure that I will have a heart attack or stroke.	
		20. It is likely that I will have a heart attack or stroke some time during my life.	
		21. I am at risk for having a heart attack or stroke some time during my life.	
		22. It is possible that I will have a heart attack or stroke within the next 10 years.	
	**Risk** (3 items)	23. I am too young to have a heart attack or stroke.	Higher sum score = Higher perceived hazards that has few, moderate, known outcomes and consequences<br><br>**Reverse coded**<br>4 = Strongly disagree; 3 = disagree; 2 = agree; 1 = strongly agree; N/A = 0
		24. People like me do not have a heart attack or stroke.	
		25. I am not worried that I might have a heart attack or stroke.	
	**Unknown Risk** (5 items)	26. I am not doing anything now that is unhealthy to my heart.	Higher sum score = Higher perceived hazards judged to be unobservable, unknown, new, and delayed in their manifestation of harm<br><br>**Reverse coded**<br>4 = Strongly disagree; 3 = disagree; 2 = agree; 1 = strongly agree; N/A = 0
		27. I am very healthy so I will not have a heart attack or stroke.	
		28. My lifestyle habits do not put me at risk for having a heart attack or stroke.	
		29. No matter what I do, if I am going to have a heart attack or stroke, I will have one.	
		30. People who do not have a heart attack or stroke are just plain lucky.	

For peer review only - http://bmjopen.bmj.com/site/about/guidelines.xhtml

**CVD Health Beliefs** (13 items)	**Susceptibility** (4 items)	31. It is likely that I will suffer from a heart attack or stroke in the future.	Higher average score = Higher perceived personal risk of heart attack and stroke<br><br>1 = Strongly disagree; 2 = disagree; 3 = agree; 4 = strongly agree; N/A = 0
		32. My chances of suffering from a heart attack or stroke in the next 10 years are great.	
		33. I feel I will suffer from a heart attack or stroke sometime during my life.	
		34. I am concerned about the likelihood of having a heart attack or stroke in the near future.	
	**Severity** (3 items)	35. Heart attacks and strokes are always fatal.	Higher average score = Higher perceived severity of heart attack and stroke<br><br>1 = Strongly disagree; 2 = disagree; 3 = agree; 4 = strongly agree; N/A = 0
		36. My whole life would change if I had a heart attack or stroke.	
		37. If I have a heart attack or stroke I will die within 10 years.	
	**Benefits** (4 items)	38. Eating at least five portions of fruit and vegetables a day will decrease my chances of having a heart attack or stroke.	Higher average score = Higher perceived benefits of diet, exercise, consuming less alcohol and smoking cessation for reducing risk for heart attack and stroke<br><br>**Reverse coded**<br>4 = Strongly disagree; 3 = disagree; 2 = agree; 1 = strongly agree; N/A = 0
		39. Increasing my exercise to at least 2½ hours a week will decrease my chances of having a heart attack or stroke.	
		40. When I exercise for at least 2½ hours a week I am doing something good for the health of my heart.	
		41. When I eat at least five portions of fruit and vegetables a day I am doing something good for the health of my heart.	
	**Barriers** (2 items)	42. I do not have time to exercise on most days of the week.	Higher average score = Higher perception of select barriers to engaging in heart attack and stroke risk reducing behaviours<br><br>1 = Strongly disagree; 2 = disagree; 3 = agree; 4 = strongly agree; N/A = 0
		43. I cannot afford to buy healthy foods.	
**Self**	**CVD risk**	How confident are you that you know how to or can…	Higher average score = higher perceived

For peer review only - http://bmjopen.bmj.com/site/about/guidelines.xhtml

Efficacy<br><br>(5 items)	reduction<br><br>self efficacy<br><br>(5 items)	44. Control the risks of having a heart attack or stroke.	confidence<br><br>1 = Not at all confident; 2 = somewhat confident; 3 = very confident; 4 = completely confident; N/A = 0
		45. Maintain a healthy weight by exercising at least 2½ hours a week within the next two months.	
		46. Stop smoking if you want to.	
		47. Drink within the recommended levels of alcohol.	
		48. Eat at least five portions of fruit and vegetables per day within the next two months.	
**Intention / Readiness to Change**<br><br>(17 items)	**Exercise**<br><br>(4 items)	49. I am not thinking about exercising for 2 ½ hours a week.	Higher average score = Higher perceived readiness for change with regard to exercise behaviour<br><br>1 = Strongly disagree; 2 = disagree; 3 = agree; 4 = strongly agree; N/A = 0
		50. I am thinking about exercising at least 2½ hours a week.	
		51. I intend or want to exercise at least 2½ hours a week.	
		52. I am ready or have started to exercise 2 ½ hours a week.	
	**Diet**<br><br>(4 items)	53. I am not thinking about eating at least five portions of fruit and vegetables a day.	Higher average score = Higher perceived readiness for change with regard to health dietary behaviour<br><br>1 = Strongly disagree; 2 = disagree; 3 = agree; 4 = strongly agree; N/A = 0
		54. I am thinking about eating at least five portions of fruit and vegetables a day.	
		55. I intend or want to eat at least five portions of fruit and vegetables a day.	
		56. I am ready or started to eat at least five portions of fruit and vegetables a day.	
	**Alcohol**<br><br>(4 items)	57. I am thinking about cutting down on alcohol.	Higher average score = Higher perceived readiness for change with regard to alcohol consumption behaviour<br><br>1 = Strongly disagree; 2 = disagree; 3 = agree; 4 = strongly agree; N/A = 0
		58. I intend or want to cut down on alcohol.	
		59. I have been cutting down on alcohol.	
		60. I am not thinking about cutting down on alcohol.	
	**Smoking**<br><br>(5 items)	61. I am thinking of stopping smoking within two months.	Higher average score = Higher perceived readiness for change with regard to smoking cessation

For peer review only - http://bmjopen.bmj.com/site/about/guidelines.xhtml

		62. I have reduced or stopped smoking.	behaviour<br><br>1 = Strongly disagree; 2 = disagree; 3 = agree; 4 = strongly agree; N/A = 0
		63. I intend or want to stop smoking.	
		64. If I stop smoking it will reduce my chances of having a heart attack or stroke.	
		65. I am not thinking about stopping smoking.	

For peer review only

For peer review only - http://bmjopen.bmj.com/site/about/guidelines.xhtml

Appendix C. Population characteristics of 110 NHS Health Check attendees

Population Characteristics		n	% Total
Gender	Male	51	46.4%
	Female	56	50.9%
Age group	40-49	45	40.9%
	50-59	40	36.4%
	60-74	14	12.7%
Ethnicity	White	93	84.5%
	Mixed	2	1.8%
	Asian	2	1.8%
	Black	4	3.6%
	Other	4	3.6%
*Deprivation**	IMD 1 - least deprived	14	12.7%
	IMD 2	30	27.3%
	IMD 3	12	10.9%
	IMD 4	31	28.2%
	IMD 5 - most deprived	14	12.7%
Cholesterol	Raised total cholesterol TC≥ 5 mmol/l	66	60.0%
Blood pressure	High blood pressure BP ≥ 140/90 mm Hg	28	25.5%
Body Mass Index (BMI)	Obese (BMI>=30)	26	23.6%
Physical activity	Physically inactive	22	20.0%
Smoking status	Smokers	20	18.2%
Alcohol consumption	Excessive drinkers	13	11.8%
*10 year predicted risk of CVD***	High CVD Risk (QRisk2>=20%)	5	4.5%
	Medium CVD Risk (Qrisk2 10-20%)	21	19.1%
	Low CVD Risk (QRisk2<10%)	85	77.3%

***Deprivation was measured using the Index of Multiple Deprivation (IMD).

**Ten year predicted risk of CVD was estimated using the Q-Risk 2 algorithm[1]

References

1. Hippisley-Cox J, Coupland C, Vinogradova Y, et al. Predicting cardiovascular risk in England and Wales: prospective derivation and validation of QRISK2. *BMJ*. 2008; 336.

For peer review only - http://bmjopen.bmj.com/site/about/guidelines.xhtml

Appendix D

The ABCD Risk Questionnaire and scoring guide

Scale	Items	Coding
**Knowledge**<br><br>Higher sum score = more knowledgeable / more correct about having a heart attack or stroke	1. One of the main causes of heart attack and stroke is stress.<br><br>2. Walking and gardening are considered types of exercise that can lower the risk of having a heart attack or stroke.<br><br>3. Moderately intense activity of 2 ½ hours a week will reduce your chances of having a heart attack or stroke.<br><br>4. People who have diabetes are at higher risk of having a heart attack or stroke.<br><br>5. Managing your stress levels will help you to manage your blood pressure.<br><br>6. Drinking high levels of alcohol can increase your cholesterol and triglyceride levels.<br><br>7. HDL refers to 'good' cholesterol, and LDL refers to 'bad' cholesterol.<br><br>8. A family history of heart disease is not a risk factor for high blood pressure.	Correct Answers:<br><br>Q1-T<br><br>Q2-T<br><br>Q3-T<br><br>Q4-T<br><br>Q5-T<br><br>Q6-T<br><br>Q7-T<br><br>Q8-F<br><br>T= True<br>F= False<br><br>Correct: Score = 1,<br>Incorrect or Don't Know: Score = 0.
**Perceived Risk of**<br><br>**Heart**<br><br>**Attack/Stroke**<br><br>Higher sum score = higher perception of risk of having a heart attack or stroke	9. I feel I will suffer from a heart attack or stroke sometime during my life.	1=Strongly disagree; 2 = disagree; 3 = agree; 4 = strongly agree; N/A = 0
	10. It is likely that I will suffer from a heart attack or stroke in the future.	1=Strongly disagree; 2 = disagree; 3 = agree; 4 = strongly agree; N/A = 0
	11. It is likely that I will have a heart attack or stroke some time during my life.	1=Strongly disagree; 2 = disagree; 3 = agree; 4 = strongly agree; N/A = 0
	12. There is a good chance I will experience a heart attack or stroke in the next 10 years.	1=Strongly disagree; 2 = disagree; 3 = agree; 4 = strongly agree; N/A = 0
	13. My chances of suffering from a heart attack or stroke in the next 10 years are great.	1=Strongly disagree; 2 = disagree; 3 = agree; 4 = strongly agree; N/A = 0
	14. It is likely I will have a heart attack or stroke because of my past and/or present	1=Strongly disagree; 2 = disagree; 3 = agree; 4 = strongly agree; N/A = 0

For peer review only

Scale	Items	Coding
**Perceived Risk of Heart Attack/Stroke**	behaviours.	
	15. I am not worried that I might have a heart attack or stroke.	**Reverse coded**<br>4=Strongly disagree; 3 = disagree; 2 = agree; 1 = strongly agree; N/A = 0
	16. I am concerned about the likelihood of having a heart attack or stroke in the near future.	1=Strongly disagree; 2 = disagree; 3 = agree; 4 = strongly agree; N/A = 0
**Perceived Benefits and Intentions to Change**<br>Higher average score = Higher perceived benefits of diet and exercise and higher perceived readiness for change in regards to exercise behaviour	17. I am thinking about exercising at least 2½ hours a week.	1=Strongly disagree; 2 = disagree; 3 = agree; 4 = strongly agree; N/A = 0
	18. I intend or want to exercise at least 2½ hours a week.	1=Strongly disagree; 2 = disagree; 3 = agree; 4 = strongly agree; N/A = 0
	19. When I exercise for at least 2½ hours a week I am doing something good for the health of my heart.	1=Strongly disagree; 2 = disagree; 3 = agree; 4 = strongly agree; N/A = 0
	20. I am confident that I can maintain a healthy weight by exercising at least 2½ hours a week within the next two months.	1=Strongly disagree; 2 = disagree; 3 = agree; 4 = strongly agree; N/A = 0
	21. I am not thinking about exercising for 2 ½ hours a week.	**Reverse coded**<br>4=Strongly disagree; 3 = disagree; 2 = agree; 1 = strongly agree; N/A = 0
	22. When I eat at least five portions of fruit and vegetables a day I am doing something good for the health of my heart.	1=Strongly disagree; 2 = disagree; 3 = agree; 4 = strongly agree; N/A = 0
	23. Increasing my exercise to at least 2½ hours a week will decrease my chances of having a heart attack or stroke.	1=Strongly disagree; 2 = disagree; 3 = agree; 4 = strongly agree; N/A = 0
**Healthy Eating Intentions**<br>Higher average score = Higher perceived readiness for change with regard to health dietary behaviour	24. I am confident that I can eat at least five portions of fruit and vegetables per day within the next two months.	1=Strongly disagree; 2 = disagree; 3 = agree; 4 = strongly agree; N/A = 0
	25. I am thinking about eating at least five portions of fruit and vegetables a day.	1=Strongly disagree; 2 = disagree; 3 = agree; 4 = strongly agree; N/A = 0
	26. I am not thinking about eating at least five portions of fruit and vegetables a day.	**Reverse coded**<br>4=Strongly disagree; 3 = disagree; 2 = agree; 1 = strongly agree; N/A = 0

For peer review only - http://bmjopen.bmj.com/site/about/guidelines.xhtml

Scale	Items	Coding

The Attitudes and Beliefs about Cardiovascular Disease (ABCD) Risk Questionnaire is licensed under the Creative Commons Attribution-NonCommercial-NoDerivatives 4.0 International License.

For peer review only

Development of a tool to evaluate the effectiveness of National Health Service Health Check cardiovascular disease risk assessment: a questionnaire examining patients' awareness of risk

Journal:	*BMJ Open*
Manuscript ID	bmjopen-2016-014413.R1
Article Type:	Research
Date Submitted by the Author:	03-May-2017

Complete List of Authors:	Woringer, Maria; Imperial College London Department of Primary Care and Public Health, 3rd Floor Reynolds Building Charing Cross Campus St Dunstans Road, W6 8RP<br>Nielsen, Jessica; City University London, School of Arts and Social Sciences, Department of Psychology, City University London Northampton Square, EC1V 0HB<br>Zibarras, Lara; City University London, School of Arts and Social Sciences, Department of Psychology, City University London Northampton Square, EC1V 0HB<br>Evason, Julie; Health Diagnostics Ltd, Chatham House, Dee Hills Park, CH3 5AR<br>Kassianos, Angelos; University College London, Department of Applied Health Research<br>Harris, Matthew; Imperial College London, Institute of Global Health Innovation, Level 1, Faculty Building South Kensington Campus, SW7 2NA ; Imperial College London Department of Primary Care and Public Health, 3rd Floor Reynolds Building Charing Cross Campus St Dunstans Road, W6 8RP<br>Majeed, Azeem; Imperial College London, Primary Care & Public Health, 3rd Floor Reynolds Building Charing Cross Campus St Dunstans Road, W6 8RP<br>Soljak, Michael; Imperial College London, Primary Care & Public Health, 3rd Floor Reynolds Building Charing Cross Campus St Dunstans Road, W6 8RP

**Primary Subject Heading**:	Communication
Secondary Subject Heading:	General practice / Family practice, Health services research
Keywords:	QUALITATIVE RESEARCH, PREVENTIVE MEDICINE, PRIMARY CARE, PUBLIC HEALTH

SCHOLARONE™ Manuscripts

For peer review only

Development of a tool to evaluate the effectiveness of National Health Service Health Check cardiovascular disease risk assessment: a questionnaire examining patients' awareness of risk

Author Names and Affiliations:

Ms. Maria Woringer (a)

Dr. Jessica Jones Nielsen (b)

Dr. Lara Zibarras (b)

Ms. Julie Evason (c)

Dr. Angelos P. Kassianos (d)

Dr. Matthew Harris (e)

Professor Azeem Majeed (a)

Dr. Michael Soljak (a)

Affiliations :

a) Department of Primary Care and Public Health, Imperial College London

b) Department of Psychology, City University London

c) Health Diagnostics Ltd, Chester, UK

d) Department of Applied Health Research, University College London

e) Institute of Global Health Innovation, Imperial College London

Word Count (excluding title page, declarations, abstract, keywords, strengths and limitations and references): 3,999

Author responsible for correspondences concerning the manuscript:

Maria Woringer; Imperial College London, Department of Primary Care and Public Health, Charing Cross Campus, The Reynolds Building, St Dunstan's Road, London W6 8RP, UK
m.woringer@imperial.ac.uk; Tel: +44(0)2075940789

For peer review only

For peer review only - http://bmjopen.bmj.com/site/about/guidelines.xhtml

List of Declarations:

Dissemination of Study Findings

The work on the development and validation of the patient questionnaire was presented as a poster titled "Development and Validation of the Attitudes and Beliefs about Cardiovascular Disease (ABCD) Risk Survey" at the NHS Health Check 2015 – Improvement through Collaboration conference in Leeds, England on 26 February, 2015. In addition, an abstract titled "Development and validation of a patient survey to assess the effectiveness of cardiovascular disease screening" was selected for oral presentation at the First International Conference of Public Health, Primary Care and Congress of Person Centred Medicine on October 29, 2015 and accepted for publication in the International Journal of Person Centred Medicine. This submission is not under consideration by any other journal. All authors have approved the manuscript and this submission.

Permissions

Favourable ethical opinion for the study - "Patient Evaluation of the NHS Health Check programme to Investigate the Programme's Effectiveness in Communicating CVD Risk" was obtained from the NRES Committee London – City & East reference number 13/LO/1885.

Study participants gave their written informed consent to participate in the study and to share their results and medical data.

The Attitudes and Beliefs about Cardiovascular Disease (ABCD) Risk Questionnaire is licensed under the Creative Commons Attribution-NonCommercial-NoDerivatives 4.0 International License.

Funding

This work was supported by the National Institute for Health Research (NIHR) Diagnostics Evidence Co-operatives (DEC) / Collaboration for Leadership in Applied Health Research and Care (CLAHRC) grant.

Data Sharing Agreement

No additional data available.

Competing Interests

None.

Contributors

MW, AM, MS, and HW designed the study, JE supplied the data. JJN designed the validation instrument, LZ performed the psychometric analysis. JE, AK, MH and AM reviewed the validation instrument's face and content validity. All authors discussed data analyses and interpreted the results. MW wrote the first draft of the manuscript. All authors critically revised and approved the final manuscript. MW had full access to all the data used in the study and takes responsibility for the integrity of the data and the accuracy of the data analysis. MW is the guarantor.

For peer review only

For peer review only - http://bmjopen.bmj.com/site/about/guidelines.xhtml

ABSTRACT

Background

The National Health Service (NHS) Health Check is a CVD risk assessment and management programme in England aiming to increase CVD risk awareness among people at increased risk of CVD. There is no tool to assess the effectiveness of the programme in communicating CVD risk to patients.

Aims

The aim of this paper was to develop a questionnaire examining patients' CVD risk awareness for use in health service research evaluations of the NHS Health Check programme.

Methods

We developed an 85 item questionnaire to determine patients' views of their risk of CVD. The questionnaire was based on a review of the relevant literature. After review by an expert panel and focus group discussion, 22 items were dropped and 2 new items were added. The resulting 65 item questionnaire with satisfactory content validity (content validity indices >=0.80) and face validity was tested on 110 NHS Health Check attendees in primary care in a cross sectional study between May 21 and July 28, 2014.

Results

Following analyses of data, we reduced the questionnaire from 65 to 26 items. The 26 item questionnaire constitutes 4 scales: Knowledge of CVD Risk and Prevention, Perceived Risk of Heart Attack/Stroke, Perceived Benefits and Intention to Change Behaviour and Healthy Eating Intentions. Perceived Risk (Cronbach's α = 0.85) and Perceived Benefits and Intention to Change Behaviour (Cronbach's α = 0.82) have satisfactory reliability (Cronbach's α >=0.70). Healthy Eating Intentions (Cronbach's α = 0.56) is below minimum threshold for reliability but acceptable for a three item scale.

Conclusions

The final questionnaire, with satisfactory reliability and validity, is recommended for use in assessing patients' understanding of CVD risk among NHS Health Check attendees.

Word Count: 276

Keywords: cardiovascular disease, primary prevention, risk assessment, questionnaire

Strengths and limitations of this study

- Questionnaire guided by literature review, expert panel, patient focus group & data analysis
- Largely developed among 110 individuals representative of the target population
- Face validity assessed via a patient focus group not representative of the target population

For peer review only - http://bmjopen.bmj.com/site/about/guidelines.xhtml

INTRODUCTION

Cardiovascular disease (CVD) is a major cause of disability and premature mortality worldwide accounting for a third of deaths annually in England.[1, 2] Ninety per cent of CVD cases are associated with modifiable lifestyle factors.[3, 4] Despite substantial reductions in mortality, modifiable risk factors contributed to only 34% of the overall decline in CVD mortality in England between 2000-2007.[5] Further gains could be made by promoting healthier lifestyle changes. CVD contributes considerably to the rising cost of healthcare and is estimated to cost the NHS and UK economy £30 billion annually. [1] In 2010 / 2011 there were 1.4 million hospital admissions related to CVD of which 60% were for people younger than 75 years of age and more than half were as an emergency. Preventing long term illness and disability associated with CVD is important for improving health while reducing healthcare costs.[6]

The National Health Service (NHS) Health Check programme may be important for preventing the premature onset of disease while reducing healthcare costs associated with CVD by identifying individuals at increased risk of CVD, raising their awareness of CVD risk and helping them manage their risk. [7-10] The NHS Health Check programme is a CVD risk assessment and management programme in England launched by the Department of Health in April 2009.[7] The programme aims to prevent heart disease, stroke, diabetes and kidney disease whilst reducing health inequalities.[8] Eligibility criteria is being 40-74 years old and free of vascular disease diagnosis. All participants are offered general lifestyle advice. People at high risk of CVD are offered statins, lifestyle advice and behaviour change support in relation to physical activity, smoking cessation, safe alcohol consumption and healthy diet. The programme has a potential to prevent 1,600 heart attacks and strokes, 650 premature deaths, and over 4,000 new cases of diabetes each year.[9] Projected programme cost is £180-£243 million/year at full implementation with estimated cost per quality adjusted life year (QALY) being £3,000.[10]

In order to adopt healthy lifestyle behaviours related to diet, exercise, smoking and alcohol consumption, the general population must be aware of CVD risk.[11] In the context of the NHS Health Check Programme, CVD risk awareness refers to the accuracy of perceived risk of CVD against predicted CVD risk, general knowledge of CVD and what one can do to lower predicted CVD risk. Whereas predicted CVD risk refers to one's chance of experiencing a heart attack or stroke,[12] perceived risk of CVD refers to a person's perception of their CVD risk. While as many as 40% of the general population underestimate their CVD risk, 20% overestimate their risk.[13] False reassurance may lead to adoption and or maintenance of unhealthy behaviours contributing to the premature onset of CVD. Low CVD risk awareness is reported among men, inner city residents, and people of lower socioeconomic status. [11 14 15] It is not known whether or not the Health Check results in improved CVD risk awareness.

Although a number of validated questionnaires were developed to measure knowledge, perceptions of CVD or intention to change behaviour,[15-17] no short, validated questionnaire was developed to assess CVD risk awareness using all of these scales. Until now studies examining the accuracy of perceived risk and knowledge of CVD relied on non-validated tools.[16] The problem with using non-validated tools is that the questions may not accurately and reliably capture individuals' views or

For peer review only - http://bmjopen.bmj.com/site/about/guidelines.xhtml

measure what they intend to measure. The aim of this work was to develop a questionnaire with satisfactory face, content validity and reliability to assess the effectiveness of the NHS Health Check programme in raising patients' awareness of CVD risk.

METHODS

The first phase of development of the questionnaire was guided by a literature review, an expert panel and a patient focus group. At each stage of questionnaire development, the number of items was reduced (see Figure 1).

Figure 1 Flowchart of Phase I of Questionnaire Development

The second phase of questionnaire development was guided by an analysis of data from 110 NHS Health Check attendees who completed the 65 item questionnaire. The number of questionnaire items was further reduced (see Figure 2).

Figure 2 Flowchart of Phase II of Questionnaire Development

Phase I of Questionnaire Development

Construction of draft questionnaire by review of relevant literature

We performed an extensive literature review pertaining to CVD risk awareness between December 2013 and January 2014 in the areas of disease knowledge, risk perception, intention to change and self-efficacy related to CVD and the Health Belief Model to guide initial item development. PubMed and PsycINFO databases and Google Scholar Articles were utilised to search for existing instruments that measure perception of CVD risk, CVD knowledge and self-efficacy with no limits on the year of publication. The following key words were used to identify the relevant literature: "cardiovascular disease" "heart disease" " knowledge" "risk" "test" "questionnaire" "scale" "assessment" "self-efficacy" "perception" "health belief model". Questionnaires were considered if they addressed CVD risk awareness, reported moderate to high scores of reliability and validity in population studies and had suitable wording and level of understanding. Questionnaires were excluded if they pertained to individuals under the age of 15 as these people would not be eligible to receive an NHS Health Check, focused on risk unrelated to heart attack or stroke, and were not written in English.

Although a number of questionnaires were found measuring different aspects of CVD risk awareness such as heart disease knowledge, perception of CVD risk, perceived susceptibility and severity of CVD and benefits and barriers to adopting healthy behaviours,[17-19] no single questionnaire encompassed them all. Initial item development was guided by HBM[20] and the Transtheoretical Model (TTM).[21] According to HBM, individuals who have accurate knowledge of CVD and perceived susceptibility to and consequences of the disease, and are aware of the benefits of taking preventive measures are more likely to make important lifestyle choices to prevent the onset of disease.[22] The TTM describes behavioural change as a staged process over time including pre-contemplation, contemplation, preparation, action and maintenance.[21] Sixty five items were selected using validated questionnaires addressing CVD knowledge, and the main constructs of HBM such as perceived susceptibility, perceived severity, perceived benefits of changing behaviours, and perceived barriers to making changes.[17-19] In addition 23 new items were generated to identify perceived levels of

For peer review only - http://bmjopen.bmj.com/site/about/guidelines.xhtml

readiness to engage in CVD risk reduction behaviours (using TTM) and self-efficacy (confidence in ability to change health behaviour) in relation to exercise, diet, smoking cessation and decreasing alcohol consumption.[23, 24] These items were based on data collected during an NHS Health Check and behaviour specific recommendations such as stopping smoking, consuming no more than 14 units of alcohol a week, eating at least five portions of fruit and vegetables a day and exercising at least 150 minutes per week.[25-28] The resulting 85 item questionnaire is in Appendix A.

Modification of questionnaire by expert panel to obtain satisfactory content validity

A panel of experts in the areas of CVD, health psychology, public health, psychometrics and questionnaire development and medicine were asked to evaluate each item and the total 85 item questionnaire for content validity in February 2014. Experts assessed content validity of the questionnaire by examining whether the items were representative of the content they were intended to measure.[29] Items were examined for representatives of the scale domain, appropriateness and relevance. The content validity index (CVI), a widely used technique in scale development determined item and questionnaire clarity, homogeneity, and relevance on a 4-point Likert scale (ranging from 1 = *an irrelevant item* to 4 = *an extremely relevant item*).[30, 31] A CVI of ≥ 0.80 is recommended.[32, 33] Experts were asked the following questions: "*Do these items belong together in the subscale?*" and "*Does each item belong in the set?*" For ratings of content validity, experts were asked whether the subscale definition and label fitted the set of items presented; whether each item belonged with the label and definition; and whether each item was unique in its contribution to the subscale.

Modification of questionnaire by patient focus group to obtain satisfactory face validity

Researchers facilitated a patient focus group to assess the face validity of the 69 item questionnaire resulting from the expert review. Face validity is assessed by end users deciding whether the questionnaire appears to measure what the researchers who developed it claim.[33] A convenience sample of six individuals was recruited on March 4, 2014 from the County Durham and Darlington National Health Service Foundation Trust. Eligibility criteria was being aged 40-74 years and being free of known vascular disease. The focus group consisted of six white females between 50-64 years of age. The majority of participants had postgraduate education. These individuals worked as clerical workers, nurses and health improvement staff. They were not involved in the delivery of the NHS Health Check programme. Participants were asked to complete the 69 item questionnaire as well as to provide feedback on whether the items correctly measured the intended scales, appropriately stated the intent of the questionnaire, and matched the individual's situations.[32, 33] In addition, participants were asked to respond to questions about clarity, content, appropriateness, format, biases of questions and presentation of information. The resulting 65 item questionnaire is in Appendix B.

Phase II of Questionnaire Development

Modification of questionnaire to have satisfactory reliability

A 65 item questionnaire was administered to 110 NHS Health Check attendees immediately after their consultation between May 21 and July 28, 2014 in a cross sectional study in England. The aim

For peer review only - http://bmjopen.bmj.com/site/about/guidelines.xhtml

was to determine the content, the scale structure and the reliability of a questionnaire in its final form.

Study Recruitment

Eligibility criteria was completion of an NHS Health Check, being aged 40-74 years and free of known vascular disease. Of 110 study participants, 15 individuals were recruited by 2 nurses from a London general practice and 95 individuals by 13 community outreach providers from local community venues in Durham. These providers collected clinical risk factor data, informed study participants about their CVD risk, took informed study consent and distributed the 65 item questionnaire to be self-completed by NHS Health Check attendees following their consultation. Unlike general practice staff who operated only during business hours, community outreach providers worked on evenings and weekends as well as during regular business hours in community venues more accessible to the general public.

Data Analysis

To select appropriate items to constitute a scale, individual items were assessed during item analysis, item facility and item discrimination.[34] To determine the factorial structure of the questionnaire and which items together constituted particular scales, an Exploratory Factor Analysis (EFA) - a widely used technique in scale development was performed.[30, 35] The reliability of factors constituting particular scales was assessed using Cronbach's alpha coefficient. [36, 37] Reliability refers to consistency, reproducibility and agreement of a scale.[38]

In order to improve the quality of a scale and increase its reliability, individual items were assessed. Items with reverse scoring were re-coded to conform to the conceptual direction of the scales.[37] Each individual item was then examined for distortions in the pattern of responding known as skew and kurtosis.[33] Item facility examined whether items were answered in the same way by everyone by checking whether the facility index approached extreme scores or had a low standard deviation.[34] Items were assessed in terms of discriminating between participants' responses to the questionnaire's scales (Knowledge, Perceived CVD Risk, CVD Health Beliefs, Intentions / Readiness to Change and Self Efficacy). Discrimination was measured by item-total correlation with item correlating below 0.2 or any negative correlations resulting in deletion of items. In addition, discrimination was measured by the inter-item correlation within each scale resulting in deletion of items correlating with other items ≥0.60.[17, 34]

A Kaiser-Meyer-Olkin (KMO) measure of sampling adequacy and a Bartlett's test of sphericity were assessed to ensure that items were appropriate for EFA.[39] Next EFA was performed to define the scales of the questionnaire which share a similar underlying construct. Parallel Analysis was used to determine the optimum number of factors to be extracted using Principal Components Analysis (PCA) with a Varimax rotation. [34, 39, 40] PCA is a data reduction technique used to explain correlations among sets of items or variables as a few conceptually meaningful factors.[30] Compared to other available methods, Parallel Analysis using PCA was shown to be the best method of extracting factors and is appropriate when applied to data conforming to the formal factor analytic model.[39, 40] Iterations of EFA were carried out to identify core constituent items in each factor. Cross-loading items or items with loading <=0.50 were removed at each iteration.[39] Internal consistency reliability

For peer review only - http://bmjopen.bmj.com/site/about/guidelines.xhtml

of resulting factors was assessed using Cronbach's α coefficients with α>=0.70 indicating good reliability.[32,36, 37]

RESULTS

Construction of a draft questionnaire by review of relevant literature

We developed an 85 item questionnaire based on the theoretical framework, NHS guidelines and other validated questionnaires relating to heart disease.[17-19] The 85 item questionnaire had 8 subscales measuring *Knowledge of CVD Risk and Prevention* (18 items), *Perceived Risk and Vulnerability of CVD* (20 items), *Perceived Susceptibility* (5 items), *Perceived Severity* (5 items), *Perceived Benefits* (6 items), *Perceived Barriers* (7 items), *Self-Efficacy* (6 items), and *Intention to Change Behaviour* (18 items). *Knowledge of CVD Risk and Prevention* subscale items were measured using the following categories: *True, False*, and *Don't Know*. *Self-Efficacy* subscale items were measured using 5 point Likert scale ranging from *1=not at all confident* to *5=completely confident*. *Perceived Severity, Perceived Benefits, Perceived Barriers and Intention to Change Behaviour* subscale items were measured using a 4 point Likert scale ranging from *1=strongly disagree* to *4=strongly agree*. The reading level of the questionnaire was at Year 7.

Modification of questionnaire by expert panel to obtain satisfactory content validity

The expert panel concluded that out of the 85 items, 69 met the CVI>=0.80 criterion and were retained. In addition, the wording of a number of questions was revised to improve clarity. Diet and exercise were defined more precisely using frequency and duration. Response options of Self-Efficacy items were changed from a five point Likert scale to a four point Likert scale for consistency with the rest of the questionnaire. Questions pertaining to smoking and drinking were rephrased to apply to smokers and drinkers (see Table 1).

Table 1 Sample item wording modifications obtained through an expert panel

Original item(s)	Expert comments	Final item
The most important cause of heart attack and stroke is stress.	Revise to "one of the most important…" Substitute the word "important" with "main."	One of the main causes of heart attack and stroke is stress.
I have a high chance of getting a heart attack or stroke because of my past behaviours.	Add "and/or present behaviours."	I have a high chance of getting a heart attack or stroke because of my past and/or present behaviours.

For peer review only - http://bmjopen.bmj.com/site/about/guidelines.xhtml

Original item(s)	Expert comments	Final item
Increasing my exercise will decrease my chances of having a heart attack or stroke.	Define amount of exercise.	Increasing my exercise to at least 30 minutes a day will decrease my chances of having a heart attack or stroke.
Eating a healthy diet will decrease my chance of having a heart attack or stroke.	Define a healthy diet.	Eating at least five portions of fruit and vegetables a day will decrease my chances of having a heart attack or stroke.
When I exercise I am doing something good for myself.	Define exercise consistently. Make the statement more specific about the heart.	When I exercise for 30 minutes a day I am doing something good for the health of my heart.
How confident are you that you know or can…? questions answered using a 5-point Likert scale: “not at all confident, somewhat confident, moderately confident, very confident, completely confident.”	Use a 4-point Likert to maintain consistency.	Five point Likert scale changed to a 4 point Likert scale: “not at all confident, somewhat confident, very confident, completely confident.”
How confident are you that you know how or can stop smoking if you want to?	Instead of saying “…that you know or can” say “that you know how to or can…” Add in parentheses “if you smoke.”	How confident are you that you know how to or can stop smoking if you want to (if you smoke)?
I want to cut down on alcohol. I intend to cut down on alcohol in the next two months.	Conceptual overlap between want to and intend to. Add in parentheses “if you drink alcohol.”	I intend or want to cut down on alcohol (if you drink alcohol).

Modification of questionnaire by patient focus group to obtain satisfactory face validity

As a result of the focus group review of the 69 item questionnaire, six items were removed, two items were added and a number of items were modified leaving a final total of 65 items with satisfactory face validity. A not applicable category was added to 50 items while the response categories to Knowledge subscale items remained unchanged. Exercise was redefined in 8 items

For peer review only - http://bmjopen.bmj.com/site/about/guidelines.xhtml

from 150 minutes a week and 30 minutes a day to 2.5 hours a week. A negatively framed question was reframed positively (see Table 2).

Table 2 Sample item wording modifications and additions through the patient focus group

Original item	Participant comments	Final item
Moderate physical activity of 150 minutes a week will reduce your chances of developing a heart or stroke.	2.5 hours a week is better than 150 minutes.	Moderate physical activity of 2.5 hours a week will reduce your chances of developing a heart or stroke.
Drinking alcohol has nothing to do with reducing the risk of heart attack or stroke.	Question is negatively stated.	Drinking high levels of alcohol can increase your cholesterol and triglyceride levels.
Missing question	Need to include family history of disease to account for genetic predisposition.	A family history of hypertension is not a risk factor for high blood pressure.
Missing question	Benefits of not smoking?	If I stopped smoking it will reduce my chances of having a heart attack or stroke.
Increasing my exercise for 30 minutes a day will decrease my chances of having a heart attack or stroke.	Two and a half hours a week is better than 30 minutes a day.	Increasing my exercise to at least 2 ½ hours a week will decrease my chances of having a heart attack or stroke.
I have reduced or stopped smoking (if you smoke).<br>“strongly disagree, disagree, agree, and strongly agree.”	Remove (if you smoke).<br>Add a “not applicable” box.	I have reduced or stopped smoking.<br><br>“strongly disagree, disagree, agree, and strongly agree, not applicable.”
How confident are you that you know how to or can consume recommended levels of alcohol (if you drink alcohol)?<br><br>“not at all confident, somewhat confident, very confident and completely	Remove (if you drink alcohol).<br>Add a “not applicable” box.	How confident are you that you know how to or can drink within the recommended levels of alcohol?<br><br>“not at all confident, somewhat confident, very confident and completely

For peer review only - http://bmjopen.bmj.com/site/about/guidelines.xhtml

Original item	Participant comments	Final item
confident."		confident, not applicable."

Modification of questionnaire to have satisfactory reliability

The 65 item questionnaire that resulted from content and face validity assessments, was administered to 110 NHS Health Check attendees immediately after their NHS Health Check consultation. The majority of study participants were White (84.5%), younger than 60 (77.3%) and had at least one or more CVD risk factors. Using the Index of Multiple Deprivation, a relative measure of deprivation across seven distinct domains including income, employment, health and disability, education skills and training, barriers to housing and services, living environment and crime, [41] people in the two most deprived fifths were 40.0% of the study population. See Appendix C for study population characteristics. The responses to the questionnaire were analysed as individual items during item analysis, item facility and item discrimination. In addition, the scale structure and reliability of resulting scales were assessed.

No items were removed during item analysis and item facility. During item discrimination assessment using item-total correlation, seven items in the Knowledge scale, four items in Perceived CVD Risk, three items in CVD Health Benefits, three items in Intention and or Readiness to Change were deleted due to item-total correlations falling below 0.2. [33] During item discrimination assessment using inter-item correlation, two items in Perceived CVD Risk and three items in Intentions / Readiness to Change were removed as these items correlated greater than 0.6 with other items.[33] Although there were two items that correlated above 0.6 in CVD Risk Reduction Self Efficacy, these remained in the questionnaire as the items were qualitatively different: *Stop smoking if you want to* and *Control the risks of having a heart attack or stroke.* In total, 22 items were removed during item discrimination analysis, leaving 43 items which had good item facility and discrimination.

Of the 43 remaining items, 8 items of the "Knowledge" scale with *"true"* or *"false"* scoring could not entered into EFA. Of the 35 items scored on a four point Likert scale, four items pertaining to smoking were deleted as they had a high proportion of missing responses (69-80%). The resulting 31 items had a Kaiser-Meyer-Olkin (KMO) measure of sampling adequacy of 0.32 and a significant Bartlett's test of sphericity (1020.50, p <.001), indicating that these data were appropriate for EFA.[39] After 12 iterations of EFA, 20 items loaded above 0.50 on the factors and there were no cross-loadings indicating good factor structure (see Table 3). Internal consistency reliability of factor structure was measured using Cronbach's α. Factor 1 (8 items): (Perceived Risk of Heart Attack/Stroke) had α =.85. Factor 2 (7 items): (Perceived Benefits & Intentions to Change) had α =.82. Factor 3 (3 items): (Healthy Eating Intentions) had α =.56. Factor 4 (2 items): (Intentions towards Alcohol) had α =-0.16. Although Healthy Eating Intentions α = 0.56 is below the minimum threshold (0.70) for reliability, this is acceptable for a three item scale.[34] The intention toward alcohol factor had two items with such low reliability (α = -0.16) that they could not be considered a separate factor and were removed. A thirteenth EFA iteration confirmed the factor loadings and reliabilities reported above. Hence the parallel analysis indicated that three factors should be retained. [39] The three factor model accounted for 57.61% of the total explained variance.

For peer review only - http://bmjopen.bmj.com/site/about/guidelines.xhtml

Table 3 Factor structure of the ABCD Risk Questionnaire

	Components		
	Factor 1 Perceived Risk of Heart Attack / Stroke	Factor 2 Perceived Benefits & Intentions to Change	Factor 3 Healthy Eating Intentions
It is likely that I will suffer from a heart attack or stroke in the future.	.844		
It is likely that I will have a heart attack or stroke some time during my life.	.816		
I feel I will suffer from a heart attack or stroke sometime during my life.	.809		
There is a good chance I will experience a heart attack or stroke in the next 10 years.	.752		
I am not worried that I might have a heart attack or stroke.	.705		
My chances of suffering from a heart attack or stroke in the next 10 years are great.	.687		
It is likely I will have a heart attack or stroke because of my past and/or present behaviours.	.639		
I am concerned about the likelihood of having a heart attack or stroke in the near future.	.575		
I am thinking about exercising at least 2 ½ hours a week.		.826	
I intend or want to exercise at least 2 ½ hours a week.		.792	
When I exercise for at least 2½ hours a week I am doing something good for the health of my heart.		.735	

For peer review only - http://bmjopen.bmj.com/site/about/guidelines.xhtml

	Components		
	Factor 1 Perceived Risk of Heart Attack / Stroke	Factor 2 Perceived Benefits & Intentions to Change	Factor 3 Healthy Eating Intentions
I am confident that I can maintain a healthy weight by exercising at least 2½ hours a week within the next two months.		.658	
I am not thinking about exercising for 2 ½ hours a week.		.656	
When I eat at least five portions of fruit and vegetables a day I am doing something good for the health of my heart.		.642	
Increasing my exercise to at least 2½ hours a week will decrease my chances of having a heart attack or stroke.		.557	
I am confident that I can eat at least five portions of fruit and vegetables per day within the next two months.			.830
I am thinking about eating at least five portions of fruit and vegetables a day.			.772
I am not thinking about eating at least five portions of fruit and vegetables a day.			.731

Note: Factor loadings and commonalities are reported following an EFA using Principal Component Analysis with Varimax rotation.

The EFA revealed three scales: Perceived Risk of Heart Attack / Stroke, Perceived Benefits and Intentions to Change and Healthy Eating Intentions. A fourth scale assessing Knowledge of CVD Risk and Prevention (not entered into EFA) was added back to the questionnaire following EFA (see Figure 2). Hence the final questionnaire included 26 items grouped into four scales: Knowledge of CVD Risk and Prevention (8 items), Perceived Risk of Heart Attack/Stroke (7 items), Perceived Benefits and Intention to Change Behaviour (7 items) and Healthy Eating Intentions (3 items). In the resulting 26 item questionnaire, two items were changed from questions “How confident are you that you know how to or can…” to statements of agreement “I am confident that I can” so as to be

answered using the same Likert scale. The ABCD Risk Questionnaire with a scoring guide for each scale is reported in Appendix D.

DISCUSSION

To the best of our knowledge this is the first study that describes the development of a short, validated questionnaire examining CVD risk awareness among the NHS Health Check attendees. Satisfactory content and face validity as well as reliability of the ABCD Risk Questionnaire suggest that the tool performs well. It may be used for evaluating the accuracy of perceived CVD risk, general knowledge of CVD and intention to change behaviour in regards to diet and exercise among NHS Health Check attendees. As the ABCD Risk Questionnaire was developed using both an expert panel and a patient focus group, the questions ought to be relatively easy to understand for both patients and clinicians. If NHS Health Check recommendations change over time, the ABCD Risk Questionnaire may need to be updated.

Critics of the NHS Health Check programme point to the lack of its evidence base. [42, 43] The majority of evaluations focused on coverage and uptake, statin prescribing, new diagnoses and CVD risk factor reduction. [44-49] As there was no instrument measuring CVD risk awareness, no studies examined the patients' understanding of CVD risk among NHS Health Check attendees. CVD risk presentation was shown to increase the accuracy of perceived risk by 10%. When risk information is repeated this leads to small but significant reductions in predicted CVD risk.[16] A national study showed modest reductions in 10 year predicted CVD risk among NHS Health Check attendees in the first four years.[48] A limitation of using predicted ten year risk of CVD is the under-estimation of CVD risk among women and younger people. [35] More research is needed to establish whether the programme improves NHS Health Check attendees' awareness of CVD risk and whether the programme has an impact on predicted lifetime CVD risk.

The ABCD Risk Questionnaire was developed and tested on a non-risk stratified population as the NHS Health Check programme is administered to all eligible people free of vascular disease diagnosis irrespective of their level of CVD risk. As such it does not encompass all aspects of CVD risk observed in the general population. Questions on smoking and drinking were progressively eliminated as they did not apply to the majority of the study participants. As questions on diet and exercise pertained to all people regardless of their level of CVD risk, such questions that reliably distinguished between study participants were selected for inclusion. Although fruit and vegetable intake is only one aspect of diet in the EatWell Guide recommended for use in NHS Health Check,[50] it is the only assessment of diet recorded during the NHS Health Check. The final questionnaire contains questions based on data collected during NHS Health Check to enable subsequent programme evaluation.[51]

Judging by the number of items reduced in various stages of development, the final questionnaire was largely shaped by analysis of data from 110 NHS Health Check attendees completing the 65 item questionnaire. This study population was representative of the population that took up the NHS Health Check programme between 2009-2014 in terms of socio-demographics including the proportion of men (46.4%), ethnic minorities (5.4%), individuals from the most deprived two fifths (40.9%), and clinical risk factors including mean total cholesterol (5.42 (95% CI 5.19, 5.64)), BMI (27.24 95% CI 26.17, 28.31), smokers (18.2%) and those at high CVD risk (4.5%).[44] As higher levels of deprivation are partly due to having less education,[41] questionnaire development was not limited to

For peer review only - http://bmjopen.bmj.com/site/about/guidelines.xhtml

people with higher education. Compared to the national evaluation, similar levels of high CVD risk were observed despite the fact that the study population contained more younger people aged 40-59 (77.3%).[44] The recruitment of hard to reach groups including younger people, socio-economically deprived individuals and ethnic minorities by community outreach providers in community venues outside of conventional working hours is consistent with prior literature. [22, 52, 53, 54]

A possible limitation to face validity is that the patient focus group evaluating the 69 item questionnaire was not representative of the target population. Whereas the NHS Health Check programme is administered to both men and women and members of ethnic minorities, the focus group consisted only of white women. Furthermore as these women had postgraduate education and worked in a health related field, they may have had higher health literacy than the general population eligible for the NHS Health Check programme. Clarity, appropriateness, biases and presentation of information may have been differentially assessed by people with different levels of health literacy. A community based recruitment method aiming to recruit some of the hard to reach groups may have been more effective in getting a more representative patient focus group.

Additional studies should be conducted with larger samples to confirm the reliability and validity of the questionnaire. It would be useful to replicate the factor analytic process on an independent, larger sample to confirm the generalizability of these findings.[373737]

CONCLUSIONS

The ABCD Risk Questionnaire showed evidence of satisfactory reliability and validity, is brief and easy to use. By capturing patients' views on CVD risk awareness during an NHS Health Check consultation, the questionnaire can be used to assess patients' understanding of CVD risk. Clinicians administering the questionnaire to patients may help to establish whether the programme is effective in empowering patients to make informed lifestyle choices about their health.

References

1. World Health Organisation. Prevention of Cardiovascular Disease - Guidelines for assessment and management of cardiovascular risk, 2007. http://www.who.int/cardiovascular_diseases/guidelines/Full%20text.pdf (accessed 08 March 2015)
2. Murray CJL, Richards MA, Newton JN, et al. UK health performance: findings of the Global Burden of Disease Study 2010. *The Lancet*. 2013; 381: 997-1020.
3. Barton GR, Goodall M, Bower P, Woolf S, Capewell S and Gabbay MB. Increasing heart-health lifestyles in deprived communities: economic evaluation of lay health trainers. *Journal of Evaluation in Clinical Practice*. 2012; 18: 835-40.
4. Yusuf S, Hawken S, Ôunpuu S, et al. Effect of potentially modifiable risk factors associated with myocardial infarction in 52 countries (the INTERHEART study): case-control study. *The Lancet*. 2004; 364: 937-52.
5. Bajekal M, Scholes S, Love H, et al. Analysing Recent Socioeconomic Trends in Coronary Heart Disease Mortality in England, 2000–2007: A Population Modelling Study. *PLoS Med*. 2012; 9: e1001237.
6. The National Institute for Health and Care Excellence (NICE). Services for the prevention of cardiovascular disease - NICE commissioning guides [CMG45], 2012.

For peer review only - http://bmjopen.bmj.com/site/about/guidelines.xhtml

ttps://www.nice.org.uk/guidance/cmg45/chapter/commissioning-services-for-the-prevention-of-cardiovascular-disease (accessed 12 June 2015)
7. Department of Health. Putting prevention first- vascular checks: risk assessment and management - next steps guidance for primary care trusts, 2009. http://www.dh.gov.uk/en/Publicationsandstatistics/Publications/PublicationsPolicyAndGuidance/DH_090277 (accessed 7 July 2016).
8. Public Health England. NHS Health Check Best Practice Guidance 2015, 2015. http://www.healthcheck.nhs.uk/commissioners_and_healthcare_professionals/national_guidance/ (accessed 6 March 2015)
9. Public Health England. NHS Health Check: Our Approach to the Evidence, 2013. https://www.gov.uk/government/uploads/system/uploads/attachment_data/file/224537/NHS_Health_Check_our_approach_to_the_evidence_v2.pdf;(accessed 12 June 2015).
10. Department of Health. Vascular Checks: Economic Modelling for Vascular Checks, 2008. http://www.dh.gov.uk/en/Publicationsandstatistics/Publications/PublicationsPolicyAndGuidance/DH_085869 (accessed 12 June 2015).
11. Homko CJ, Santamore WP, Zamora L, et al. Cardiovascular Disease Knowledge and Risk Perception Among Underserved Individuals at Increased Risk of Cardiovascular Disease. *Journal of Cardiovascular Nursing*. 2008; 23: 332-7
12. Collins GS and Altman DG. Predicting the 10 year risk of cardiovascular disease in the United Kingdom: independent and external validation of an updated version of QRISK2. *BMJ*. 2012; 344.
13. Webster R and Heeley E. Perceptions of risk: understanding cardiovascular disease. *Risk management and healthcare policy*. 2010; 3: 49-60.
14. Kirkland SA, MacLean DR, Langille DB, Joffres MR, MacPherson KM and Andreou P. Knowledge and awareness of risk factors for cardiovascular disease among Canadians 55 to 74 years of age: results from the Canadian Heart Health Surveys, 1986-1992. *CMAJ : Canadian Medical Association journal = journal de l'Association medicale canadienne*. 1999; 161: S10-6.
15. Potvin L, Richard L and Edwards AC. Knowledge of cardiovascular disease risk factors among the Canadian population: relationships with indicators of socioeconomic status. *CMAJ : Canadian Medical Association journal = journal de l'Association medicale canadienne*. 2000; 162: S5-11.
16. Sheridan SL, Viera AJ, Krantz MJ, et al. The Effect of Giving Global Coronary Risk Information to Adults: A Systematic Review. *Archives of Internal Medicine*. 2010; 170: 230-9.
17. Bergman HE, Reeve BB, Moser RP, Scholl S and Klein WMP. Development of a Comprehensive Heart Disease Knowledge Questionnaire. *American Journal of Health Education*. 2011; 42: 74-87.
18. Ammouri AA and Neuberger G. The Perception of Risk of Heart Disease Scale: development and psychometric analysis. *J Nurs Meas*. 2008; 16: 83-97.
19. Tovar EG, Rayens MK, Clark M and Nguyen H. Development and psychometric testing of the Health Beliefs Related to Cardiovascular Disease Scale: preliminary findings. *Journal of advanced nursing*. 2010; 66: 2772-84.
20. Strecher V.J. R, I.M. *The Health Belief Model. In Health Behavior and Health Education, Theory, Research and Practice (Glanz, K., Lewis, F.M., Rimer, B.K. eds)*. San Francisco: Jossey-Bass, 1997.
21. Prochaska JO, Redding, C.A., Evers, K.E. . *The Transtheoretical Model and Stages of Change. In Health Behavior and Health Education, Theory, Research and Practice (Glanz, K., Lewis, F.M., Rimer, B.K. eds).* . San Francisco: Jossey-Bass, 1997.
22. Hsu HY and Gallinagh R. The relationships between health beliefs and utilization of free health examinations in older people living in a community setting in Taiwan. *Journal of advanced nursing*. 2001; 35: 864-73.
23. Prochaska JO, DiClemente CC and Norcross JC. In search of how people change. Applications to addictive behaviors. *Am Psychol*. 1992; 47: 1102-14.

For peer review only - http://bmjopen.bmj.com/site/about/guidelines.xhtml

24. Prochaska JO, Norcross JC and DiClemente CC. *Changing for Good: A Revolutionary Six-Stage Program for Overcoming Bad Habits and Moving Your Life Positively Forward.* New York, NY Avon Books, 1994.
25. Department of Health. Physical Activity Guidelines for Adults (19-64 Years), 2011. http://www.nhs.uk/Livewell/fitness/Documents/adults-19-64-years.pdf (accessed 23 April 2015).
26. Department of Health. Improve Your NHS Health Check Numbers, 2014. http://www.nhs.uk/Conditions/nhs-health-check/Pages/Take-action-to-improve-your-health.aspx (accessed 23 April 2015).
27. Department of Health. Five a Day, 2013. http://www.nhs.uk/livewell/5aday/Pages/ADAYhome.aspx (accessed 23 April, 2015).
28. Health Do. Putting prevention first. NHS health check: Vascular risk assessment and management - best practice guidance. 2009.
29. Andrew S and Halcomb EJ. *Mixed Methods Research for Nursing and the Health Sciences*. Blackwell Publishing Ltd, 2009.
30. Chan CW. Perceptions of coronary heart disease: the development and psychometric testing of a measurement scale. *Psychol Health Med*. 2014; 19: 159-68.
31. Wells KJ, Arevalo M, Meade CD, et al. Development and validation of the biobanking attitudes and knowledge survey (BANKS). *Cancer Epidemiol Biomarkers Prev*. 2014; 23: 374-82.
32. Waltz CF, Strickland OL and Lenz ER. *Measurement in Nursing Research*. Second Edition ed. Philadelphia: F.A. Davis Company, 1991.
33. Waltz CF, Strickland OL and Lenz ER. *Measurement in Nursing and Health Research* Fourth Edition ed. New York: Springer Publishing Company, 2010.
34. Rust J and Golombok S. *Modern psychometrics: The science of psychological assessment.* Second Edition ed. London: Routledge, 1999.
35. JBS 3 Joint British Societies for the Prevention of Cardiovascular Disease. Lifetime Risk, 2014. http://www.jbs3risk.com/pages/lifetime_risk.htm (accessed 9 August 2016).
36. Nunnally JC and Bernstein IH. *Psychometric theory*. Third Edition ed. New York: McGraw-Hill Higher, INC, 1994.
37. DeVellis RF. *Scale Development Theory and Applications*. Third Edition ed. Los Angelos / London / New Delhi / Singapore / Washington DC: SAGE, 2012.
38. Aldridge V, Wade A and Koutoumanou E. Validity and Reliability University College London, Institute of Child Health2015.
39. Ferguson E and Cox T. Exploratory Factor Analysis: A Users'Guide. *International Journal of Selection and Assessment*. 1993; 1: 84-94.
40. Zwick WR and Velicer WF. Comparison of five rules for determining the number of components to retain. *Psychological Bulletin*. 1986; 99: 432-42.
41. Department for Communities and Local Government. The English Indices of Deprivation 2010 - Neighborhoods Statistical Release, 2011. https://www.gov.uk/government/uploads/system/uploads/attachment_data/file/6871/1871208.pdf (accessed 16 December 2014).
42. Krogsbøll Lasse T, Jørgensen Karsten J, Grønhøj Larsen C and Gøtzsche Peter C. General health checks in adults for reducing morbidity and mortality from disease. *Cochrane Database of Systematic Reviews*: (2012).
43. Capewell S and Graham H. Will cardiovascular disease prevention widen health inequalities? *PLoS Med*. 2010; 7: e1000320.
44. Chang KC-M, Soljak M, Lee JT, et al. Coverage of a national cardiovascular risk assessment and management programme (NHS Health Check): Retrospective database study. *Preventive Medicine*.
45. Artac M, Dalton ARH, Majeed A, Car J and Millett C. Effectiveness of a national cardiovascular disease risk assessment program (NHS Health Check): Results after one year. *Preventive Medicine*. 2013.

For peer review only - http://bmjopen.bmj.com/site/about/guidelines.xhtml

46. Artac M, Dalton ARH, Majeed A, Car J, Huckvale K and Millett C. Uptake of the NHS Health Check programme in an urban setting. *Family Practice*. 2013; 30: 426-35.
47. Dalton ARH, Bottle A, Okoro C, Majeed A and Millett C. Uptake of the NHS Health Checks programme in a deprived, culturally diverse setting: cross-sectional study. *J Public Health (Oxf)*. 2011; 33: 422-9.
48. Chang KC, Lee JT, Vamos EP, et al. Impact of the National Health Service Health Check on cardiovascular disease risk: a difference-in-differences matching analysis. *CMAJ : Canadian Medical Association journal = journal de l'Association medicale canadienne*. 2016.
49. Robson J, Dostal I, Sheikh A, et al. The NHS Health Check in England: an evaluation of the first 4 years. *BMJ Open*. 2016; 6: e008840.
50. NHS Choices. Eating a Balanced Diet. 2016. http://www.nhs.uk/Livewell/Goodfood/Pages/Healthyeating.aspx (accessed 27 April 2017).
51. Public Health England. NHS Health Check Best practice guidance. 2017. http://www.healthcheck.nhs.uk/commissioners_and_providers/guidance/national_guidance1/ (accessed 27 April 2017).
52. Grayland J and Wilson R. Improving male life expectancy in Birmingham. *Public Health*. 2009; 123: e50-e6.
53. Hunt BD, Hiles SL, Chauhan A, et al. Evaluation of the Healthy LifeCheck programme: a vascular risk assessment service for community pharmacies in Leicester city, UK. *J Public Health (Oxf)*. 2013; 35: 440-6.
54. Visram S, Carr SM and Geddes L. Can lay health trainers increase uptake of NHS health checks in hard-to-reach populations? A mixed-method pilot evaluation. *J Public Health (Oxf)*. 2014.

For peer review only

For peer review only - http://bmjopen.bmj.com/site/about/guidelines.xhtml

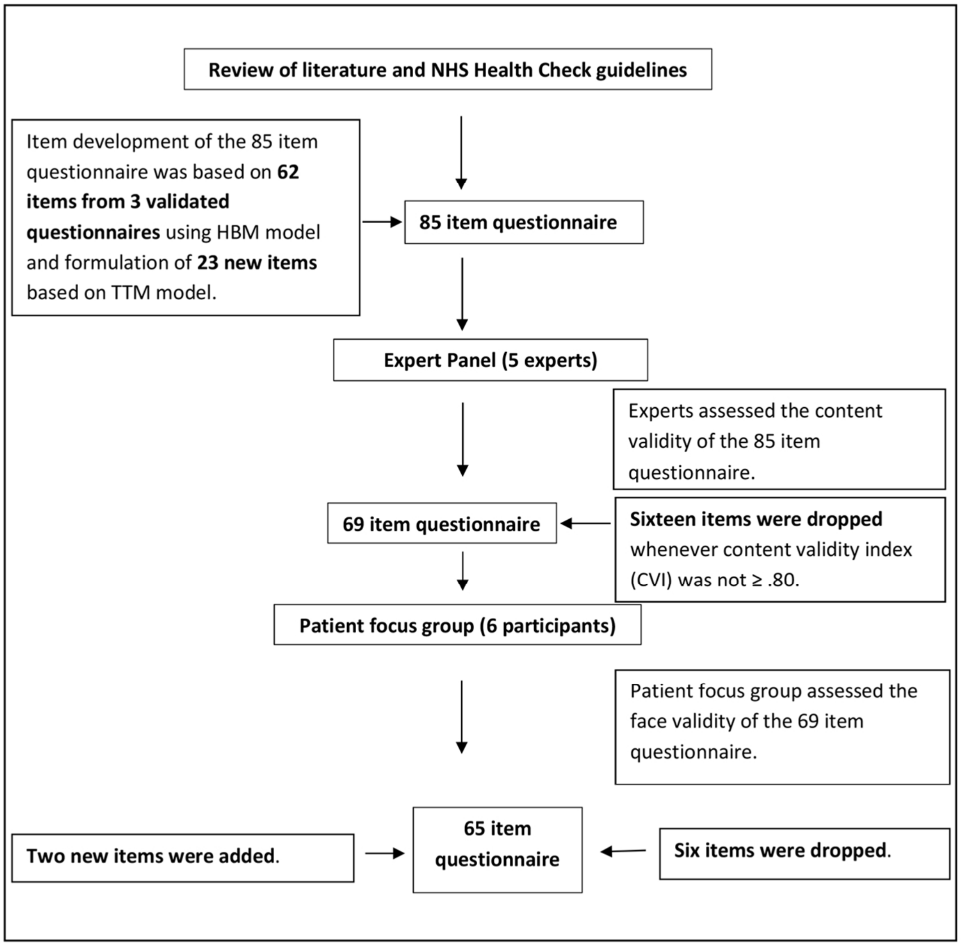

Figure 1

112x112mm (300 x 300 DPI)

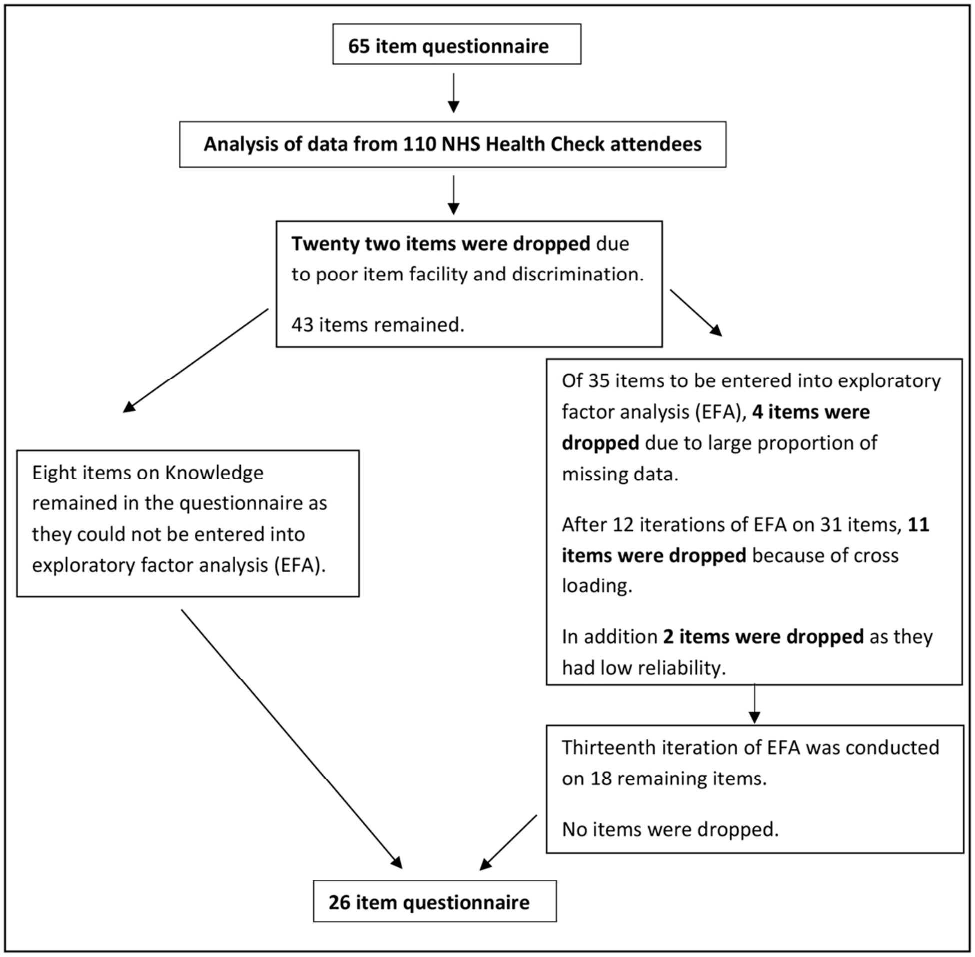

Figure 2

112x112mm (300 x 300 DPI)

Appendix A: 85 Item Questionnaire

Subscale	Items	Answers
Knowledge of CVD Risk and Prevention	1. Eating a lot of red meat increases heart attack and stroke risk.	True, False, Don't Know<br>T=True F=False<br>Correct Answers<br>Q1=T Q2=F Q3=T Q4=F Q5=T Q6=F Q7=F Q8=T Q9=T Q10=T Q11=F Q12=T Q13=T Q14=T Q15=T Q16=F Q17=T Q18=F
	2. Most people can tell whether or not they have high blood pressure.	
	3. You can reduce your risk of heart attack or stroke by being physically active.	
	4. 'High' blood pressure is defined as 110/80 (systolic/diastolic) or higher.	
	5. Dietary fibre lowers blood cholesterol.	
	6. The most important cause of heart attack and stroke is stress.	
	7. Trans-fats are healthier for the heart than most other kinds of fats.	
	8. Walking and gardening are considered types of exercise that can lower the risk of having a heart attack or stroke.	
	9. You can reduce your chance of developing a heart attack or stroke by eating five-a-day diet of fruits and vegetables.	
	10. Moderate physical activity of 150 minutes a week will reduce your chances of developing a heart attack or stroke.	
	11. People who quit smoking by 60 add five years to their life.	
	12. People who have diabetes are at higher risk having a heart attack or stroke.	
	13. Managing your stress levels will help you to manage your blood pressure.	
	14. HDL refers to 'good' cholesterol, and LDL refers to 'bad' cholesterol.	
	15. The healthiest exercise for the heart involves rapid breathing for a sustained period of time.	
	16. Many vegetables are high in cholesterol.	
	17. You are more likely to have a heart attack or stroke if you're overweight or obese.	
	18. Drinking alcohol has nothing to do with reducing the risk of heart attack or stroke.	
Perceived Risk and Vulnerability of CVD	19. There is a possibility that I will have a heart attack or stroke.	1 = Strongly disagree; 2 = disagree; 3 = agree; 4 = strongly agree
	20. There is a good chance I will experience a heart attack or stroke during the next 10 years.	
	21. A person who gets a heart attack or stroke has no chance of recovering.	
	22. I have a high chance of getting a heart attack or stroke because of my past behaviours.	
	23. I feel sure that I will have a heart attack or stroke.	
	24. Healthy lifestyle habits are unattainable.	
	25. It is likely that I will get a heart attack or stroke.	
	26. I am at risk for having a heart attack or stroke.	
	27. It is possible that I will have a heart attack or stroke.	
	28. I am not doing anything now that is unhealthy to my heart.	
	29. I am too young to have a heart attack or stroke.	
	30. People like me do not get a heart attack or stroke.	
	31. I am very healthy so I will not have a heart attack or stroke.	
	32. I am not worried that I might have a heart attack or stroke.	
	33. People my age are too young to have a heart attack or stroke.	

For peer review only

	34. People my age do not have a heart attack or stroke.	
	35. My lifestyle habits do not put me at risk for having a heart attack or stroke.	
	36. No matter what I do, if I am going to have a heart attack or stroke, I will have one.	
	37. People who do not have a heart attack or stroke are just plain lucky.	
	38. The causes of a heart attack or stroke are unknown.	
Perceived Susceptibility	39. It is likely that I will suffer from a heart attack or stroke in the future.	
	40. My chances of suffering from a heart attack or stroke in the next few years are great.	
	41. Having a heart attack or stroke is currently a possibility for me.	
	42. I feel I will suffer from a heart attack or stroke sometime during my life.	
	43. I am concerned about the likelihood of having a heart attack or stroke in the near future.	
Perceived Severity	44. Heart attacks and strokes are always fatal.	
	45. Having a heart attack or stroke will threaten my relationship with my significant other.	
	46. My whole life would change if I had a heart attack or stroke.	
	47. Having a heart attack or stroke would have a very bad effect on my sex life.	
	48. If I have a heart attack or stroke I will die within 10 years.	
Perceived Benefits	49. Increasing my exercise will decrease my chances of having a heart attack or stroke.	
	50. Eating a healthy diet will decrease my chance of having a heart attack or stroke.	
	51. Stopping smoking will reduce my chance of having a heart attack or stroke.	
	52. When I exercise I am doing something good for myself.	
	53. When I eat healthy I am doing something good for myself.	
	54. Cutting down on alcohol will decrease my chances of having a heart attack or stroke.	
Perceived Barriers	55. I do not know appropriate exercises to perform to reduce my risk of developing cardiovascular disease.	
	56. I do not know the recommended drinking limits for men or women.	
	57. I do not have time to exercise for 30 minutes a day on most days of the week.	
	58. I do not know what is considered a healthy diet that would prevent me from developing cardiovascular disease.	
	59. I will not have energy if I stop smoking.	
	60. I cannot afford to buy healthy foods.	
	61. I have other problems more important than worrying about diet and exercise.	
Self Efficacy	62. How confident are you that you know or can control the risks of having a heart attack or stroke?	1= not at all confident, 2=somewhat confident, 3= moderately confident, 4=very confident,
	63. How confident are you that you know or can maintain a healthy weight by exercising regularly?	
	64. How confident are you that you know or can stop smoking if you want to?	
	65. How confident are you that you know or can consume less alcohol?	

For peer review only - http://bmjopen.bmj.com/site/about/guidelines.xhtml

	66. How confident are you that you know or can control your blood pressure and/or cholesterol levels by taking your prescribed medications?	5=completely confident
	67. How confident are you that you know or can eat a healthy and balanced diet?	
Intention to Change Behaviour or Cues to Action	68. I want to stop smoking (if you do smoke).	1 = Strongly disagree; 2 = disagree; 3 = agree; 4 = strongly agree
	69. I intend to maintain a healthy weight.	
	70. I intend to be physically active within two months.	
	71. I expect to maintain a healthy weight.	
	72. I want to be physically active.	
	73. I intend to eat a healthy and balanced diet within two months.	
	74. I expect to stop smoking (if you do smoke).	
	75. I want to cut down on alcohol.	
	76. I want to maintain a healthy and balanced diet.	
	77. I intend to stop smoking within two months (if you do smoke).	
	78. I expect to eat a healthy and balanced diet.	
	79. I intend to cut down on alcohol in the next two months.	
	80. I expect to be physically active.	
	81. I expect to cut down on alcohol.	
	82. I want to eat a healthy and balanced diet.	
	83. I expect to take my medication to control my blood pressure and/or cholesterol	
	84. I want to take my medication to control my blood pressure and/or cholesterol	
	85. I intend to take my medication to control my blood pressure and/or cholesterol within two months	

Appendix B: 65 Item Questionnaire

For peer review only - http://bmjopen.bmj.com/site/about/guidelines.xhtml

Scale	Subscale	Items	Coding of Answers
**Knowledge**<br><br>(15 items)<br><br>Higher sum score = more knowledge able / more correct	**CVD Risk Knowledge – Risk of having a heart attack / stroke**<br><br>(15 items)	1. Eating a lot of red meat increases heart attack and stroke risk.	Correct Answers<br><br>Q1-T Q6-T Q11-F<br>Q2-F Q7-T Q12-T<br>Q3-T Q8-T Q13-T<br>Q4-T Q9-T Q14-T<br>Q5-T Q10-T Q15-F<br><br>T= True<br>F= False<br><br>Correct: Score = 1, Incorrect or Don't Know Score = 0.
		2. Most people can tell whether or not they have high blood pressure.	
		3. You can reduce your risk of heart attack or stroke by being physically active.	
		4. One of the main causes of heart attack and stroke is stress.	
		5. Walking and gardening are considered types of exercise that can lower the risk of having a heart attack or stroke.	
		6. You can reduce your chance of developing a heart attack or stroke by eating at least five portions of fruit and vegetables a day.	
		7. Moderate intensity activity of 2 ½ hours a week will reduce your chances of developing a heart attack or stroke.	
		8. People who have diabetes are at higher risk of having a heart attack or stroke.	
		9. Managing your stress levels will help you to manage your blood pressure.	
		10. The healthiest exercise for the heart involves rapid breathing for 10 minutes or more.	
		11. Many vegetables are high in cholesterol.	
		12. You are more likely to have a heart attack or stroke if you're overweight or obese.	
		13. Drinking high levels of alcohol can increase your cholesterol and triglyceride levels.	
		14. HDL refers to 'good' cholesterol, and LDL refers to 'bad' cholesterol.	
		15. Family history of heart disease is not a risk factor for high blood pressure.	

For peer review only - http://bmjopen.bmj.com/site/about/guidelines.xhtml

**Perceived CVD Risk** (15 items) Composite score = sum across subscales. Higher score = higher perception of risk of having a heart attack or stroke	**Dread Risk** (7 items)	16. There is a possibility that I will have a heart attack or stroke.	Higher sum score = Higher perceived lack of control, dread, catastrophic potential and fatal consequences<br><br>1 = Strongly disagree; 2 = disagree; 3 = agree; 4 = strongly agree; N/A = 0
		17. There is a good chance I will experience a heart attack or stroke in the next 10 years.	
		18. It is likely I will have a heart attack or stroke because of my past and/or present behaviours.	
		19. I feel sure that I will have a heart attack or stroke.	
		20. It is likely that I will have a heart attack or stroke some time during my life.	
		21. I am at risk for having a heart attack or stroke some time during my life.	
		22. It is possible that I will have a heart attack or stroke within the next 10 years.	
	**Risk** (3 items)	23. I am too young to have a heart attack or stroke.	Higher sum score = Higher perceived hazards that has few, moderate, known outcomes and consequences<br><br>**Reverse coded**<br>4 = Strongly disagree; 3 = disagree; 2 = agree; 1 = strongly agree; N/A = 0
		24. People like me do not have a heart attack or stroke.	
		25. I am not worried that I might have a heart attack or stroke.	
	**Unknown Risk** (5 items)	26. I am not doing anything now that is unhealthy to my heart.	Higher sum score = Higher perceived hazards judged to be unobservable, unknown, new, and delayed in their manifestation of harm<br><br>**Reverse coded**<br>4 = Strongly disagree; 3 = disagree; 2 = agree; 1 = strongly agree; N/A = 0
		27. I am very healthy so I will not have a heart attack or stroke.	
		28. My lifestyle habits do not put me at risk for having a heart attack or stroke.	
		29. No matter what I do, if I am going to have a heart attack or stroke, I will have one.	
		30. People who do not have a heart attack or stroke are just plain lucky.	

For peer review only - http://bmjopen.bmj.com/site/about/guidelines.xhtml

**CVD Health** **Beliefs** (13 items)	**Susceptibility** (4 items)	31. It is likely that I will suffer from a heart attack or stroke in the future.	Higher average score = Higher perceived personal risk of heart attack and stroke<br>1 = Strongly disagree; 2 = disagree; 3 = agree; 4 = strongly agree; N/A = 0
		32. My chances of suffering from a heart attack or stroke in the next 10 years are great.	
		33. I feel I will suffer from a heart attack or stroke sometime during my life.	
		34. I am concerned about the likelihood of having a heart attack or stroke in the near future.	
	**Severity** (3 items)	35. Heart attacks and strokes are always fatal.	Higher average score = Higher perceived severity of heart attack and stroke<br>1 = Strongly disagree; 2 = disagree; 3 = agree; 4 = strongly agree; N/A = 0
		36. My whole life would change if I had a heart attack or stroke.	
		37. If I have a heart attack or stroke I will die within 10 years.	
	**Benefits** (4 items)	38. Eating at least five portions of fruit and vegetables a day will decrease my chances of having a heart attack or stroke.	Higher average score = Higher perceived benefits of diet, exercise, consuming less alcohol and smoking cessation for reducing risk for heart attack and stroke<br>**Reverse coded**<br>4 = Strongly disagree; 3 = disagree; 2 = agree; 1 = strongly agree; N/A = 0
		39. Increasing my exercise to at least 2 ½ hours a week will decrease my chances of having a heart attack or stroke.	
		40. When I exercise for at least 2 ½ hours a week I am doing something good for the health of my heart.	
		41. When I eat at least five portions of fruit and vegetables a day I am doing something good for the health of my heart.	
	**Barriers** (2 items)	42. I do not have time to exercise on most days of the week.	Higher average score = Higher perception of select barriers to engaging in heart attack and stroke risk reducing behaviours<br>1 = Strongly disagree; 2 = disagree; 3 = agree; 4 = strongly agree; N/A = 0
		43. I cannot afford to buy healthy foods.	
**Self**	**CVD risk**	<u>How confident are you that you know how to or can…</u>	Higher average score = higher perceived confidence

For peer review only - http://bmjopen.bmj.com/site/about/guidelines.xhtml

**Efficacy**<br><br>(5 items)	**reduction**<br><br>**self efficacy**<br><br>(5 items)	44. Control the risks of having a heart attack or stroke.	1 = Not at all confident; 2 = somewhat confident; 3 = very confident; 4 = completely confident; N/A = 0
		45. Maintain a healthy weight by exercising at least 2½ hours a week within the next two months.	
		46. Stop smoking if you want to.	
		47. Drink within the recommended levels of alcohol.	
		48. Eat at least five portions of fruit and vegetables per day within the next two months.	
**Intention /**<br><br>**Readiness**<br><br>**to Change**<br><br>(17 items)	**Exercise**<br><br>(4 items)	49. I am not thinking about exercising for 2 ½ hours a week.	Higher average score = Higher perceived readiness for change with regard to exercise behaviour<br><br>1 = Strongly disagree; 2 = disagree; 3 = agree; 4 = strongly agree; N/A = 0
		50. I am thinking about exercising at least 2 ½ hours a week.	
		51. I intend or want to exercise at least 2 ½ hours a week.	
		52. I am ready or have started to exercise 2 ½ hours a week.	
	**Diet**<br><br>(4 items)	53. I am not thinking about eating at least five portions of fruit and vegetables a day.	Higher average score = Higher perceived readiness for change with regard to health dietary behaviour<br><br>1 = Strongly disagree; 2 = disagree; 3 = agree; 4 = strongly agree; N/A = 0
		54. I am thinking about eating at least five portions of fruit and vegetables a day.	
		55. I intend or want to eat at least five portions of fruit and vegetables a day.	
		56. I am ready or started to eat at least five portions of fruit and vegetables a day.	
	**Alcohol**<br><br>(4 items)	57. I am thinking about cutting down on alcohol.	Higher average score = Higher perceived readiness for change with regard to alcohol consumption behaviour<br><br>1 = Strongly disagree; 2 = disagree; 3 = agree; 4 = strongly agree; N/A = 0
		58. I intend or want to cut down on alcohol.	
		59. I have been cutting down on alcohol.	
		60. I am not thinking about cutting down on alcohol.	
	**Smoking**<br><br>(5 items)	61. I am thinking of stopping smoking within two months.	Higher average score = Higher perceived readiness for change with regard to
		62. I have reduced or stopped smoking.	

			smoking cessation behaviour<br><br>1 = Strongly disagree; 2 = disagree; 3 = agree; 4 = strongly agree; N/A = 0
		63. I intend or want to stop smoking.	
		64. If I stop smoking it will reduce my chances of having a heart attack or stroke.	
		65. I am not thinking about stopping smoking.	

For peer review only

Appendix C. Population characteristics of 110 NHS Health Check attendees

For peer review only - http://bmjopen.bmj.com/site/about/guidelines.xhtml

Population Characteristics		n	% Total
Gender	Male	51	46.4%
	Female	56	50.9%
Age group	40-49	45	40.9%
	50-59	40	36.4%
	60-74	14	12.7%
Ethnicity	White	93	84.5%
	Mixed	2	1.8%
	Asian	2	1.8%
	Black	4	3.6%
	Other	4	3.6%
*Deprivation**	IMD 1 - least deprived	14	12.7%
	IMD 2	30	27.3%
	IMD 3	12	10.9%
	IMD 4	31	28.2%
	IMD 5 - most deprived	14	12.7%
Cholesterol	Raised total cholesterol TC≥ 5 mmol/l	66	60.0%
Blood pressure	High blood pressure BP ≥ 140/90 mm Hg	28	25.5%
Body Mass Index (BMI)	Obese (BMI>=30)	26	23.6%
Physical activity	Physically inactive	22	20.0%
Smoking status	Smokers	20	18.2%
Alcohol consumption	Excessive drinkers	13	11.8%
*10 year predicted risk of CVD***	High CVD Risk (QRisk2>=20%)	5	4.5%
	Medium CVD Risk (10%=<Qrisk2<20%)	21	19.1%
	Low CVD Risk (QRisk2<10%)	85	77.3%
Mean Values & 95% Confidence Intervals	Age (95% CI)	51.52	(49.93, 53.12)
	Total Cholesterol (95% CI)	5.42	(5.19, 5.64)
	HDL Cholesterol (95% CI)	1.44	(1.36, 1.53)
	Cholesterol Ratio (TC/HDL) (95% CI)	4.12	(3.73, 4.52)
	SBP (95% CI)	129.60	(125.76, 133.44)
	DBP (95% CI)	81.63	(79.62, 83.63)
	BMI (95% CI)	27.24	(26.17, 28.31)
	Q-Risk 2 (95% CI)	6.27	(5.19, 7.34)

*****Deprivation was measured using the Index of Multiple Deprivation (IMD).

**Ten year predicted risk of CVD was estimated using the Q-Risk 2 algorithm[1]

Notes: SBP = systolic blood pressure; DBP = diastolic blood pressure; CI = confidence interval; HDL = high density lipoprotein; CVD = cardiovascular disease

References

1. Hippisley-Cox J, Coupland C, Vinogradova Y, et al. Predicting cardiovascular risk in England and Wales: prospective derivation and validation of QRISK2. *BMJ*. 2008; 336.

Appendix D

For peer review only - http://bmjopen.bmj.com/site/about/guidelines.xhtml

The ABCD Risk Questionnaire and scoring guide

Scale	Items	Coding
**Knowledge**<br><br>Higher sum score = more knowledgeable / more correct about having a heart attack or stroke	1. One of the main causes of heart attack and stroke is stress.<br><br>2. Walking and gardening are considered types of exercise that can lower the risk of having a heart attack or stroke.<br><br>3. Moderately intense activity of 2 ½ hours a week will reduce your chances of having a heart attack or stroke.<br><br>4. People who have diabetes are at higher risk of having a heart attack or stroke.<br><br>5. Managing your stress levels will help you to manage your blood pressure.<br><br>6. Drinking high levels of alcohol can increase your cholesterol and triglyceride levels.<br><br>7. HDL refers to 'good' cholesterol, and LDL refers to 'bad' cholesterol.<br><br>8. A family history of heart disease is not a risk factor for high blood pressure.	Correct Answers:<br><br>Q1-T<br><br>Q2-T<br><br>Q3-T<br><br>Q4-T<br><br>Q5-T<br><br>Q6-T<br><br>Q7-T<br><br>Q8-F<br><br>T= True<br>F= False<br><br>Correct: Score = 1,<br>Incorrect or Don't Know: Score = 0.
**Perceived Risk of**<br><br>**Heart**<br><br>**Attack/Stroke**	9. I feel I will suffer from a heart attack or stroke sometime during my life.	1=Strongly disagree; 2 = disagree; 3 = agree; 4 = strongly agree; N/A = 0
	10. It is likely that I will suffer from a heart attack or stroke in the future.	1=Strongly disagree; 2 = disagree; 3 = agree; 4 = strongly agree; N/A = 0
Higher sum score = higher perception of risk of having a heart attack or stroke	11. It is likely that I will have a heart attack or stroke some time during my life.	1=Strongly disagree; 2 = disagree; 3 = agree; 4 = strongly agree; N/A = 0
	12. There is a good chance I will experience a heart attack or stroke in the next 10 years.	1=Strongly disagree; 2 = disagree; 3 = agree; 4 = strongly agree; N/A = 0
	13. My chances of suffering from a heart attack or stroke in the next 10 years are great.	1=Strongly disagree; 2 = disagree; 3 = agree; 4 = strongly agree; N/A = 0
**Perceived Risk of**	14. It is likely I will have a heart attack or stroke because of my past and/or present behaviours.	1=Strongly disagree; 2 = disagree; 3 = agree; 4 = strongly agree; N/A = 0

For peer review only - http://bmjopen.bmj.com/site/about/guidelines.xhtml

Scale	Items	Coding
**Heart Attack/Stroke**	15. I am not worried that I might have a heart attack or stroke.	**Reverse coded**<br>4=Strongly disagree; 3 = disagree; 2 = agree; 1 = strongly agree; N/A = 0
	16. I am concerned about the likelihood of having a heart attack or stroke in the near future.	1=Strongly disagree; 2 = disagree; 3 = agree; 4 = strongly agree; N/A = 0
**Perceived Benefits and Intentions to Change**<br>Higher average score = Higher perceived benefits of diet and exercise and higher perceived readiness for change in regards to exercise behaviour	17. I am thinking about exercising at least 2½ hours a week.	1=Strongly disagree; 2 = disagree; 3 = agree; 4 = strongly agree; N/A = 0
	18. I intend or want to exercise at least 2½ hours a week.	1=Strongly disagree; 2 = disagree; 3 = agree; 4 = strongly agree; N/A = 0
	19. When I exercise for at least 2½ hours a week I am doing something good for the health of my heart.	1=Strongly disagree; 2 = disagree; 3 = agree; 4 = strongly agree; N/A = 0
	20. I am confident that I can maintain a healthy weight by exercising at least 2½ hours a week within the next two months.	1=Strongly disagree; 2 = disagree; 3 = agree; 4 = strongly agree; N/A = 0
	21. I am not thinking about exercising for 2 ½ hours a week.	**Reverse coded**<br>4=Strongly disagree; 3 = disagree; 2 = agree; 1 = strongly agree; N/A = 0
	22. When I eat at least five portions of fruit and vegetables a day I am doing something good for the health of my heart.	1=Strongly disagree; 2 = disagree; 3 = agree; 4 = strongly agree; N/A = 0
	23. Increasing my exercise to at least 2½ hours a week will decrease my chances of having a heart attack or stroke.	1=Strongly disagree; 2 = disagree; 3 = agree; 4 = strongly agree; N/A = 0
**Healthy Eating Intentions**<br>Higher average score = Higher perceived readiness for change with regard to health dietary behaviour	24. I am confident that I can eat at least five portions of fruit and vegetables per day within the next two months.	1=Strongly disagree; 2 = disagree; 3 = agree; 4 = strongly agree; N/A = 0
	25. I am thinking about eating at least five portions of fruit and vegetables a day.	1=Strongly disagree; 2 = disagree; 3 = agree; 4 = strongly agree; N/A = 0
	26. I am not thinking about eating at least five portions of fruit and vegetables a day.	**Reverse coded**<br>4=Strongly disagree; 3 = disagree; 2 = agree; 1 = strongly agree; N/A = 0

The Attitudes and Beliefs about Cardiovascular Disease (ABCD) Risk Questionnaire is licensed under the Creative Commons Attribution-NonCommercial-NoDerivatives 4.0 International License.

For peer review only - http://bmjopen.bmj.com/site/about/guidelines.xhtml

For peer review only

For peer review only - http://bmjopen.bmj.com/site/about/guidelines.xhtml

BMJ Open

Development of a questionnaire to evaluate patients' awareness of cardiovascular disease risk in England's National Health Service Health Check preventive cardiovascular programme

Journal:	*BMJ Open*
Manuscript ID	bmjopen-2016-014413.R2
Article Type:	Research
Date Submitted by the Author:	03-Jul-2017
Complete List of Authors:	Woringer, Maria; Imperial College London Department of Primary Care and Public Health, 3rd Floor Reynolds Building Charing Cross Campus St Dunstans Road, W6 8RP<br>Nielsen, Jessica; City University London, School of Arts and Social Sciences, Department of Psychology, City University London Northampton Square, EC1V 0HB<br>Zibarras, Lara; City University London, School of Arts and Social Sciences, Department of Psychology, City University London Northampton Square, EC1V 0HB<br>Evason, Julie; Health Diagnostics Ltd, Chatham House, Dee Hills Park, CH3 5AR<br>Kassianos, Angelos; University College London, Department of Applied Health Research<br>Harris, Matthew; Imperial College London, Institute of Global Health Innovation, Level 1, Faculty Building South Kensington Campus, SW7 2NA ; Imperial College London Department of Primary Care and Public Health, 3rd Floor Reynolds Building Charing Cross Campus St Dunstans Road, W6 8RP<br>Majeed, Azeem; Imperial College London, Primary Care & Public Health, 3rd Floor Reynolds Building Charing Cross Campus St Dunstans Road, W6 8RP<br>Soljak, Michael; Imperial College London, Primary Care & Public Health, 3rd Floor Reynolds Building Charing Cross Campus St Dunstans Road, W6 8RP
**Primary Subject Heading**:	Communication
Secondary Subject Heading:	General practice / Family practice, Health services research
Keywords:	QUALITATIVE RESEARCH, PREVENTIVE MEDICINE, PRIMARY CARE, PUBLIC HEALTH

SCHOLARONE™
Manuscripts

For peer review only

Development of a questionnaire to evaluate patients' awareness of cardiovascular disease risk in England's National Health Service Health Check preventive cardiovascular programme

Author Names and Affiliations:

Ms. Maria Woringer (a)

Dr. Jessica Jones Nielsen (b)

Dr. Lara Zibarras (b)

Ms. Julie Evason (c)

Dr. Angelos P. Kassianos (d)

Dr. Matthew Harris (e)

Professor Azeem Majeed (a)

Dr. Michael Soljak (a)

Affiliations :

a) Department of Primary Care and Public Health, Imperial College London

b) Department of Psychology, City University London

c) Health Diagnostics Ltd, Chester, UK

d) Department of Applied Health Research, University College London

e) Institute of Global Health Innovation, Imperial College London

Word Count (excluding title page, declarations, abstract, keywords, strengths and limitations and references): 3,999

Author responsible for correspondences concerning the manuscript:

Maria Woringer; Imperial College London, Department of Primary Care and Public Health, Charing Cross Campus, The Reynolds Building, St Dunstan's Road, London W6 8RP, UK m.woringer@imperial.ac.uk; Tel: +44(0)2075940789

For peer review only - http://bmjopen.bmj.com/site/about/guidelines.xhtml

For peer review only

List of Declarations:

Dissemination of Study Findings

The work on the development and validation of the patient questionnaire was presented as a poster titled "Development and Validation of the Attitudes and Beliefs about Cardiovascular Disease (ABCD) Risk Survey" at the NHS Health Check 2015 – Improvement through Collaboration conference in Leeds, England on 26 February, 2015. In addition, an abstract titled "Development and validation of a patient survey to assess the effectiveness of cardiovascular disease screening" was selected for oral presentation at the First International Conference of Public Health, Primary Care and Congress of Person Centred Medicine on October 29, 2015 and accepted for publication in the International Journal of Person Centred Medicine. This submission is not under consideration by any other journal. All authors have approved the manuscript and this submission.

For peer review only

Permissions

Favourable ethical opinion for the study - "Patient Evaluation of the NHS Health Check Programme to Investigate the Programme's Effectiveness in Communicating CVD Risk" was obtained from the NRES Committee London – City & East reference number 13/LO/1885.

Study participants gave their written informed consent to participate in the study and to share their results and medical data.

The Attitudes and Beliefs about Cardiovascular Disease (ABCD) Risk Questionnaire is licensed under the Creative Commons Attribution-NonCommercial-NoDerivatives 4.0 International License.

Funding

This work was supported by the National Institute for Health Research (NIHR) Diagnostics Evidence Co-operatives (DEC) / Collaboration for Leadership in Applied Health Research and Care (CLAHRC) grant.

Data Sharing Agreement

No additional data available.

Competing Interests

None.

Contributors

MW, AM, MS, and HW designed the study, JE supplied the data. JJN designed the validation instrument, LZ performed the psychometric analysis. JE, AK, MH and AM reviewed the validation instrument's face and content validity. All authors discussed data analyses and interpreted the results. MW wrote the first draft of the manuscript. All authors critically revised and approved the final manuscript. MW had full access to all the data used in the study and takes responsibility for the integrity of the data and the accuracy of the data analysis. MW is the guarantor.

For peer review only - http://bmjopen.bmj.com/site/about/guidelines.xhtml

ABSTRACT

Background

The National Health Service (NHS) Health Check is a CVD risk assessment and management programme in England aiming to increase CVD risk awareness among people at increased risk of CVD. There is no tool to assess the effectiveness of the programme in communicating CVD risk to patients.

Aims

The aim of this paper was to develop a questionnaire examining patients' CVD risk awareness for use in health service research evaluations of the NHS Health Check programme.

Methods

We developed an 85 item questionnaire to determine patients' views of their risk of CVD. The questionnaire was based on a review of the relevant literature. After review by an expert panel and focus group discussion, 22 items were dropped and 2 new items were added. The resulting 65 item questionnaire with satisfactory content validity (content validity indices >=0.80) and face validity was tested on 110 NHS Health Check attendees in primary care in a cross sectional study between May 21 and July 28, 2014.

Results

Following analyses of data, we reduced the questionnaire from 65 to 26 items. The 26 item questionnaire constitutes 4 scales: Knowledge of CVD Risk and Prevention, Perceived Risk of Heart Attack/Stroke, Perceived Benefits and Intention to Change Behaviour and Healthy Eating Intentions. Perceived Risk (Cronbach's α = 0.85) and Perceived Benefits and Intention to Change Behaviour (Cronbach's α = 0.82) have satisfactory reliability (Cronbach's α >=0.70). Healthy Eating Intentions (Cronbach's α = 0.56) is below minimum threshold for reliability but acceptable for a three item scale.

Conclusions

The final questionnaire, with satisfactory reliability and validity, is recommended for use in assessing patients' awareness of CVD risk among NHS Health Check attendees.

Word Count: 276

Keywords: cardiovascular disease, primary prevention, risk assessment, questionnaire

Strengths and limitations of this study

- Questionnaire guided by literature review, expert panel, patient focus group & data analysis
- Largely developed among 110 individuals representative of the target population
- Face validity assessed via a patient focus group not representative of the target population

For peer review only - http://bmjopen.bmj.com/site/about/guidelines.xhtml

INTRODUCTION

Cardiovascular disease (CVD) is a major cause of disability and premature mortality worldwide. In England it accounts for a third of deaths and costs the NHS and UK economy £30 billion annually.[1, 2] Modifiable lifestyle risk factors, associated with 90% of CVD,[3, 4] contributed to only 34% of the overall decline in CVD mortality in England between 2000-2007.[5] In 2010 / 2011 there were 1.4 million CVD related hospital admissions, of which 60% were for people younger than 75 and more than half as an emergency. Further gains could be made in preventing long term illness and disability associated with CVD while reducing healthcare costs by promoting healthier lifestyle changes.[6]

The National Health Service (NHS) Health Check programme may be important for preventing premature CVD while reducing healthcare costs therein by identifying individuals at increased risk of CVD, raising their awareness of CVD risk and helping them manage their risk. [7-10] This CVD risk assessment and management programme was launched by the Department of Health in April 2009 in England among 40-74 year olds free of vascular disease diagnosis.[7] It aims to prevent heart disease, stroke, diabetes and kidney disease whilst reducing health inequalities. Individuals' socio-demographics, cholesterol, blood pressure, smoking, and family history of CVD are used to predict CVD risk.[11] In addition to lifestyle advice given to all participants, people at high risk of CVD are invited for further consultations and offered statins and behaviour change support in relation to physical activity, smoking cessation, safe alcohol consumption and healthy diet. Projected programme cost is £180-£243 million/year with estimated cost per quality adjusted life year (QALY) at £3,000.[10]

To adopt healthy lifestyle behaviours related to diet, exercise, smoking and alcohol consumption, the general population must be aware of CVD risk.[12] In the context of the NHS Health Check Programme, CVD risk awareness refers to the accuracy of perceived risk of CVD against predicted CVD risk, general knowledge of CVD and what one can do to lower predicted CVD risk. Whereas predicted CVD risk refers to one's chance of experiencing a heart attack or stroke,[11] perceived risk of CVD refers to a person's perception of their CVD risk. While as many as 40% of the general population underestimate their CVD risk, 20% overestimate their risk.[13] False reassurance may lead to adoption and or maintenance of unhealthy behaviours contributing to the premature onset of CVD. Low CVD risk awareness is reported among men, inner city residents, and people of lower socioeconomic status. [12 14 15] It is not known if the Health Check results in improved CVD risk awareness.

Although several validated questionnaires measure knowledge, perceptions of CVD or intention to change behaviour, [15-17] no short, validated questionnaire assesses CVD risk awareness using all of these scales. Until now studies examining the accuracy of perceived risk and knowledge of CVD relied on non-validated tools.[16] The problem with using non-validated tools is that the questions may not accurately and reliably capture individuals' views or measure what they intend to measure. The aim of this work was to develop a questionnaire with satisfactory face, content validity and reliability to assess patients' awareness of CVD risk among NHS Health Check attendees.

METHODS

For peer review only - http://bmjopen.bmj.com/site/about/guidelines.xhtml

The first phase of development of the questionnaire was guided by a literature review, an expert panel and a patient focus group. At each stage of questionnaire development, the number of items was reduced (see Figure 1).

Figure 1 Flowchart of Phase I of Questionnaire Development

The second phase of questionnaire development was guided by an analysis of data from 110 NHS Health Check attendees who completed the 65 item questionnaire. The number of questionnaire items was further reduced (see Figure 2).

Figure 2 Flowchart of Phase II of Questionnaire Development

Phase I of Questionnaire Development

Construction of draft questionnaire by review of relevant literature

We performed an extensive literature review pertaining to CVD risk awareness between December 2013 and January 2014 in the areas of disease knowledge, risk perception, intention to change and self-efficacy related to CVD and the Health Belief Model (HBM) to guide initial item development. PubMed and PsycINFO databases and Google Scholar Articles were utilised to search for existing instruments that measure perception of CVD risk, CVD knowledge and self-efficacy with no limits on the year of publication. The following key words were used to identify the relevant literature: "cardiovascular disease" "heart disease" "knowledge" "risk" "test" "questionnaire" "scale" "assessment" "self-efficacy" "perception" "health belief model". Questionnaires were considered if they addressed CVD risk awareness, reported moderate to high scores of reliability and validity in population studies and had suitable wording and level of understanding. Questionnaires were excluded if they pertained to individuals under the age of 15 as these people would not be eligible to receive an NHS Health Check, focused on risk unrelated to heart attack or stroke, and were not written in English.

Although a number of questionnaires were found measuring different aspects of CVD risk awareness such as heart disease knowledge, perception of CVD risk, perceived susceptibility and severity of CVD and benefits and barriers to adopting healthy behaviours,[17-19] no single questionnaire encompassed them all. Initial item development was guided by HBM[20] and the Transtheoretical Model (TTM).[21] According to HBM, individuals who have accurate knowledge of CVD and perceived susceptibility to and consequences of the disease, and are aware of the benefits of taking preventive measures are more likely to make important lifestyle choices to prevent the onset of disease.[22] The TTM describes behavioural change as a staged process over time including pre-contemplation, contemplation, preparation, action and maintenance.[21] Sixty five items were selected using validated questionnaires addressing CVD knowledge, and the main constructs of HBM such as perceived susceptibility, perceived severity, perceived benefits of changing behaviours, and perceived barriers to making changes.[17-19] In addition 23 new items were generated to identify perceived levels of readiness to engage in CVD risk reduction behaviours (using TTM) and self-efficacy (confidence in ability to change health behaviour) in relation to exercise, diet, smoking cessation and decreasing alcohol consumption.[23, 24] These items were based on data collected during an NHS Health Check and behaviour specific recommendations such as stopping smoking, consuming no more than 14 units of

For peer review only - http://bmjopen.bmj.com/site/about/guidelines.xhtml

alcohol a week, eating at least five portions of fruit and vegetables a day and exercising at least 150 minutes per week.[25-28] The resulting 85 item questionnaire is in Appendix A.

Modification of questionnaire by expert panel to obtain satisfactory content validity

A panel of experts in the areas of CVD, health psychology, public health, psychometrics and questionnaire development and medicine were asked to evaluate each item and the total 85 item questionnaire for content validity in February 2014. Experts assessed content validity of the questionnaire by examining whether the items were representative of the content they were intended to measure.[29] Items were examined for representatives of the scale domain, appropriateness and relevance. The content validity index (CVI), a widely used technique in scale development determined item and questionnaire clarity, homogeneity, and relevance on a 4-point Likert scale (ranging from 1 = *an irrelevant item* to 4 = *an extremely relevant item*).[30, 31] A CVI of ≥ 0.80 is recommended.[32, 33] Experts were asked the following questions: "*Do these items belong together in the subscale?*" and "*Does each item belong in the set?*" For ratings of content validity, experts were asked whether the subscale definition and label fitted the set of items presented; whether each item belonged with the label and definition; and whether each item was unique in its contribution to the subscale.

Modification of questionnaire by patient focus group to obtain satisfactory face validity

Researchers facilitated a patient focus group to assess the face validity of the 69 item questionnaire resulting from the expert review. Face validity is assessed by end users deciding whether the questionnaire appears to measure what the researchers who developed it claim.[33] A convenience sample of six individuals was recruited on March 4, 2014 from the County Durham and Darlington National Health Service Foundation Trust. Eligibility criteria were being aged 40-74 years and being free of known vascular disease. The focus group consisted of six white females between 50-64 years of age. Most participants had postgraduate education. These individuals worked as clerical workers, nurses and health improvement staff. They were not involved in the delivery of the NHS Health Check programme. Participants were asked to complete the 69 item questionnaire as well as to provide feedback on whether the items correctly measured the intended scales, appropriately stated the intent of the questionnaire, and matched the individual's situations.[32, 33] In addition, participants were asked to respond to questions about clarity, content, appropriateness, format, biases of questions and presentation of information. The resulting 65 item questionnaire is in Appendix B.

Phase II of Questionnaire Development

Modification of questionnaire to have satisfactory reliability

A 65 item questionnaire was administered to 110 NHS Health Check attendees immediately after their consultation between May 21 and July 28, 2014 in a cross sectional study in England. The aim was to determine the content, the scale structure and the reliability of a questionnaire in its final form.

Study Recruitment

Eligibility criteria were completion of an NHS Health Check, being aged 40-74 years and free of known vascular disease. Of 110 study participants, 15 individuals were recruited by 2 nurses from a

For peer review only - http://bmjopen.bmj.com/site/about/guidelines.xhtml

London general practice and 95 individuals by 13 community outreach providers from local community venues in Durham. These providers collected clinical risk factor data, informed study participants about their CVD risk, took informed study consent and distributed the 65 item questionnaire to be self-completed by NHS Health Check attendees following their consultation. Unlike general practice staff who operated only during business hours, community outreach providers worked on evenings and weekends as well as during regular business hours in community venues more accessible to the general public.

Data Analysis

To select appropriate items to constitute a scale, individual items were assessed during item analysis, item facility and item discrimination.[34] To determine the factorial structure of the questionnaire and which items together constituted particular scales, an Exploratory Factor Analysis (EFA) - a widely used technique in scale development was performed.[30, 35] The reliability of factors constituting particular scales was assessed using Cronbach's alpha coefficient. [36, 37] Reliability refers to consistency, reproducibility and agreement of a scale.[38]

To improve the quality of a scale and increase its reliability, individual items were assessed. Items with reverse scoring were re-coded to conform to the conceptual direction of the scales.[37] Each individual item was then examined for distortions in the pattern of responding known as skew and kurtosis.[33] Item facility examined whether items were answered in the same way by everyone by checking whether the facility index approached extreme scores or had a low standard deviation.[34] Items were assessed in discriminating between participants' responses to the questionnaire's scales (Knowledge, Perceived CVD Risk, CVD Health Beliefs, Intentions / Readiness to Change and Self Efficacy). Discrimination was measured by item-total correlation with item correlating below 0.2 or any negative correlations resulting in deletion of items. In addition, discrimination was measured by the inter-item correlation within each scale resulting in deletion of items correlating with other items ≥0.60.[17, 34]

A Kaiser-Meyer-Olkin (KMO) measure of sampling adequacy and a Bartlett's test of sphericity were assessed to ensure that items were appropriate for EFA.[39] Next EFA was performed to define the scales of the questionnaire which share a similar underlying construct. Parallel Analysis was used to determine the optimum number of factors to be extracted using Principal Components Analysis (PCA) with a Varimax rotation. [34, 39, 40] PCA is a data reduction technique used to explain correlations among sets of items or variables as a few conceptually meaningful factors.[30] Compared to other available methods, Parallel Analysis using PCA was shown to be the best method of extracting factors and is appropriate when applied to data conforming to the formal factor analytic model.[39, 40] Iterations of EFA were carried out to identify core constituent items in each factor. Cross-loading items or items with loading <=0.50 were removed at each iteration.[39] Internal consistency reliability of resulting factors was assessed using Cronbach's α coefficients with α>=0.70 indicating good reliability.[32,36, 37] Associations between resulting factors and predicted CVD risk were examined using Spearman's rank correlation coefficient.

RESULTS

Construction of a draft questionnaire by review of relevant literature

For peer review only - http://bmjopen.bmj.com/site/about/guidelines.xhtml

We developed an 85 item questionnaire based on the theoretical framework, NHS guidelines and other validated questionnaires relating to heart disease.[17-19] The 85 item questionnaire had 8 subscales measuring *Knowledge of CVD Risk and Prevention* (18 items), *Perceived Risk and Vulnerability of CVD* (20 items), *Perceived Susceptibility* (5 items), *Perceived Severity* (5 items), *Perceived Benefits* (6 items), *Perceived Barriers* (7 items), *Self-Efficacy* (6 items), and *Intention to Change Behaviour* (18 items). *Knowledge of CVD Risk and Prevention* subscale items were measured using the following categories: *True, False*, and *Don't Know*. *Self-Efficacy* subscale items were measured using 5 point Likert scale ranging from *1=not at all confident* to *5=completely confident*. *Perceived Severity, Perceived Benefits, Perceived Barriers and Intention to Change Behaviour* subscale items were measured using a 4 point Likert scale ranging from *1=strongly disagree* to *4=strongly agree*. The reading level of the questionnaire was at Year 7.

Modification of questionnaire by expert panel to obtain satisfactory content validity

The expert panel concluded that out of the 85 items, 69 met the CVI>=0.80 criterion and were retained. In addition, the wording of a number of questions was revised to improve clarity. Diet and exercise were defined more precisely using frequency and duration. Response options of Self-Efficacy items were changed from a five point Likert scale to a four point Likert scale for consistency with the rest of the questionnaire. Questions pertaining to smoking and drinking were rephrased to apply to smokers and drinkers (see Table 1).

Table 1 Sample item wording modifications obtained through an expert panel

**Original item(s)**	**Expert comments**	**Final item**
The most important cause of heart attack and stroke is stress.	Revise to "one of the most important…" Substitute the word "important" with "main."	One of the main causes of heart attack and stroke is stress.
I have a high chance of getting a heart attack or stroke because of my past behaviours.	Add "and/or present behaviours."	I have a high chance of getting a heart attack or stroke because of my past and/or present behaviours.
Increasing my exercise will decrease my chances of having a heart attack or stroke.	Define amount of exercise.	Increasing my exercise to at least 30 minutes a day will decrease my chances of having a heart attack or stroke.
Eating a healthy diet will decrease my chance of having a heart attack or stroke.	Define a healthy diet.	Eating at least five portions of fruit and vegetables a day will decrease my chances of having

For peer review only - http://bmjopen.bmj.com/site/about/guidelines.xhtml

Original item(s)	Expert comments	Final item
		a heart attack or stroke.
When I exercise I am doing something good for myself.	Define exercise consistently.<br>Make the statement more specific about the heart.	When I exercise for 30 minutes a day I am doing something good for the health of my heart.
How confident are you that you know or can…? questions answered using a 5-point Likert scale:<br>“not at all confident, somewhat confident, moderately confident, very confident, completely confident.”	Use a 4-point Likert to maintain consistency.	Five point Likert scale changed to a 4 point Likert scale:<br>“not at all confident, somewhat confident, very confident, completely confident.”
How confident are you that you know how or can stop smoking if you want to?	Instead of saying “…that you know or can” say “that you know how to or can…”<br>Add in parentheses “if you smoke.”	How confident are you that you know how to or can stop smoking if you want to (if you smoke)?
I want to cut down on alcohol.<br>I intend to cut down on alcohol in the next two months.	Conceptual overlap between want to and intend to.<br>Add in parentheses “if you drink alcohol.”	I intend or want to cut down on alcohol (if you drink alcohol).

Modification of questionnaire by patient focus group to obtain satisfactory face validity

As a result of the focus group review of the 69 item questionnaire, six items were removed, two items were added and a number of items were modified leaving a final total of 65 items with satisfactory face validity. A not applicable category was added to 50 items while the response categories to Knowledge subscale items remained unchanged. Exercise was redefined in 8 items from 150 minutes a week and 30 minutes a day to 2.5 hours a week. A negatively framed question was reframed positively (see Table 2).

Table 2 Sample item wording modifications and additions through the patient focus group

Original item	Participant comments	Final item
Moderate physical activity of 150 minutes a week will	2.5 hours a week is better than 150 minutes.	Moderate physical activity of 2.5 hours a week will reduce

For peer review only - http://bmjopen.bmj.com/site/about/guidelines.xhtml

Original item	Participant comments	Final item
reduce your chances of developing a heart or stroke.		your chances of developing a heart or stroke.
Drinking alcohol has nothing to do with reducing the risk of heart attack or stroke.	Question is negatively stated.	Drinking high levels of alcohol can increase your cholesterol and triglyceride levels.
Missing question	Need to include family history of disease to account for genetic predisposition.	A family history of hypertension is not a risk factor for high blood pressure.
Missing question	Benefits of not smoking?	If I stopped smoking it will reduce my chances of having a heart attack or stroke.
Increasing my exercise for 30 minutes a day will decrease my chances of having a heart attack or stroke.	Two and a half hours a week is better than 30 minutes a day.	Increasing my exercise to at least 2 ½ hours a week will decrease my chances of having a heart attack or stroke.
I have reduced or stopped smoking (if you smoke). “strongly disagree, disagree, agree, and strongly agree.”	Remove (if you smoke). Add a “not applicable” box.	I have reduced or stopped smoking. “strongly disagree, disagree, agree, and strongly agree, not applicable.”
How confident are you that you know how to or can consume recommended levels of alcohol (if you drink alcohol)? “not at all confident, somewhat confident, very confident and completely confident.”	Remove (if you drink alcohol). Add a “not applicable” box.	How confident are you that you know how to or can drink within the recommended levels of alcohol? “not at all confident, somewhat confident, very confident and completely confident, not applicable.”

Modification of questionnaire to have satisfactory reliability

The 65 item questionnaire that resulted from content and face validity assessments, was administered to 110 NHS Health Check attendees immediately after their NHS Health Check consultation. Most study participants were White (84.5%), younger than 60 (77.3%) and had at least

For peer review only - http://bmjopen.bmj.com/site/about/guidelines.xhtml

one or more CVD risk factors. Using the Index of Multiple Deprivation, a relative measure of deprivation across seven distinct domains including income, employment, health and disability, education skills and training, barriers to housing and services, living environment and crime, [41] people in the two most deprived fifths were 40.0% of the study population. See Appendix C for study population characteristics. The responses to the questionnaire were analysed as individual items during item analysis, item facility and item discrimination. In addition, the scale structure and reliability of resulting scales were assessed.

No items were removed during item analysis and item facility. During item discrimination assessment using item-total correlation, seven items in the Knowledge scale, four items in Perceived CVD Risk, three items in CVD Health Benefits, three items in Intention and or Readiness to Change were deleted due to item-total correlations falling below 0.2. [33] During item discrimination assessment using inter-item correlation, two items in Perceived CVD Risk and three items in Intentions / Readiness to Change were removed as these items correlated greater than 0.6 with other items.[33] Although there were two items that correlated above 0.6 in CVD Risk Reduction Self Efficacy, these remained in the questionnaire as the items were qualitatively different: *Stop smoking if you want to* and *Control the risks of having a heart attack or stroke.* In total, 22 items were removed during item discrimination analysis, leaving 43 items which had good item facility and discrimination.

Of the 43 remaining items, 8 items of the "Knowledge" scale with *"true"* or *"false"* scoring could not be entered into EFA. Of the 35 items scored on a four point Likert scale, four items pertaining to smoking were deleted as they had a high proportion of missing responses (69-80%). The resulting 31 items had a Kaiser-Meyer-Olkin (KMO) measure of sampling adequacy of 0.32 and a significant Bartlett's test of sphericity (1020.50, p <.001), indicating that these data were appropriate for EFA.[39] After 12 iterations of EFA, 20 items loaded above 0.50 on the factors and there were no cross-loadings indicating good factor structure (see Table 3). Internal consistency reliability of factor structure was measured using Cronbach's α. Factor 1 (8 items): (Perceived Risk of Heart Attack/Stroke) had α =.85. Factor 2 (7 items): (Perceived Benefits & Intentions to Change) had α =.82. Factor 3 (3 items): (Healthy Eating Intentions) had α =.56. Factor 4 (2 items): (Intentions towards Alcohol) had α =-0.16. Although Healthy Eating Intentions α = 0.56 is below the minimum threshold (0.70) for reliability, this is acceptable for a three item scale.[34] The intention toward alcohol factor had two items with such low reliability (α = -0.16) that they could not be considered a separate factor and were removed. A thirteenth EFA iteration confirmed the factor loadings and reliabilities reported above. Hence the parallel analysis indicated that three factors should be retained. [39] The three factor model accounted for 57.61% of the total explained variance.

Table 3 Factor structure of the ABCD Risk Questionnaire

	Components

For peer review only - http://bmjopen.bmj.com/site/about/guidelines.xhtml

	Factor 1 Perceived Risk of Heart Attack / Stroke	Factor 2 Perceived Benefits & Intentions to Change	Factor 3 Healthy Eating Intentions
It is likely that I will suffer from a heart attack or stroke in the future.	.844		
It is likely that I will have a heart attack or stroke some time during my life.	.816		
I feel I will suffer from a heart attack or stroke sometime during my life.	.809		
There is a good chance I will experience a heart attack or stroke in the next 10 years.	.752		
I am not worried that I might have a heart attack or stroke.	.705		
My chances of suffering from a heart attack or stroke in the next 10 years are great.	.687		
It is likely I will have a heart attack or stroke because of my past and/or present behaviours.	.639		
I am concerned about the likelihood of having a heart attack or stroke in the near future.	.575		
I am thinking about exercising at least 2 ½ hours a week.		.826	
I intend or want to exercise at least 2 ½ hours a week.		.792	
When I exercise for at least 2½ hours a week I am doing something good for the health of my heart.		.735	
I am confident that I can maintain a healthy weight by exercising at least 2½ hours a week within the next two months.		.658	

For peer review only - http://bmjopen.bmj.com/site/about/guidelines.xhtml

	Components		
	Factor 1 Perceived Risk of Heart Attack / Stroke	Factor 2 Perceived Benefits & Intentions to Change	Factor 3 Healthy Eating Intentions
I am not thinking about exercising for 2 ½ hours a week.		.656	
When I eat at least five portions of fruit and vegetables a day I am doing something good for the health of my heart.		.642	
Increasing my exercise to at least 2½ hours a week will decrease my chances of having a heart attack or stroke.		.557	
I am confident that I can eat at least five portions of fruit and vegetables per day within the next two months.			.830
I am thinking about eating at least five portions of fruit and vegetables a day.			.772
I am not thinking about eating at least five portions of fruit and vegetables a day.			.731
Note: Factor loadings and commonalities are reported following an EFA using Principal Component Analysis with Varimax rotation.			

The EFA revealed three scales: Perceived Risk of Heart Attack / Stroke, Perceived Benefits and Intentions to Change and Healthy Eating Intentions. A fourth scale assessing Knowledge of CVD Risk and Prevention (not entered into EFA) was added back to the questionnaire following EFA (see Figure 2). Hence the final questionnaire included 26 items grouped into four scales: Knowledge of CVD Risk and Prevention (8 items), Perceived Risk of Heart Attack/Stroke (7 items), Perceived Benefits and Intention to Change Behaviour (7 items) and Healthy Eating Intentions (3 items). In the resulting 26 item questionnaire, two items were changed from questions "How confident are you that you know how to or can…" to statements of agreement "I am confident that I can" so as to be answered using the same Likert scale. The time to complete this questionnaire is between 10-15 minutes. The ABCD Risk Questionnaire with a scoring guide for each scale is reported in Appendix D. Using Spearman's rho, there was a positive and significant relationship between perceived and predicted CVD risk (Appendix E).

For peer review only - http://bmjopen.bmj.com/site/about/guidelines.xhtml

DISCUSSION

To the best of our knowledge this is the first study that describes the development of a short, validated questionnaire examining CVD risk awareness among the NHS Health Check attendees. Satisfactory content and face validity as well as reliability of the ABCD Risk Questionnaire suggest that the tool performs well. It may be used for evaluating the accuracy of perceived CVD risk, general knowledge of CVD and intention to change behaviour in regards to diet and exercise among NHS Health Check attendees. Agreement between perceived and predicted CVD risk suggests that the tool performs well in assessing perceived CVD risk. As the ABCD Risk Questionnaire was developed using both an expert panel and a patient focus group, the questions ought to be relatively easy to understand for both patients and clinicians. If NHS Health Check recommendations change over time, the ABCD Risk Questionnaire may need to be updated.

Critics of the NHS Health Check programme point to the lack of its evidence base. [42, 43] The majority of evaluations focused on coverage and uptake, statin prescribing, new diagnoses and CVD risk factor reduction. [44-49] As there was no instrument measuring CVD risk awareness, no studies examined the patients' understanding of CVD risk among NHS Health Check attendees. CVD risk presentation was shown to increase the accuracy of perceived risk by 10%. When risk information is repeated this leads to small but significant reductions in predicted CVD risk.[16] A national study showed modest reductions in 10 year predicted CVD risk among NHS Health Check attendees in the first four years.[48] A limitation of using predicted ten year risk of CVD is the under-estimation of CVD risk among women and younger people. [35] More research is needed to establish whether the programme improves NHS Health Check attendees' awareness of CVD risk and whether the programme has an impact on predicted lifetime CVD risk.

The ABCD Risk Questionnaire was developed and tested on a non-risk stratified population as the NHS Health Check programme is administered to all eligible people free of vascular disease diagnosis irrespective of their level of CVD risk. As such it does not encompass all aspects of CVD risk observed in the general population. Questions on smoking and drinking were progressively eliminated as they did not apply to most study participants. As questions on diet and exercise pertained to all people regardless of their level of CVD risk, such questions that reliably distinguished between study participants were selected for inclusion. Although fruit and vegetable intake is only one aspect of diet in the EatWell Guide recommended for use in NHS Health Check,[50] it is the only assessment of diet recorded during the NHS Health Check. The final questionnaire contains questions based on data collected during NHS Health Check to enable subsequent programme evaluation.[51] Future studies examining populations at increased CVD risk can look into incorporating smoking and alcohol into the ABCD Risk Questionnaire.

Judging by the number of items reduced in various stages of development, the final questionnaire was largely shaped by analysis of data from 110 NHS Health Check attendees completing the 65 item questionnaire. This study population was representative of the population that took up the NHS Health Check programme between 2009-2014 in terms of socio-demographics including the proportion of men (46.4%), ethnic minorities (5.4%), individuals from the most deprived two fifths (40.9%), and clinical risk factors including mean total cholesterol (5.42 (95% CI 5.19, 5.64)), BMI (27.24 95% CI 26.17, 28.31), smokers (18.2%) and those at high CVD risk (4.5%).[44] As higher levels of deprivation are partly due to having less education,[41] questionnaire development was not limited to

For peer review only - http://bmjopen.bmj.com/site/about/guidelines.xhtml

people with higher education. Compared to the national evaluation, similar levels of high CVD risk were observed despite the fact that the study population contained more younger people aged 40-59 (77.3%).[44] The recruitment of hard to reach groups including younger people, socio-economically deprived individuals and ethnic minorities by community outreach providers in community venues outside of conventional working hours is consistent with prior literature. [22, 52, 53, 54]

A possible limitation to face validity is that the patient focus group evaluating the 69 item questionnaire was not representative of the target population. Whereas the NHS Health Check programme is administered to both men and women and members of ethnic minorities, the focus group consisted only of white women. Furthermore, as these women had postgraduate education and worked in a health-related field, they may have had higher health literacy than the general population eligible for the NHS Health Check programme. Clarity, appropriateness, biases and presentation of information may have been differentially assessed by people with different levels of health literacy. A community based recruitment method aiming to recruit some of the hard to reach groups may have been more effective in getting a more representative patient focus group.

Additional studies should be conducted with larger samples to confirm the reliability and validity of the questionnaire. It would be useful to replicate the factor analytic process on an independent, larger sample to confirm the generalizability of these findings.[37]

CONCLUSIONS

The ABCD Risk Questionnaire showed evidence of satisfactory reliability and validity, is brief and easy to use. By capturing patients' views on CVD risk awareness during an NHS Health Check consultation, the questionnaire can be used to assess patients' understanding of CVD risk. Clinicians administering the questionnaire to patients may help to establish whether the programme is effective in empowering patients to make informed lifestyle choices about their health.

References

1. World Health Organisation. Prevention of Cardiovascular Disease - Guidelines for assessment and management of cardiovascular risk, 2007. http://www.who.int/cardiovascular_diseases/guidelines/Full%20text.pdf (accessed 08 March 2015)
2. Murray CJL, Richards MA, Newton JN, et al. UK health performance: findings of the Global Burden of Disease Study 2010. *The Lancet*. 2013; 381: 997-1020.
3. Barton GR, Goodall M, Bower P, Woolf S, Capewell S and Gabbay MB. Increasing heart-health lifestyles in deprived communities: economic evaluation of lay health trainers. *Journal of Evaluation in Clinical Practice*. 2012; 18: 835-40.
4. Yusuf S, Hawken S, Ôunpuu S, et al. Effect of potentially modifiable risk factors associated with myocardial infarction in 52 countries (the INTERHEART study): case-control study. *The Lancet*. 2004; 364: 937-52.
5. Bajekal M, Scholes S, Love H, et al. Analysing Recent Socioeconomic Trends in Coronary Heart Disease Mortality in England, 2000–2007: A Population Modelling Study. *PLoS Med*. 2012; 9: e1001237.
6. The National Institute for Health and Care Excellence (NICE). Services for the prevention of cardiovascular disease - NICE commissioning guides [CMG45], 2012.

For peer review only - http://bmjopen.bmj.com/site/about/guidelines.xhtml

ttps://www.nice.org.uk/guidance/cmg45/chapter/commissioning-services-for-the-prevention-of-cardiovascular-disease (accessed 12 June 2015)
7. Department of Health. Putting prevention first- vascular checks: risk assessment and management - next steps guidance for primary care trusts, 2009. http://www.dh.gov.uk/en/Publicationsandstatistics/Publications/PublicationsPolicyAndGuidance/DH_090277 (accessed 7 July 2016).
8. Public Health England. NHS Health Check Best Practice Guidance 2015, 2015. http://www.healthcheck.nhs.uk/commissioners_and_healthcare_professionals/national_guidance/ (accessed 6 March 2015)
9. Public Health England. NHS Health Check: Our Approach to the Evidence, 2013. https://www.gov.uk/government/uploads/system/uploads/attachment_data/file/224537/NHS_Health_Check_our_approach_to_the_evidence_v2.pdf;(accessed 12 June 2015).
10. Department of Health. Vascular Checks: Economic Modelling for Vascular Checks, 2008. http://www.dh.gov.uk/en/Publicationsandstatistics/Publications/PublicationsPolicyAndGuidance/DH_085869 (accessed 12 June 2015).
11. Collins GS and Altman DG. Predicting the 10 year risk of cardiovascular disease in the United Kingdom: independent and external validation of an updated version of QRISK2. *BMJ*. 2012; 344.
12. Homko CJ, Santamore WP, Zamora L, et al. Cardiovascular Disease Knowledge and Risk Perception Among Underserved Individuals at Increased Risk of Cardiovascular Disease. *Journal of Cardiovascular Nursing*. 2008; 23: 332-7
13. Webster R and Heeley E. Perceptions of risk: understanding cardiovascular disease. *Risk management and healthcare policy*. 2010; 3: 49-60.
14. Kirkland SA, MacLean DR, Langille DB, Joffres MR, MacPherson KM and Andreou P. Knowledge and awareness of risk factors for cardiovascular disease among Canadians 55 to 74 years of age: results from the Canadian Heart Health Surveys, 1986-1992. *CMAJ : Canadian Medical Association journal = journal de l'Association medicale canadienne*. 1999; 161: S10-6.
15. Potvin L, Richard L and Edwards AC. Knowledge of cardiovascular disease risk factors among the Canadian population: relationships with indicators of socioeconomic status. *CMAJ : Canadian Medical Association journal = journal de l'Association medicale canadienne*. 2000; 162: S5-11.
16. Sheridan SL, Viera AJ, Krantz MJ, et al. The Effect of Giving Global Coronary Risk Information to Adults: A Systematic Review. *Archives of Internal Medicine*. 2010; 170: 230-9.
17. Bergman HE, Reeve BB, Moser RP, Scholl S and Klein WMP. Development of a Comprehensive Heart Disease Knowledge Questionnaire. *American Journal of Health Education*. 2011; 42: 74-87.
18. Ammouri AA and Neuberger G. The Perception of Risk of Heart Disease Scale: development and psychometric analysis. *J Nurs Meas*. 2008; 16: 83-97.
19. Tovar EG, Rayens MK, Clark M and Nguyen H. Development and psychometric testing of the Health Beliefs Related to Cardiovascular Disease Scale: preliminary findings. *Journal of advanced nursing*. 2010; 66: 2772-84.
20. Strecher V.J. R, I.M. *The Health Belief Model. In Health Behavior and Health Education, Theory, Research and Practice (Glanz, K., Lewis, F.M., Rimer, B.K. eds)*. San Francisco: Jossey-Bass, 1997.
21. Prochaska JO, Redding, C.A., Evers, K.E. . *The Transtheoretical Model and Stages of Change. In Health Behavior and Health Education, Theory, Research and Practice (Glanz, K., Lewis, F.M., Rimer, B.K. eds).* . San Francisco: Jossey-Bass, 1997.
22. Hsu HY and Gallinagh R. The relationships between health beliefs and utilization of free health examinations in older people living in a community setting in Taiwan. *Journal of advanced nursing*. 2001; 35: 864-73.
23. Prochaska JO, DiClemente CC and Norcross JC. In search of how people change. Applications to addictive behaviors. *Am Psychol*. 1992; 47: 1102-14.

For peer review only - http://bmjopen.bmj.com/site/about/guidelines.xhtml

24. Prochaska JO, Norcross JC and DiClemente CC. *Changing for Good: A Revolutionary Six-Stage Program for Overcoming Bad Habits and Moving Your Life Positively Forward.* New York, NY Avon Books, 1994.

25. Department of Health. Physical Activity Guidelines for Adults (19-64 Years), 2011. http://www.nhs.uk/Livewell/fitness/Documents/adults-19-64-years.pdf (accessed 23 April 2015).

26. Department of Health. Improve Your NHS Health Check Numbers, 2014. http://www.nhs.uk/Conditions/nhs-health-check/Pages/Take-action-to-improve-your-health.aspx (accessed 23 April 2015).

27. Department of Health. Five a Day, 2013. http://www.nhs.uk/livewell/5aday/Pages/ADAYhome.aspx (accessed 23 April, 2015).

28. Health Do. Putting prevention first. NHS health check: Vascular risk assessment and management - best practice guidance. 2009.

29. Andrew S and Halcomb EJ. *Mixed Methods Research for Nursing and the Health Sciences*. Blackwell Publishing Ltd, 2009.

30. Chan CW. Perceptions of coronary heart disease: the development and psychometric testing of a measurement scale. *Psychol Health Med*. 2014; 19: 159-68.

31. Wells KJ, Arevalo M, Meade CD, et al. Development and validation of the biobanking attitudes and knowledge survey (BANKS). *Cancer Epidemiol Biomarkers Prev*. 2014; 23: 374-82.

32. Waltz CF, Strickland OL and Lenz ER. *Measurement in Nursing Research*. Second Edition ed. Philadelphia: F.A. Davis Company, 1991.

33. Waltz CF, Strickland OL and Lenz ER. *Measurement in Nursing and Health Research* Fourth Edition ed. New York: Springer Publishing Company, 2010.

34. Rust J and Golombok S. *Modern psychometrics: The science of psychological assessment.* Second Edition ed. London: Routledge, 1999.

35. JBS 3 Joint British Societies for the Prevention of Cardiovascular Disease. Lifetime Risk, 2014. http://www.jbs3risk.com/pages/lifetime_risk.htm (accessed 9 August 2016).

36. Nunnally JC and Bernstein IH. *Psychometric theory*. Third Edition ed. New York: McGraw-Hill Higher, INC, 1994.

37. DeVellis RF. *Scale Development Theory and Applications*. Third Edition ed. Los Angelos / London / New Delhi / Singapore / Washington DC: SAGE, 2012.

38. Aldridge V, Wade A and Koutoumanou E. Validity and Reliability University College London, Institute of Child Health2015.

39. Ferguson E and Cox T. Exploratory Factor Analysis: A Users'Guide. *International Journal of Selection and Assessment*. 1993; 1: 84-94.

40. Zwick WR and Velicer WF. Comparison of five rules for determining the number of components to retain. *Psychological Bulletin*. 1986; 99: 432-42.

41. Department for Communities and Local Government. The English Indices of Deprivation 2010 - Neighborhoods Statistical Release, 2011. https://www.gov.uk/government/uploads/system/uploads/attachment_data/file/6871/1871208.pdf (accessed 16 December 2014).

42. Krogsbøll Lasse T, Jørgensen Karsten J, Grønhøj Larsen C and Gøtzsche Peter C. General health checks in adults for reducing morbidity and mortality from disease. *Cochrane Database of Systematic Reviews*: (2012).

43. Capewell S and Graham H. Will cardiovascular disease prevention widen health inequalities? *PLoS Med*. 2010; 7: e1000320.

44. Chang KC-M, Soljak M, Lee JT, et al. Coverage of a national cardiovascular risk assessment and management programme (NHS Health Check): Retrospective database study. *Preventive Medicine*.

45. Artac M, Dalton ARH, Majeed A, Car J and Millett C. Effectiveness of a national cardiovascular disease risk assessment program (NHS Health Check): Results after one year. *Preventive Medicine*. 2013.

For peer review only - http://bmjopen.bmj.com/site/about/guidelines.xhtml

46. Artac M, Dalton ARH, Majeed A, Car J, Huckvale K and Millett C. Uptake of the NHS Health Check programme in an urban setting. *Family Practice*. 2013; 30: 426-35.
47. Dalton ARH, Bottle A, Okoro C, Majeed A and Millett C. Uptake of the NHS Health Checks programme in a deprived, culturally diverse setting: cross-sectional study. *J Public Health (Oxf)*. 2011; 33: 422-9.
48. Chang KC, Lee JT, Vamos EP, et al. Impact of the National Health Service Health Check on cardiovascular disease risk: a difference-in-differences matching analysis. *CMAJ : Canadian Medical Association journal = journal de l'Association medicale canadienne*. 2016.
49. Robson J, Dostal I, Sheikh A, et al. The NHS Health Check in England: an evaluation of the first 4 years. *BMJ Open*. 2016; 6: e008840.
50. NHS Choices. Eating a Balanced Diet. 2016. http://www.nhs.uk/Livewell/Goodfood/Pages/Healthyeating.aspx (accessed 27 April 2017).
51. Public Health England. NHS Health Check Best practice guidance. 2017. http://www.healthcheck.nhs.uk/commissioners_and_providers/guidance/national_guidance1/ (accessed 27 April 2017).
52. Grayland J and Wilson R. Improving male life expectancy in Birmingham. *Public Health*. 2009; 123: e50-e6.
53. Hunt BD, Hiles SL, Chauhan A, et al. Evaluation of the Healthy LifeCheck programme: a vascular risk assessment service for community pharmacies in Leicester city, UK. *J Public Health (Oxf)*. 2013; 35: 440-6.
54. Visram S, Carr SM and Geddes L. Can lay health trainers increase uptake of NHS health checks in hard-to-reach populations? A mixed-method pilot evaluation. *J Public Health (Oxf)*. 2014.

For peer review only

For peer review only - http://bmjopen.bmj.com/site/about/guidelines.xhtml

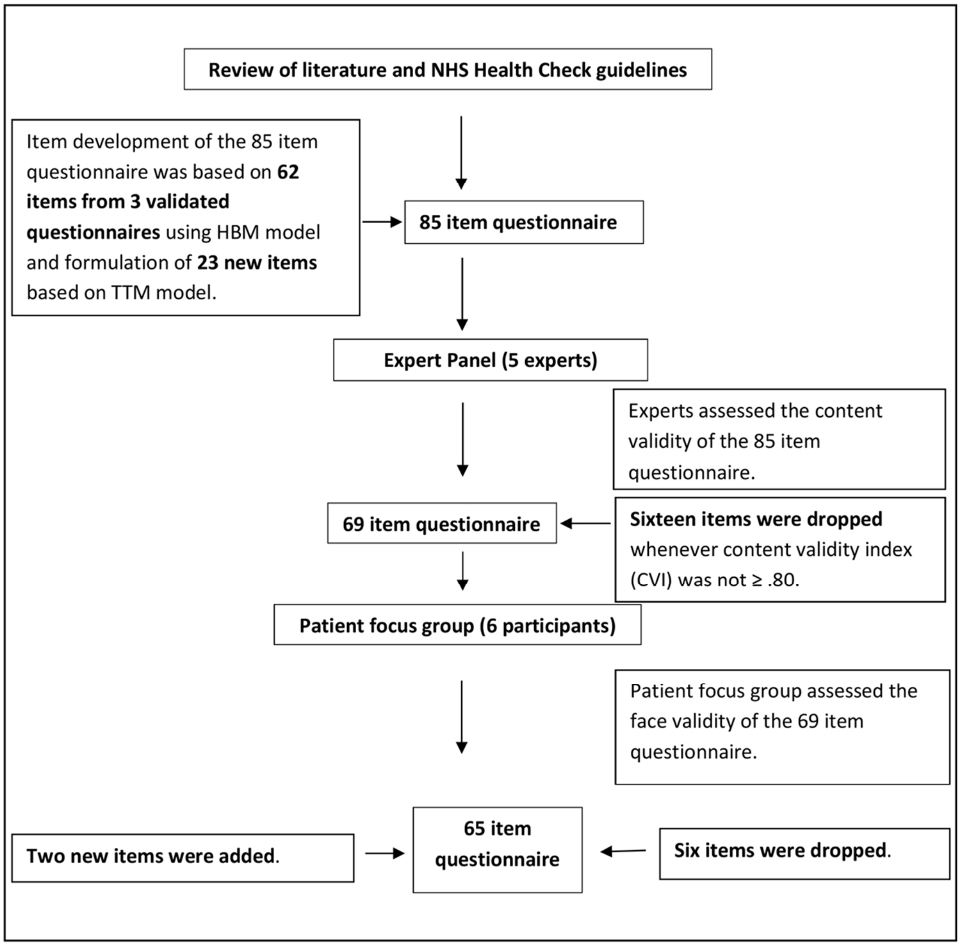

Figure 1

112x112mm (300 x 300 DPI)

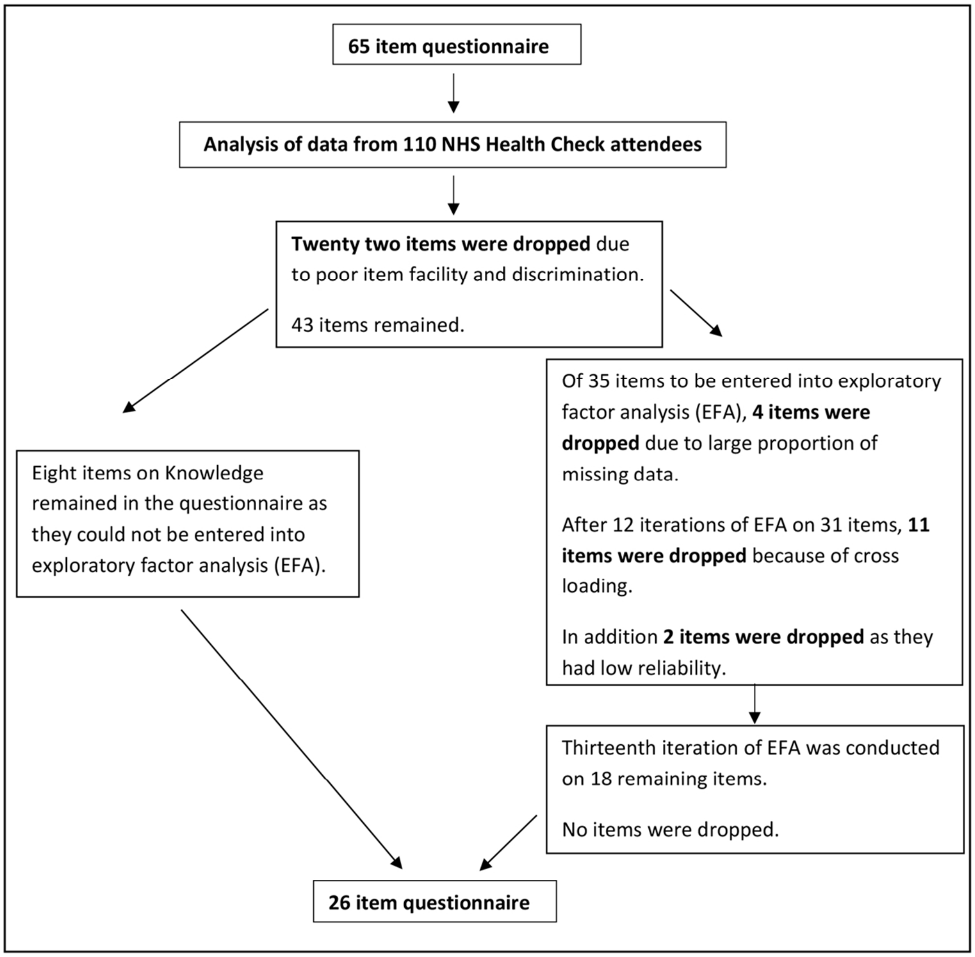

Figure 2

112x112mm (300 x 300 DPI)

Appendix A. 85 Item Questionnaire

Subscale	Items	Answers
Knowledge of CVD Risk and Prevention	1. Eating a lot of red meat increases heart attack and stroke risk.	True, False, Don't Know<br>T=True F=False<br>Correct Answers<br>Q1=T Q2=F Q3=T Q4=F Q5=T Q6=F Q7=F Q8=T Q9=T Q10=T Q11=F Q12=T Q13=T Q14=T Q15=T Q16=F Q17=T Q18=F
	2. Most people can tell whether or not they have high blood pressure.	
	3. You can reduce your risk of heart attack or stroke by being physically active.	
	4. 'High' blood pressure is defined as 110/80 (systolic/diastolic) or higher.	
	5. Dietary fibre lowers blood cholesterol.	
	6. The most important cause of heart attack and stroke is stress.	
	7. Trans-fats are healthier for the heart than most other kinds of fats.	
	8. Walking and gardening are considered types of exercise that can lower the risk of having a heart attack or stroke.	
	9. You can reduce your chance of developing a heart attack or stroke by eating five-a-day diet of fruits and vegetables.	
	10. Moderate physical activity of 150 minutes a week will reduce your chances of developing a heart attack or stroke.	
	11. People who quit smoking by 60 add five years to their life.	
	12. People who have diabetes are at higher risk having a heart attack or stroke.	
	13. Managing your stress levels will help you to manage your blood pressure.	
	14. HDL refers to 'good' cholesterol, and LDL refers to 'bad' cholesterol.	
	15. The healthiest exercise for the heart involves rapid breathing for a sustained period of time.	
	16. Many vegetables are high in cholesterol.	
	17. You are more likely to have a heart attack or stroke if you're overweight or obese.	
	18. Drinking alcohol has nothing to do with reducing the risk of heart attack or stroke.	
Perceived Risk and Vulnerability of CVD	19. There is a possibility that I will have a heart attack or stroke.	1 = Strongly disagree; 2 = disagree; 3 = agree; 4 = strongly agree
	20. There is a good chance I will experience a heart attack or stroke during the next 10 years.	
	21. A person who gets a heart attack or stroke has no chance of recovering.	
	22. I have a high chance of getting a heart attack or stroke because of my past behaviours.	
	23. I feel sure that I will have a heart attack or stroke.	
	24. Healthy lifestyle habits are unattainable.	
	25. It is likely that I will get a heart attack or stroke.	
	26. I am at risk for having a heart attack or stroke.	
	27. It is possible that I will have a heart attack or stroke.	
	28. I am not doing anything now that is unhealthy to my heart.	
	29. I am too young to have a heart attack or stroke.	
	30. People like me do not get a heart attack or stroke.	
	31. I am very healthy so I will not have a heart attack or stroke.	
	32. I am not worried that I might have a heart attack or stroke.	
	33. People my age are too young to have a heart attack or stroke.	

For peer review only

For peer review only - http://bmjopen.bmj.com/site/about/guidelines.xhtml

	34. People my age do not have a heart attack or stroke.	
	35. My lifestyle habits do not put me at risk for having a heart attack or stroke.	
	36. No matter what I do, if I am going to have a heart attack or stroke, I will have one.	
	37. People who do not have a heart attack or stroke are just plain lucky.	
	38. The causes of a heart attack or stroke are unknown.	
Perceived Susceptibility	39. It is likely that I will suffer from a heart attack or stroke in the future.	
	40. My chances of suffering from a heart attack or stroke in the next few years are great.	
	41. Having a heart attack or stroke is currently a possibility for me.	
	42. I feel I will suffer from a heart attack or stroke sometime during my life.	
	43. I am concerned about the likelihood of having a heart attack or stroke in the near future.	
Perceived Severity	44. Heart attacks and strokes are always fatal.	
	45. Having a heart attack or stroke will threaten my relationship with my significant other.	
	46. My whole life would change if I had a heart attack or stroke.	
	47. Having a heart attack or stroke would have a very bad effect on my sex life.	
	48. If I have a heart attack or stroke I will die within 10 years.	
Perceived Benefits	49. Increasing my exercise will decrease my chances of having a heart attack or stroke.	
	50. Eating a healthy diet will decrease my chance of having a heart attack or stroke.	
	51. Stopping smoking will reduce my chance of having a heart attack or stroke.	
	52. When I exercise I am doing something good for myself.	
	53. When I eat healthy I am doing something good for myself.	
	54. Cutting down on alcohol will decrease my chances of having a heart attack or stroke.	
Perceived Barriers	55. I do not know appropriate exercises to perform to reduce my risk of developing cardiovascular disease.	
	56. I do not know the recommended drinking limits for men or women.	
	57. I do not have time to exercise for 30 minutes a day on most days of the week.	
	58. I do not know what is considered a healthy diet that would prevent me from developing cardiovascular disease.	
	59. I will not have energy if I stop smoking.	
	60. I cannot afford to buy healthy foods.	
	61. I have other problems more important than worrying about diet and exercise.	
Self Efficacy	62. How confident are you that you know or can control the risks of having a heart attack or stroke?	1= not at all confident, 2=somewhat confident, 3= moderately confident, 4=very confident,
	63. How confident are you that you know or can maintain a healthy weight by exercising regularly?	
	64. How confident are you that you know or can stop smoking if you want to?	
	65. How confident are you that you know or can consume less alcohol?	

For peer review only

For peer review only - http://bmjopen.bmj.com/site/about/guidelines.xhtml

	66. How confident are you that you know or can control your blood pressure and/or cholesterol levels by taking your prescribed medications?	5=completely confident
	67. How confident are you that you know or can eat a healthy and balanced diet?	
Intention to Change Behaviour or Cues to Action	68. I want to stop smoking (if you do smoke).	1 = Strongly disagree; 2 = disagree; 3 = agree; 4 = strongly agree
	69. I intend to maintain a healthy weight.	
	70. I intend to be physically active within two months.	
	71. I expect to maintain a healthy weight.	
	72. I want to be physically active.	
	73. I intend to eat a healthy and balanced diet within two months.	
	74. I expect to stop smoking (if you do smoke).	
	75. I want to cut down on alcohol.	
	76. I want to maintain a healthy and balanced diet.	
	77. I intend to stop smoking within two months (if you do smoke).	
	78. I expect to eat a healthy and balanced diet.	
	79. I intend to cut down on alcohol in the next two months.	
	80. I expect to be physically active.	
	81. I expect to cut down on alcohol.	
	82. I want to eat a healthy and balanced diet.	
	83. I expect to take my medication to control my blood pressure and/or cholesterol	
	84. I want to take my medication to control my blood pressure and/or cholesterol	
	85. I intend to take my medication to control my blood pressure and/or cholesterol within two months	

For peer review only - http://bmjopen.bmj.com/site/about/guidelines.xhtml

Appendix B. 65 Item Questionnaire

Scale	Subscale	Items	Coding of Answers
**Knowledge**<br><br>(15 items)<br><br>Higher sum score = more knowledgeable / more correct	**CVD Risk Knowledge – Risk of having a heart attack / stroke**<br><br>(15 items)	1. Eating a lot of red meat increases heart attack and stroke risk.	Correct Answers<br><br>Q1-T Q6-T Q11-F<br>Q2-F Q7-T Q12-T<br>Q3-T Q8-T Q13-T<br>Q4-T Q9-T Q14-T<br>Q5-T Q10-T Q15-F<br><br>T= True<br>F= False<br><br>Correct: Score = 1, Incorrect or Don't Know Score = 0.
		2. Most people can tell whether or not they have high blood pressure.	
		3. You can reduce your risk of heart attack or stroke by being physically active.	
		4. One of the main causes of heart attack and stroke is stress.	
		5. Walking and gardening are considered types of exercise that can lower the risk of having a heart attack or stroke.	
		6. You can reduce your chance of developing a heart attack or stroke by eating at least five portions of fruit and vegetables a day.	
		7. Moderate intensity activity of 2 ½ hours a week will reduce your chances of developing a heart attack or stroke.	
		8. People who have diabetes are at higher risk of having a heart attack or stroke.	
		9. Managing your stress levels will help you to manage your blood pressure.	
		10. The healthiest exercise for the heart involves rapid breathing for 10 minutes or more.	
		11. Many vegetables are high in cholesterol.	
		12. You are more likely to have a heart attack or stroke if you're overweight or obese.	
		13. Drinking high levels of alcohol can increase your cholesterol and triglyceride levels.	
		14. HDL refers to 'good' cholesterol, and LDL refers to 'bad' cholesterol.	
		15. Family history of heart disease is not a risk	

For peer review only - http://bmjopen.bmj.com/site/about/guidelines.xhtml

		factor for high blood pressure.	
**Perceived CVD Risk** (15 items)<br><br>Composite score = sum across subscales. Higher score = higher perception of risk of having a heart attack or stroke	**Dread Risk** (7 items)	16. There is a possibility that I will have a heart attack or stroke.	Higher sum score = Higher perceived lack of control, dread, catastrophic potential and fatal consequences<br><br>1 = Strongly disagree; 2 = disagree; 3 = agree; 4 = strongly agree; N/A = 0
		17. There is a good chance I will experience a heart attack or stroke in the next 10 years.	
		18. It is likely I will have a heart attack or stroke because of my past and/or present behaviours.	
		19. I feel sure that I will have a heart attack or stroke.	
		20. It is likely that I will have a heart attack or stroke some time during my life.	
		21. I am at risk for having a heart attack or stroke some time during my life.	
		22. It is possible that I will have a heart attack or stroke within the next 10 years.	
	**Risk** (3 items)	23. I am too young to have a heart attack or stroke.	Higher sum score = Higher perceived hazards that has few, moderate, known outcomes and consequences<br><br>**Reverse coded**<br>4 = Strongly disagree; 3 = disagree; 2 = agree; 1 = strongly agree; N/A = 0
		24. People like me do not have a heart attack or stroke.	
		25. I am not worried that I might have a heart attack or stroke.	
	**Unknown Risk** (5 items)	26. I am not doing anything now that is unhealthy to my heart.	Higher sum score = Higher perceived hazards judged to be unobservable, unknown, new, and delayed in their manifestation of harm<br><br>**Reverse coded**<br>4 = Strongly disagree; 3 = disagree; 2 = agree; 1 = strongly agree; N/A = 0
		27. I am very healthy so I will not have a heart attack or stroke.	
		28. My lifestyle habits do not put me at risk for having a heart attack or stroke.	
		29. No matter what I do, if I am going to have a heart attack or stroke, I will have one.	
		30. People who do not have a heart attack or stroke are just plain lucky.	

For peer review only - http://bmjopen.bmj.com/site/about/guidelines.xhtml

**CVD Health Beliefs** (13 items)	**Susceptibility** (4 items)	31. It is likely that I will suffer from a heart attack or stroke in the future.	Higher average score = Higher perceived personal risk of heart attack and stroke<br><br>1 = Strongly disagree; 2 = disagree; 3 = agree; 4 = strongly agree; N/A = 0
		32. My chances of suffering from a heart attack or stroke in the next 10 years are great.	
		33. I feel I will suffer from a heart attack or stroke sometime during my life.	
		34. I am concerned about the likelihood of having a heart attack or stroke in the near future.	
	**Severity** (3 items)	35. Heart attacks and strokes are always fatal.	Higher average score = Higher perceived severity of heart attack and stroke<br><br>1 = Strongly disagree; 2 = disagree; 3 = agree; 4 = strongly agree; N/A = 0
		36. My whole life would change if I had a heart attack or stroke.	
		37. If I have a heart attack or stroke I will die within 10 years.	
	**Benefits** (4 items)	38. Eating at least five portions of fruit and vegetables a day will decrease my chances of having a heart attack or stroke.	Higher average score = Higher perceived benefits of diet, exercise, consuming less alcohol and smoking cessation for reducing risk for heart attack and stroke<br><br>**Reverse coded**<br>4 = Strongly disagree; 3 = disagree; 2 = agree; 1 = strongly agree; N/A = 0
		39. Increasing my exercise to at least 2 ½ hours a week will decrease my chances of having a heart attack or stroke.	
		40. When I exercise for at least 2 ½ hours a week I am doing something good for the health of my heart.	
		41. When I eat at least five portions of fruit and vegetables a day I am doing something good for the health of my heart.	
	**Barriers** (2 items)	42. I do not have time to exercise on most days of the week.	Higher average score = Higher perception of select barriers to engaging in heart attack and stroke risk reducing behaviours<br><br>1 = Strongly disagree; 2 = disagree; 3 = agree; 4 = strongly agree; N/A = 0
		43. I cannot afford to buy healthy foods.	
**Self**	**CVD risk**	How confident are you that you know how to or can…	Higher average score = higher perceived

For peer review only - http://bmjopen.bmj.com/site/about/guidelines.xhtml

**Efficacy** (5 items)	**reduction self efficacy** (5 items)	44. Control the risks of having a heart attack or stroke.	confidence 1 = Not at all confident; 2 = somewhat confident; 3 = very confident; 4 = completely confident; N/A = 0
		45. Maintain a healthy weight by exercising at least 2½ hours a week within the next two months.	
		46. Stop smoking if you want to.	
		47. Drink within the recommended levels of alcohol.	
		48. Eat at least five portions of fruit and vegetables per day within the next two months.	
**Intention / Readiness to Change** (17 items)	**Exercise** (4 items)	49. I am not thinking about exercising for 2 ½ hours a week.	Higher average score = Higher perceived readiness for change with regard to exercise behaviour 1 = Strongly disagree; 2 = disagree; 3 = agree; 4 = strongly agree; N/A = 0
		50. I am thinking about exercising at least 2 ½ hours a week.	
		51. I intend or want to exercise at least 2 ½ hours a week.	
		52. I am ready or have started to exercise 2 ½ hours a week.	
	**Diet** (4 items)	53. I am not thinking about eating at least five portions of fruit and vegetables a day.	Higher average score = Higher perceived readiness for change with regard to health dietary behaviour 1 = Strongly disagree; 2 = disagree; 3 = agree; 4 = strongly agree; N/A = 0
		54. I am thinking about eating at least five portions of fruit and vegetables a day.	
		55. I intend or want to eat at least five portions of fruit and vegetables a day.	
		56. I am ready or started to eat at least five portions of fruit and vegetables a day.	
	**Alcohol** (4 items)	57. I am thinking about cutting down on alcohol.	Higher average score = Higher perceived readiness for change with regard to alcohol consumption behaviour 1 = Strongly disagree; 2 = disagree; 3 = agree; 4 = strongly agree; N/A = 0
		58. I intend or want to cut down on alcohol.	
		59. I have been cutting down on alcohol.	
		60. I am not thinking about cutting down on alcohol.	
	**Smoking** (5 items)	61. I am thinking of stopping smoking within two months.	Higher average score = Higher perceived readiness for change with regard to smoking cessation

For peer review only - http://bmjopen.bmj.com/site/about/guidelines.xhtml

		62. I have reduced or stopped smoking.	behaviour<br><br>1 = Strongly disagree; 2 = disagree; 3 = agree; 4 = strongly agree; N/A = 0
		63. I intend or want to stop smoking.	
		64. If I stop smoking it will reduce my chances of having a heart attack or stroke.	
		65. I am not thinking about stopping smoking.	

For peer review only

For peer review only - http://bmjopen.bmj.com/site/about/guidelines.xhtml

Appendix C. Population characteristics of 110 NHS Health Check attendees

Population Characteristics		n	% Total
Gender	Male	51	46.4%
	Female	56	50.9%
Age group	40-49	45	40.9%
	50-59	40	36.4%
	60-74	14	12.7%
Ethnicity	White	93	84.5%
	Mixed	2	1.8%
	Asian	2	1.8%
	Black	4	3.6%
	Other	4	3.6%
*Deprivation**	IMD 1 - least deprived	14	12.7%
	IMD 2	30	27.3%
	IMD 3	12	10.9%
	IMD 4	31	28.2%
	IMD 5 - most deprived	14	12.7%
Cholesterol	Raised total cholesterol TC≥ 5 mmol/l	66	60.0%
Blood pressure	High blood pressure BP ≥ 140/90 mm Hg	28	25.5%
Body Mass Index (BMI)	Obese (BMI>=30)	26	23.6%
Physical activity	Physically inactive	22	20.0%
Smoking status	Smokers	20	18.2%
Alcohol consumption	Excessive drinkers	13	11.8%
*10 year predicted risk of CVD***	High CVD Risk (QRisk2>=20%)	5	4.5%
	Medium CVD Risk (10%=<Qrisk2<20%)	21	19.1%
	Low CVD Risk (QRisk2<10%)	85	77.3%
Mean Values & 95% Confidence Intervals	Age (95% CI)	51.52	(49.93, 53.12)
	Total Cholesterol (95% CI)	5.42	(5.19, 5.64)
	HDL Cholesterol (95% CI)	1.44	(1.36, 1.53)
	Cholesterol Ratio (TC/HDL) (95% CI)	4.12	(3.73, 4.52)
	SBP (95% CI)	129.60	(125.76, 133.44)
	DBP (95% CI)	81.63	(79.62, 83.63)
	BMI (95% CI)	27.24	(26.17, 28.31)
	Q-Risk 2 (95% CI)	6.27	(5.19, 7.34)

*Deprivation was measured using the Index of Multiple Deprivation (IMD).

**Ten year predicted risk of CVD was estimated using the Q-Risk 2 algorithm[1]

Notes: SBP = systolic blood pressure; DBP = diastolic blood pressure; CI = confidence interval; HDL = high density lipoprotein; CVD = cardiovascular disease

References

1. Hippisley-Cox J, Coupland C, Vinogradova Y, et al. Predicting cardiovascular risk in England and Wales: prospective derivation and validation of QRISK2. *BMJ*. 2008; 336.

For peer review only - http://bmjopen.bmj.com/site/about/guidelines.xhtml

Appendix D. The ABCD Risk Questionnaire and scoring guide

Scale	Items	Coding
**Knowledge**<br><br>Higher sum score = more knowledgeable / more correct about having a heart attack or stroke	1. One of the main causes of heart attack and stroke is stress.<br><br>2. Walking and gardening are considered types of exercise that can lower the risk of having a heart attack or stroke.<br><br>3. Moderately intense activity of 2 ½ hours a week will reduce your chances of having a heart attack or stroke.<br><br>4. People who have diabetes are at higher risk of having a heart attack or stroke.<br><br>5. Managing your stress levels will help you to manage your blood pressure.<br><br>6. Drinking high levels of alcohol can increase your cholesterol and triglyceride levels.<br><br>7. HDL refers to 'good' cholesterol, and LDL refers to 'bad' cholesterol.<br><br>8. A family history of heart disease is not a risk factor for high blood pressure.	Correct Answers:<br><br>Q1-T<br><br>Q2-T<br><br>Q3-T<br><br>Q4-T<br><br>Q5-T<br><br>Q6-T<br><br>Q7-T<br><br>Q8-F<br><br>T= True<br>F= False<br><br>Correct: Score = 1,<br>Incorrect or Don't Know: Score = 0.
**Perceived Risk of**<br><br>**Heart**<br><br>**Attack/Stroke**<br><br>Higher sum score = higher perception of risk of having a heart attack or stroke	9. I feel I will suffer from a heart attack or stroke sometime during my life.	1=Strongly disagree; 2 = disagree; 3 = agree; 4 = strongly agree; N/A = 0
	10. It is likely that I will suffer from a heart attack or stroke in the future.	1=Strongly disagree; 2 = disagree; 3 = agree; 4 = strongly agree; N/A = 0
	11. It is likely that I will have a heart attack or stroke some time during my life.	1=Strongly disagree; 2 = disagree; 3 = agree; 4 = strongly agree; N/A = 0
	12. There is a good chance I will experience a heart attack or stroke in the next 10 years.	1=Strongly disagree; 2 = disagree; 3 = agree; 4 = strongly agree; N/A = 0
	13. My chances of suffering from a heart attack or stroke in the next 10 years are great.	1=Strongly disagree; 2 = disagree; 3 = agree; 4 = strongly agree; N/A = 0
**Perceived Risk of**	14. It is likely I will have a heart attack or stroke because of my past and/or present behaviours.	1=Strongly disagree; 2 = disagree; 3 = agree; 4 = strongly agree; N/A = 0

For peer review only

For peer review only - http://bmjopen.bmj.com/site/about/guidelines.xhtml

Scale	Items	Coding
**Heart Attack/Stroke**	15. I am not worried that I might have a heart attack or stroke.	**Reverse coded** 4=Strongly disagree; 3 = disagree; 2 = agree; 1 = strongly agree; N/A = 0
	16. I am concerned about the likelihood of having a heart attack or stroke in the near future.	1=Strongly disagree; 2 = disagree; 3 = agree; 4 = strongly agree; N/A = 0
**Perceived Benefits and Intentions to Change** Higher average score = Higher perceived benefits of diet and exercise and higher perceived readiness for change in regards to exercise behaviour	17. I am thinking about exercising at least 2½ hours a week.	1=Strongly disagree; 2 = disagree; 3 = agree; 4 = strongly agree; N/A = 0
	18. I intend or want to exercise at least 2½ hours a week.	1=Strongly disagree; 2 = disagree; 3 = agree; 4 = strongly agree; N/A = 0
	19. When I exercise for at least 2½ hours a week I am doing something good for the health of my heart.	1=Strongly disagree; 2 = disagree; 3 = agree; 4 = strongly agree; N/A = 0
	20. I am confident that I can maintain a healthy weight by exercising at least 2½ hours a week within the next two months.	1=Strongly disagree; 2 = disagree; 3 = agree; 4 = strongly agree; N/A = 0
	21. I am not thinking about exercising for 2 ½ hours a week.	**Reverse coded** 4=Strongly disagree; 3 = disagree; 2 = agree; 1 = strongly agree; N/A = 0
	22. When I eat at least five portions of fruit and vegetables a day I am doing something good for the health of my heart.	1=Strongly disagree; 2 = disagree; 3 = agree; 4 = strongly agree; N/A = 0
	23. Increasing my exercise to at least 2½ hours a week will decrease my chances of having a heart attack or stroke.	1=Strongly disagree; 2 = disagree; 3 = agree; 4 = strongly agree; N/A = 0
**Healthy Eating Intentions** Higher average score = Higher perceived readiness for change with regard to health dietary behaviour	24. I am confident that I can eat at least five portions of fruit and vegetables per day within the next two months.	1=Strongly disagree; 2 = disagree; 3 = agree; 4 = strongly agree; N/A = 0
	25. I am thinking about eating at least five portions of fruit and vegetables a day.	1=Strongly disagree; 2 = disagree; 3 = agree; 4 = strongly agree; N/A = 0
	26. I am not thinking about eating at least five portions of fruit and vegetables a day.	**Reverse coded** 4=Strongly disagree; 3 = disagree; 2 = agree; 1 = strongly agree; N/A = 0

The Attitudes and Beliefs about Cardiovascular Disease (ABCD) Risk Questionnaire is licensed under the Creative Commons Attribution-NonCommercial-NoDerivatives 4.0 International License

For peer review only - http://bmjopen.bmj.com/site/about/guidelines.xhtml

Appendix E. Correlations of Factors of the ABCD Risk Questionnaire with Predicted CVD Risk using Spearman's Rho among 110 NHS Health Check Attendees

		KNOWLEDGE	PERCEIVED RISK	PERCEIVED BENEFITS	HEALTHY INTENTIONS	IMD2010_Quintile	BMI	QRISK2	QRISK2_L_M_H
KNOWLEDGE	Correlation Coefficient	1.000	-.124	-.148	-.106	-.002	-.225*	-.007	-.063
	Sig. (2-tailed)	.	.236	.175	.319	.986	.021	.941	.522
	N	107	93	86	91	99	105	104	104
PERCEIVED RISK	Correlation Coefficient	-.124	1.000	-.195	-.188	.239*	.389**	.220*	.173
	Sig. (2-tailed)	.236	.	.080	.088	.025	.000	.036	.102
	N	93	95	82	84	87	92	91	91
PERCEIVED BENEFITS	Correlation Coefficient	-.148	-.195	1.000	.533**	-.287**	-.068	-.118	-.232*
	Sig. (2-tailed)	.175	.080	.	.000	.009	.538	.284	.033
	N	86	82	88	83	81	85	84	84
HEALTHY INTENTIONS	Correlation Coefficient	-.106	-.188	.533**	1.000	-.261*	.084	-.072	-.116
	Sig. (2-tailed)	.319	.088	.000	.	.016	.430	.504	.279
	N	91	84	83	93	85	90	89	89

For peer review only - http://bmjopen.bmj.com/site/about/guidelines.xhtml

		KNOWLEDGE	PERCEIVED RISK	PERCEIVED BENEFITS	HEALTHY INTENTIONS	IMD2010_Quintile	BMI	QRISK2	QRISK2_L_M_H
IMD2010_Quintile	Correlation Coefficient	-.002	.239*	-.287**	-.261*	1.000	-.008	.009	.017
	Sig. (2-tailed)	.986	.025	.009	.016	.	.938	.931	.870
	N	99	87	81	85	101	101	100	100
BMI	Correlation Coefficient	-.225*	.389**	-.068	.084	-.008	1.000	.020	.028
	Sig. (2-tailed)	.021	.000	.538	.430	.938	.	.839	.777
	N	105	92	85	90	101	107	106	106
QRISK2	Correlation Coefficient	-.007	.220*	-.118	-.072	.009	.020	1.000	.694**
	Sig. (2-tailed)	.941	.036	.284	.504	.931	.839	.	.000
	N	104	91	84	89	100	106	106	106
QRISK2_L_M_H	Correlation Coefficient	-.063	.173	-.232*	-.116	.017	.028	.694**	1.000
	Sig. (2-tailed)	.522	.102	.033	.279	.870	.777	.000	.
	N	104	91	84	89	100	106	106	106

* Correlation is significant at the 0.05 level (2-tailed). ** Correlation is significant at the 0.01 level (2-tailed).

QRisk2_L_M_H categorizes predicted CVD risk from Low CVD Risk (QRISK2<10%), to Medium CVD Risk (10%<=QRISK2<20%), to High CVD Risk (QRISK2>=20%).

IMD2010Quintile categorizes deprivation from 1=least deprived to 5=most deprived.

BMJ Open

Development of a questionnaire to evaluate patients' awareness of cardiovascular disease risk in England's National Health Service Health Check preventive cardiovascular programme

Journal:	*BMJ Open*
Manuscript ID	bmjopen-2016-014413.R3
Article Type:	Research
Date Submitted by the Author:	01-Aug-2017

Complete List of Authors:	Woringer, Maria; Imperial College London Department of Primary Care and Public Health, 3rd Floor Reynolds Building Charing Cross Campus St Dunstans Road, W6 8RP<br>Nielsen, Jessica; City University London, School of Arts and Social Sciences, Department of Psychology, City University London Northampton Square, EC1V 0HB<br>Zibarras, Lara; City University London, School of Arts and Social Sciences, Department of Psychology, City University London Northampton Square, EC1V 0HB<br>Evason, Julie; Health Diagnostics Ltd, Chatham House, Dee Hills Park, CH3 5AR<br>Kassianos, Angelos; University College London, Department of Applied Health Research<br>Harris, Matthew; Imperial College London, Institute of Global Health Innovation, Level 1, Faculty Building South Kensington Campus, SW7 2NA ; Imperial College London Department of Primary Care and Public Health, 3rd Floor Reynolds Building Charing Cross Campus St Dunstans Road, W6 8RP<br>Majeed, Azeem; Imperial College London, Primary Care & Public Health, 3rd Floor Reynolds Building Charing Cross Campus St Dunstans Road, W6 8RP<br>Soljak, Michael; Imperial College London, Primary Care & Public Health, 3rd Floor Reynolds Building Charing Cross Campus St Dunstans Road, W6 8RP

**Primary Subject Heading**:	Communication
Secondary Subject Heading:	General practice / Family practice, Health services research
Keywords:	QUALITATIVE RESEARCH, PREVENTIVE MEDICINE, PRIMARY CARE, PUBLIC HEALTH

SCHOLARONE™
Manuscripts

For peer review only

Development of a questionnaire to evaluate patients' awareness of cardiovascular disease risk in England's National Health Service Health Check preventive cardiovascular programme

Author Names and Affiliations:

Ms. Maria Woringer (a)

Dr. Jessica Jones Nielsen (b)

Dr. Lara Zibarras (b)

Ms. Julie Evason (c)

Dr. Angelos P. Kassianos (d)

Dr. Matthew Harris (e)

Professor Azeem Majeed (a)

Dr. Michael Soljak (a)

Affiliations :

a) Department of Primary Care and Public Health, Imperial College London

b) Department of Psychology, City University London

c) Health Diagnostics Ltd, Chester, UK

d) Department of Applied Health Research, University College London

e) Institute of Global Health Innovation, Imperial College London

Word Count (excluding title page, declarations, abstract, keywords, strengths and limitations and references): 3,998

Author responsible for correspondences concerning the manuscript:

Maria Woringer; Imperial College London, Department of Primary Care and Public Health, Charing Cross Campus, The Reynolds Building, St Dunstan's Road, London W6 8RP, UK m.woringer@imperial.ac.uk; Tel: +44(0)2075940789

For peer review only

List of Declarations:

Dissemination of Study Findings

The work on the development and validation of the patient questionnaire was presented as a poster titled “Development and Validation of the Attitudes and Beliefs about Cardiovascular Disease (ABCD) Risk Survey” at the NHS Health Check 2015 – Improvement through Collaboration conference in Leeds, England on 26 February, 2015. In addition, an abstract titled “Development and validation of a patient survey to assess the effectiveness of cardiovascular disease screening” was selected for oral presentation at the First International Conference of Public Health, Primary Care and Congress of Person Centred Medicine on October 29, 2015 and accepted for publication in the International Journal of Person Centred Medicine. This submission is not under consideration by any other journal. All authors have approved the manuscript and this submission.

Permissions

Favourable ethical opinion for the study - “Patient Evaluation of the NHS Health Check Programme to Investigate the Programme’s Effectiveness in Communicating CVD Risk” was obtained from the NRES Committee London – City & East reference number 13/LO/1885.

Study participants gave their written informed consent to participate in the study and to share their results and medical data.

The Attitudes and Beliefs about Cardiovascular Disease (ABCD) Risk Questionnaire is licensed under the Creative Commons Attribution-NonCommercial-NoDerivatives 4.0 International License.

Funding

This work was supported by the National Institute for Health Research (NIHR) Diagnostics Evidence Co-operatives (DEC) / Collaboration for Leadership in Applied Health Research and Care (CLAHRC) grant.

Data Sharing Agreement

No additional data available.

Competing Interests

None.

Contributors

MW, AM, MS, and HW designed the study, JE supplied the data. JJN designed the validation instrument, LZ performed the psychometric analysis. JE, AK, MH and AM reviewed the validation instrument’s face and content validity. All authors discussed data analyses and interpreted the results. MW wrote the first draft of the manuscript. All authors critically revised and approved the final manuscript. MW had full access to all the data used in the study and takes responsibility for the integrity of the data and the accuracy of the data analysis. MW is the guarantor.

For peer review only - http://bmjopen.bmj.com/site/about/guidelines.xhtml

For peer review only

ABSTRACT

Background

The National Health Service (NHS) Health Check is a CVD risk assessment and management programme in England aiming to increase CVD risk awareness among people at increased risk of CVD. There is no tool to assess the effectiveness of the programme in communicating CVD risk to patients.

Aims

The aim of this paper was to develop a questionnaire examining patients' CVD risk awareness for use in health service research evaluations of the NHS Health Check programme.

Methods

We developed an 85 item questionnaire to determine patients' views of their risk of CVD. The questionnaire was based on a review of the relevant literature. After review by an expert panel and focus group discussion, 22 items were dropped and 2 new items were added. The resulting 65 item questionnaire with satisfactory content validity (content validity indices >=0.80) and face validity was tested on 110 NHS Health Check attendees in primary care in a cross sectional study between May 21 and July 28, 2014.

Results

Following analyses of data, we reduced the questionnaire from 65 to 26 items. The 26 item questionnaire constitutes 4 scales: Knowledge of CVD Risk and Prevention, Perceived Risk of Heart Attack/Stroke, Perceived Benefits and Intention to Change Behaviour and Healthy Eating Intentions. Perceived Risk (Cronbach's α = 0.85) and Perceived Benefits and Intention to Change Behaviour (Cronbach's α = 0.82) have satisfactory reliability (Cronbach's α >=0.70). Healthy Eating Intentions (Cronbach's α = 0.56) is below minimum threshold for reliability but acceptable for a three item scale.

Conclusions

The resulting questionnaire, with satisfactory reliability and validity, may be used in assessing patients' awareness of CVD risk among NHS Health Check attendees.

Word Count: 275

Keywords: cardiovascular disease, primary prevention, risk assessment, questionnaire

Strengths and limitations of this study

- Questionnaire guided by literature review, expert panel, patient focus group & data analysis
- Largely developed among 110 individuals representative of the target population
- Face validity assessed via a patient focus group not representative of the target population

For peer review only - http://bmjopen.bmj.com/site/about/guidelines.xhtml

INTRODUCTION

Cardiovascular disease (CVD) is a major cause of disability and premature mortality worldwide. In England it accounts for a third of deaths and costs the NHS and UK economy £30 billion annually.[1, 2] Modifiable lifestyle risk factors, associated with 90% of CVD,[3, 4] contributed to only 34% of the overall decline in CVD mortality in England between 2000-2007.[5] In 2010 / 2011 there were 1.4 million CVD related hospital admissions, of which 60% were for people younger than 75 and more than half as an emergency. Further gains could be made in preventing long term illness and disability associated with CVD while reducing healthcare costs by promoting healthier lifestyle changes.[6]

The National Health Service (NHS) Health Check programme may be important for preventing premature CVD while reducing healthcare costs therein by identifying individuals at increased risk of CVD, raising their awareness of CVD risk and helping them manage their risk. [7-10] This CVD risk assessment and management programme was launched by the Department of Health in April 2009 in England among 40-74 year olds free of vascular disease diagnosis.[7] It aims to prevent heart disease, stroke, diabetes and kidney disease whilst reducing health inequalities. Individuals' socio-demographics, cholesterol, blood pressure, smoking, and family history of CVD are used to predict CVD risk.[11] In addition to lifestyle advice given to all participants, people at high risk of CVD are invited for further consultations and offered statins and behaviour change support in relation to physical activity, smoking cessation, safe alcohol consumption and healthy diet. Projected programme cost is £180-£243 million/year with estimated cost per quality adjusted life year (QALY) at £3,000.[10]

To adopt healthy lifestyle behaviours related to diet, exercise, smoking and alcohol consumption, the general population must be aware of CVD risk.[12] In the context of the NHS Health Check Programme, CVD risk awareness refers to the accuracy of perceived risk of CVD against predicted CVD risk, general knowledge of CVD and what one can do to lower predicted CVD risk. Whereas predicted CVD risk refers to one's chance of experiencing a heart attack or stroke,[11] perceived risk of CVD refers to a person's perception of their CVD risk. While as many as 40% of the general population underestimate their CVD risk, 20% overestimate their risk.[13] False reassurance may lead to adoption and or maintenance of unhealthy behaviours contributing to the premature onset of CVD. Low CVD risk awareness is reported among men, inner city residents, and people of lower socioeconomic status. [12] [14] [15] It is not known if the Health Check results in improved CVD risk awareness.

Although several validated questionnaires measure knowledge, perceptions of CVD or intention to change behaviour, [15-17] no short, validated questionnaire assesses CVD risk awareness using all of these scales. Until now studies examining the accuracy of perceived risk and knowledge of CVD relied on non-validated tools.[16] The problem with using non-validated tools is that the questions may not accurately and reliably capture individuals' views or measure what they intend to measure. The aim of this work was to develop a questionnaire with satisfactory face, content validity and reliability to assess patients' awareness of CVD risk among NHS Health Check attendees.

METHODS

For peer review only - http://bmjopen.bmj.com/site/about/guidelines.xhtml

The first phase of development of the questionnaire was guided by a literature review, an expert panel and a patient focus group. At each stage of questionnaire development, the number of items was reduced (see Figure 1).

Figure 1 Flowchart of Phase I of Questionnaire Development

The second phase of questionnaire development was guided by an analysis of data from 110 NHS Health Check attendees who completed the 65 item questionnaire. The number of questionnaire items was further reduced (see Figure 2).

Figure 2 Flowchart of Phase II of Questionnaire Development

Phase I of Questionnaire Development

Construction of draft questionnaire by review of relevant literature

We performed an extensive literature review pertaining to CVD risk awareness between December 2013 and January 2014 in the areas of disease knowledge, risk perception, intention to change and self-efficacy related to CVD and the Health Belief Model (HBM) to guide initial item development. PubMed and PsycINFO databases and Google Scholar Articles were utilised to search for existing instruments that measure perception of CVD risk, CVD knowledge and self-efficacy with no limits on the year of publication. The following key words were used to identify the relevant literature: “cardiovascular disease” “heart disease” “knowledge” “risk” “test” “questionnaire” “scale” “assessment” “self-efficacy” “perception” “health belief model”. Questionnaires were considered if they addressed CVD risk awareness, reported moderate to high scores of reliability and validity in population studies and had suitable wording and level of understanding. Questionnaires were excluded if they pertained to individuals under the age of 15 as these people would not be eligible to receive an NHS Health Check, focused on risk unrelated to heart attack or stroke, and were not written in English.

Although a number of questionnaires were found measuring different aspects of CVD risk awareness such as heart disease knowledge, perception of CVD risk, perceived susceptibility and severity of CVD and benefits and barriers to adopting healthy behaviours,[17-19] no single questionnaire encompassed them all. Initial item development was guided by HBM[20] and the Transtheoretical Model (TTM).[21] According to HBM, individuals who have accurate knowledge of CVD and perceived susceptibility to and consequences of the disease, and are aware of the benefits of taking preventive measures are more likely to make important lifestyle choices to prevent the onset of disease.[22] The TTM describes behavioural change as a staged process over time including pre-contemplation, contemplation, preparation, action and maintenance.[21] Sixty five items were selected using validated questionnaires addressing CVD knowledge, and the main constructs of HBM such as perceived susceptibility, perceived severity, perceived benefits of changing behaviours, and perceived barriers to making changes.[17-19] In addition 23 new items were generated to identify perceived levels of readiness to engage in CVD risk reduction behaviours (using TTM) and self-efficacy (confidence in ability to change health behaviour) in relation to exercise, diet, smoking cessation and decreasing alcohol consumption.[23, 24] These items were based on data collected during an NHS Health Check and behaviour specific recommendations such as stopping smoking, consuming no more than 14 units of

For peer review only - http://bmjopen.bmj.com/site/about/guidelines.xhtml

alcohol a week, eating at least five portions of fruit and vegetables a day and exercising at least 150 minutes per week.[25-28] The resulting 85 item questionnaire is in Appendix A.

Modification of questionnaire by expert panel to obtain satisfactory content validity

A panel of experts in the areas of CVD, health psychology, public health, psychometrics and questionnaire development and medicine were asked to evaluate each item and the total 85 item questionnaire for content validity in February 2014. Experts assessed content validity of the questionnaire by examining whether the items were representative of the content they were intended to measure.[29] Items were examined for representatives of the scale domain, appropriateness and relevance. The content validity index (CVI), a widely used technique in scale development determined item and questionnaire clarity, homogeneity, and relevance on a 4-point Likert scale (ranging from 1 = *an irrelevant item* to 4 = *an extremely relevant item*).[30, 31] A CVI of ≥ 0.80 is recommended.[32, 33] Experts were asked the following questions: "*Do these items belong together in the subscale?*" and "*Does each item belong in the set?*" For ratings of content validity, experts were asked whether the subscale definition and label fitted the set of items presented; whether each item belonged with the label and definition; and whether each item was unique in its contribution to the subscale.

Modification of questionnaire by patient focus group to obtain satisfactory face validity

Researchers facilitated a patient focus group to assess the face validity of the 69 item questionnaire resulting from the expert review. Face validity is assessed by end users deciding whether the questionnaire appears to measure what the researchers who developed it claim.[33] A convenience sample of six individuals was recruited on March 4, 2014 from the County Durham and Darlington National Health Service Foundation Trust. Eligibility criteria were being aged 40-74 years and being free of known vascular disease. The focus group consisted of six white females between 50-64 years of age. Most participants had postgraduate education. These individuals worked as clerical workers, nurses and health improvement staff. They were not involved in the delivery of the NHS Health Check programme. Participants were asked to complete the 69 item questionnaire as well as to provide feedback on whether the items correctly measured the intended scales, appropriately stated the intent of the questionnaire, and matched the individual's situations.[32, 33] In addition, participants were asked to respond to questions about clarity, content, appropriateness, format, biases of questions and presentation of information. The resulting 65 item questionnaire is in Appendix B.

Phase II of Questionnaire Development

Modification of questionnaire to have satisfactory reliability

A 65 item questionnaire was administered to 110 NHS Health Check attendees immediately after their consultation between May 21 and July 28, 2014 in a cross sectional study in England. The aim was to determine the content, the scale structure and the reliability of the resulting questionnaire.

Study Recruitment

Eligibility criteria were completion of an NHS Health Check, being aged 40-74 years and free of known vascular disease. Of 110 study participants, 15 individuals were recruited by 2 nurses from a London general practice and 95 individuals by 13 community outreach providers from local

For peer review only - http://bmjopen.bmj.com/site/about/guidelines.xhtml

community venues in Durham. These providers collected clinical risk factor data, informed study participants about their CVD risk, took informed study consent and distributed the 65 item questionnaire to be self-completed by NHS Health Check attendees following their consultation. Unlike general practice staff who operated only during business hours, community outreach providers worked on evenings and weekends as well as during regular business hours in community venues more accessible to the general public.

Data Analysis

To select appropriate items to constitute a scale, individual items were assessed during item analysis, item facility and item discrimination.[34] To determine the factorial structure of the questionnaire and which items together constituted particular scales, an Exploratory Factor Analysis (EFA) - a widely used technique in scale development was performed.[30, 35] The reliability of factors constituting particular scales was assessed using Cronbach's alpha coefficient. [36, 37] Reliability refers to consistency, reproducibility and agreement of a scale.[38]

To improve the quality of a scale and increase its reliability, individual items were assessed. Items with reverse scoring were re-coded to conform to the conceptual direction of the scales.[37] Each individual item was then examined for distortions in the pattern of responding known as skew and kurtosis.[33] Item facility examined whether items were answered in the same way by everyone by checking whether the facility index approached extreme scores or had a low standard deviation.[34] Items were assessed in discriminating between participants' responses to the questionnaire's scales (Knowledge, Perceived CVD Risk, CVD Health Beliefs, Intentions / Readiness to Change and Self Efficacy). Discrimination was measured by item-total correlation with item correlating below 0.2 or any negative correlations resulting in deletion of items. In addition, discrimination was measured by the inter-item correlation within each scale resulting in deletion of items correlating with other items ≥0.60.[17, 34]

A Kaiser-Meyer-Olkin (KMO) measure of sampling adequacy and a Bartlett's test of sphericity were assessed to ensure that items were appropriate for EFA.[39] Next EFA was performed to define the scales of the questionnaire which share a similar underlying construct. Parallel Analysis was used to determine the optimum number of factors to be extracted using Principal Components Analysis (PCA) with a Varimax rotation. [34, 39, 40] PCA is a data reduction technique used to explain correlations among sets of items or variables as a few conceptually meaningful factors.[30] Compared to other available methods, Parallel Analysis using PCA was shown to be the best method of extracting factors and is appropriate when applied to data conforming to the formal factor analytic model.[39, 40] Iterations of EFA were carried out to identify core constituent items in each factor. Cross-loading items or items with loading <=0.50 were removed at each iteration.[39] Internal consistency reliability of resulting factors was assessed using Cronbach's α coefficients with α>=0.70 indicating good reliability.[32,36, 37] Associations between resulting factors and predicted CVD risk were examined using Spearman's rank correlation coefficient.

RESULTS

Construction of a draft questionnaire by review of relevant literature

For peer review only - http://bmjopen.bmj.com/site/about/guidelines.xhtml

We developed an 85 item questionnaire based on the theoretical framework, NHS guidelines and other validated questionnaires relating to heart disease.[17-19] The 85 item questionnaire had 8 subscales measuring *Knowledge of CVD Risk and Prevention* (18 items), *Perceived Risk and Vulnerability of CVD* (20 items), *Perceived Susceptibility* (5 items), *Perceived Severity* (5 items), *Perceived Benefits* (6 items), *Perceived Barriers* (7 items), *Self-Efficacy* (6 items), and *Intention to Change Behaviour* (18 items). *Knowledge of CVD Risk and Prevention* subscale items were measured using the following categories: *True, False*, and *Don't Know*. *Self-Efficacy* subscale items were measured using 5 point Likert scale ranging from *1=not at all confident* to *5=completely confident*. *Perceived Severity, Perceived Benefits, Perceived Barriers and Intention to Change Behaviour* subscale items were measured using a 4 point Likert scale ranging from *1=strongly disagree* to *4=strongly agree*. The reading level of the questionnaire was at Year 7.

Modification of questionnaire by expert panel to obtain satisfactory content validity

The expert panel concluded that out of the 85 items, 69 met the CVI>=0.80 criterion and were retained. In addition, the wording of a number of questions was revised to improve clarity. Diet and exercise were defined more precisely using frequency and duration. Response options of Self-Efficacy items were changed from a five point Likert scale to a four point Likert scale for consistency with the rest of the questionnaire. Questions pertaining to smoking and drinking were rephrased to apply to smokers and drinkers (see Table 1).

Table 1 Sample item wording modifications obtained through an expert panel

**Original item(s)**	**Expert comments**	**Final item**
The most important cause of heart attack and stroke is stress.	Revise to "one of the most important…" Substitute the word "important" with "main."	One of the main causes of heart attack and stroke is stress.
I have a high chance of getting a heart attack or stroke because of my past behaviours.	Add "and/or present behaviours."	I have a high chance of getting a heart attack or stroke because of my past and/or present behaviours.
Increasing my exercise will decrease my chances of having a heart attack or stroke.	Define amount of exercise.	Increasing my exercise to at least 30 minutes a day will decrease my chances of having a heart attack or stroke.
Eating a healthy diet will decrease my chance of having a heart attack or stroke.	Define a healthy diet.	Eating at least five portions of fruit and vegetables a day will decrease my chances of having

For peer review only - http://bmjopen.bmj.com/site/about/guidelines.xhtml

For peer review only

Original item(s)	Expert comments	Final item
		a heart attack or stroke.
When I exercise I am doing something good for myself.	Define exercise consistently. Make the statement more specific about the heart.	When I exercise for 30 minutes a day I am doing something good for the health of my heart.
How confident are you that you know or can…? questions answered using a 5-point Likert scale: “not at all confident, somewhat confident, moderately confident, very confident, completely confident.”	Use a 4-point Likert to maintain consistency.	Five point Likert scale changed to a 4 point Likert scale: “not at all confident, somewhat confident, very confident, completely confident.”
How confident are you that you know how or can stop smoking if you want to?	Instead of saying “…that you know or can” say “that you know how to or can…” Add in parentheses “if you smoke.”	How confident are you that you know how to or can stop smoking if you want to (if you smoke)?
I want to cut down on alcohol. I intend to cut down on alcohol in the next two months.	Conceptual overlap between want to and intend to. Add in parentheses “if you drink alcohol.”	I intend or want to cut down on alcohol (if you drink alcohol).

Modification of questionnaire by patient focus group to obtain satisfactory face validity

As a result of the focus group review of the 69 item questionnaire, six items were removed, two items were added and a number of items were modified leaving a final total of 65 items with satisfactory face validity. A not applicable category was added to 50 items while the response categories to Knowledge subscale items remained unchanged. Exercise was redefined in 8 items from 150 minutes a week and 30 minutes a day to 2.5 hours a week. A negatively framed question was reframed positively (see Table 2).

Table 2 Sample item wording modifications and additions through the patient focus group

Original item	Participant comments	Final item
Moderate physical activity of 150 minutes a week will	2.5 hours a week is better than 150 minutes.	Moderate physical activity of 2.5 hours a week will reduce

For peer review only - http://bmjopen.bmj.com/site/about/guidelines.xhtml

Original item	Participant comments	Final item
reduce your chances of developing a heart or stroke.		your chances of developing a heart or stroke.
Drinking alcohol has nothing to do with reducing the risk of heart attack or stroke.	Question is negatively stated.	Drinking high levels of alcohol can increase your cholesterol and triglyceride levels.
Missing question	Need to include family history of disease to account for genetic predisposition.	A family history of hypertension is not a risk factor for high blood pressure.
Missing question	Benefits of not smoking?	If I stopped smoking it will reduce my chances of having a heart attack or stroke.
Increasing my exercise for 30 minutes a day will decrease my chances of having a heart attack or stroke.	Two and a half hours a week is better than 30 minutes a day.	Increasing my exercise to at least 2 ½ hours a week will decrease my chances of having a heart attack or stroke.
I have reduced or stopped smoking (if you smoke).<br>“strongly disagree, disagree, agree, and strongly agree.”	Remove (if you smoke).<br>Add a “not applicable” box.	I have reduced or stopped smoking.<br>“strongly disagree, disagree, agree, and strongly agree, not applicable.”
How confident are you that you know how to or can consume recommended levels of alcohol (if you drink alcohol)?<br>“not at all confident, somewhat confident, very confident and completely confident.”	Remove (if you drink alcohol).<br>Add a “not applicable” box.	How confident are you that you know how to or can drink within the recommended levels of alcohol?<br>“not at all confident, somewhat confident, very confident and completely confident, not applicable.”

Modification of questionnaire to have satisfactory reliability

The 65 item questionnaire that resulted from content and face validity assessments, was administered to 110 NHS Health Check attendees immediately after their NHS Health Check consultation. Most study participants were White (84.5%), younger than 60 (77.3%) and had at least

For peer review only - http://bmjopen.bmj.com/site/about/guidelines.xhtml

one or more CVD risk factors. Using the Index of Multiple Deprivation, a relative measure of deprivation across seven distinct domains including income, employment, health and disability, education skills and training, barriers to housing and services, living environment and crime, [41] people in the two most deprived fifths were 40.0% of the study population. See Appendix C for study population characteristics. The responses to the questionnaire were analysed as individual items during item analysis, item facility and item discrimination. In addition, the scale structure and reliability of resulting scales were assessed.

No items were removed during item analysis and item facility. During item discrimination assessment using item-total correlation, seven items in the Knowledge scale, four items in Perceived CVD Risk, three items in CVD Health Benefits, three items in Intention and or Readiness to Change were deleted due to item-total correlations falling below 0.2. [33] During item discrimination assessment using inter-item correlation, two items in Perceived CVD Risk and three items in Intentions / Readiness to Change were removed as these items correlated greater than 0.6 with other items.[33] Although there were two items that correlated above 0.6 in CVD Risk Reduction Self Efficacy, these remained in the questionnaire as the items were qualitatively different: *Stop smoking if you want to* and *Control the risks of having a heart attack or stroke.* In total, 22 items were removed during item discrimination analysis, leaving 43 items which had good item facility and discrimination.

Of the 43 remaining items, 8 items of the "Knowledge" scale with *"true"* or *"false"* scoring could not be entered into EFA. Of the 35 items scored on a four point Likert scale, four items pertaining to smoking were deleted as they had a high proportion of missing responses (69-80%). The resulting 31 items had a Kaiser-Meyer-Olkin (KMO) measure of sampling adequacy of 0.32 and a significant Bartlett's test of sphericity (1020.50, p <.001), indicating that these data were appropriate for EFA.[39] After 12 iterations of EFA, 20 items loaded above 0.50 on the factors and there were no cross-loadings indicating good factor structure (see Table 3). Internal consistency reliability of factor structure was measured using Cronbach's α. Factor 1 (8 items): (Perceived Risk of Heart Attack/Stroke) had α =.85. Factor 2 (7 items): (Perceived Benefits & Intentions to Change) had α =.82. Factor 3 (3 items): (Healthy Eating Intentions) had α =.56. Factor 4 (2 items): (Intentions towards Alcohol) had α =-0.16. Although Healthy Eating Intentions α = 0.56 is below the minimum threshold (0.70) for reliability, this is acceptable for a three item scale.[34] The intention toward alcohol factor had two items with such low reliability (α = -0.16) that they could not be considered a separate factor and were removed. A thirteenth EFA iteration confirmed the factor loadings and reliabilities reported above. Hence the parallel analysis indicated that three factors should be retained. [39] The three factor model accounted for 57.61% of the total explained variance.

Table 3 Factor structure of the ABCD Risk Questionnaire

	Components

For peer review only - http://bmjopen.bmj.com/site/about/guidelines.xhtml

	Factor 1 Perceived Risk of Heart Attack / Stroke	Factor 2 Perceived Benefits & Intentions to Change	Factor 3 Healthy Eating Intentions
It is likely that I will suffer from a heart attack or stroke in the future.	.844		
It is likely that I will have a heart attack or stroke some time during my life.	.816		
I feel I will suffer from a heart attack or stroke sometime during my life.	.809		
There is a good chance I will experience a heart attack or stroke in the next 10 years.	.752		
I am not worried that I might have a heart attack or stroke.	.705		
My chances of suffering from a heart attack or stroke in the next 10 years are great.	.687		
It is likely I will have a heart attack or stroke because of my past and/or present behaviours.	.639		
I am concerned about the likelihood of having a heart attack or stroke in the near future.	.575		
I am thinking about exercising at least 2 ½ hours a week.		.826	
I intend or want to exercise at least 2 ½ hours a week.		.792	
When I exercise for at least 2½ hours a week I am doing something good for the health of my heart.		.735	
I am confident that I can maintain a healthy weight by exercising at least 2½ hours a week within the next two months.		.658	

	Components		
	Factor 1 Perceived Risk of Heart Attack / Stroke	Factor 2 Perceived Benefits & Intentions to Change	Factor 3 Healthy Eating Intentions
I am not thinking about exercising for 2 ½ hours a week.		.656	
When I eat at least five portions of fruit and vegetables a day I am doing something good for the health of my heart.		.642	
Increasing my exercise to at least 2½ hours a week will decrease my chances of having a heart attack or stroke.		.557	
I am confident that I can eat at least five portions of fruit and vegetables per day within the next two months.			.830
I am thinking about eating at least five portions of fruit and vegetables a day.			.772
I am not thinking about eating at least five portions of fruit and vegetables a day.			.731
Note: Factor loadings and commonalities are reported following an EFA using Principal Component Analysis with Varimax rotation.			

The EFA revealed three scales: Perceived Risk of Heart Attack / Stroke, Perceived Benefits and Intentions to Change and Healthy Eating Intentions. A fourth scale assessing Knowledge of CVD Risk and Prevention (not entered into EFA) was added back to the questionnaire following EFA (see Figure 2). Hence the resulting questionnaire included 26 items grouped into four scales: Knowledge of CVD Risk and Prevention (8 items), Perceived Risk of Heart Attack/Stroke (7 items), Perceived Benefits and Intention to Change Behaviour (7 items) and Healthy Eating Intentions (3 items). In the resulting 26 item questionnaire, two items were changed from questions "How confident are you that you know how to or can…" to statements of agreement "I am confident that I can" so as to be answered using the same Likert scale. The time to complete this questionnaire is between 10-15 minutes. The ABCD Risk Questionnaire with a scoring guide for each scale is reported in Appendix D. Using Spearman's rho, there was a positive and significant relationship between perceived and predicted CVD risk (Appendix E).

For peer review only - http://bmjopen.bmj.com/site/about/guidelines.xhtml

DISCUSSION

To the best of our knowledge this is the first study that describes the development of a short, validated questionnaire with satisfactory content and face validity and reliability examining CVD risk awareness among the NHS Health Check attendees. The ABCD Risk Questionnaire may be used for evaluating the accuracy of perceived CVD risk, general knowledge of CVD and intention to change behaviour in regards to diet and exercise among NHS Health Check attendees. Agreement between perceived and predicted CVD risk suggests that the tool performs well in assessing perceived CVD risk. As the questionnaire was developed using both an expert panel and a patient focus group, it ought to be relatively easy to understand for both patients and clinicians. If NHS Health Check recommendations change over time, it may need to be updated.

Critics of the NHS Health Check programme point to the lack of its evidence base. [42, 43] The majority of evaluations focused on coverage and uptake, statin prescribing, new diagnoses and CVD risk factor reduction. [44-49] As there was no instrument measuring CVD risk awareness, no studies examined the patients' understanding of CVD risk among NHS Health Check attendees. CVD risk presentation was shown to increase the accuracy of perceived risk by 10%. When risk information is repeated this leads to small but significant reductions in predicted CVD risk.[16] A national study showed modest reductions in 10 year predicted CVD risk among NHS Health Check attendees in the first four years.[48] A limitation of using predicted ten year risk of CVD is the under-estimation of CVD risk among women and younger people. [35] More research is needed to establish whether the programme improves NHS Health Check attendees' awareness of CVD risk and whether the programme has an impact on predicted lifetime CVD risk.

The ABCD Risk Questionnaire was developed on a non-risk stratified population after their initial NHS Health Check consultation as the NHS Health Check programme is administered to all eligible people free of vascular disease diagnosis irrespective of their level of CVD risk. The questionnaire does not encompass all aspects of CVD risk observed in the general population. Questions on smoking and drinking were progressively eliminated as they did not apply to most study participants. As questions on diet and exercise pertained to all people regardless of their level of CVD risk, such questions that reliably distinguished between study participants were selected for inclusion. Although fruit and vegetable intake is only one aspect of diet in the EatWell Guide recommended for use in NHS Health Check,[50] it is the only assessment of diet recorded during the NHS Health Check. The resulting questionnaire contains questions based on data collected during NHS Health Check to enable subsequent programme evaluation.[51] Future studies examining populations at increased CVD risk can look into incorporating smoking and alcohol into the ABCD Risk Questionnaire to learn about these individuals' preconceptions and attendance of follow up care.

Judging by the number of items reduced in various stages of development, the ABCD Risk Questionnaire was largely shaped by analysis of data from 110 NHS Health Check attendees completing the 65 item questionnaire. This study population was representative of the population that took up the NHS Health Check programme between 2009-2014 in terms of socio-demographics including the proportion of men (46.4%), ethnic minorities (5.4%), individuals from the most deprived two fifths (40.9%), and clinical risk factors including mean total cholesterol (5.42 (95% CI 5.19, 5.64)), BMI (27.24 95% CI 26.17, 28.31), smokers (18.2%) and those at high CVD risk (4.5%).[44] As higher levels of deprivation are partly due to having less education,[41] questionnaire development

For peer review only - http://bmjopen.bmj.com/site/about/guidelines.xhtml

was not limited to people with higher education. Compared to the national evaluation, similar levels of high CVD risk were observed despite the fact that the study population contained more younger people aged 40-59 (77.3%).[44] The recruitment of hard to reach groups including younger people, socio-economically deprived individuals and ethnic minorities by community outreach providers in community venues outside of conventional working hours is consistent with prior literature. [22, 52, 53, 54]

A possible limitation to face validity is that the patient focus group evaluating the 69 item questionnaire was not representative of the target population. Whereas the NHS Health Check programme is administered to both men and women and members of ethnic minorities, the focus group consisted only of white women. Furthermore, as these women had postgraduate education and worked in a health-related field, they may have had higher health literacy than the general population eligible for the NHS Health Check programme. Clarity, appropriateness, biases and presentation of information may have been differentially assessed by people with different levels of health literacy. A community based recruitment method aiming to recruit some of the hard to reach groups may have been more effective in getting a more representative patient focus group.

Additional studies should be conducted with larger samples to confirm the reliability and validity of the questionnaire. It would be useful to replicate the factor analytic process on an independent, larger sample to confirm the generalizability of these findings.[37]

CONCLUSIONS

The ABCD Risk Questionnaire showed evidence of satisfactory reliability and validity, is brief and easy to use. By capturing patients' views on CVD risk awareness during an NHS Health Check consultation, the questionnaire can be used to assess patients' understanding of CVD risk. Clinicians administering the questionnaire to patients may help to establish whether the programme is effective in empowering patients to make informed lifestyle choices about their health.

References

1. World Health Organisation. Prevention of Cardiovascular Disease - Guidelines for assessment and management of cardiovascular risk, 2007. http://www.who.int/cardiovascular_diseases/guidelines/Full%20text.pdf (accessed 08 March 2015)
2. Murray CJL, Richards MA, Newton JN, et al. UK health performance: findings of the Global Burden of Disease Study 2010. *The Lancet*. 2013; 381: 997-1020.
3. Barton GR, Goodall M, Bower P, Woolf S, Capewell S and Gabbay MB. Increasing heart-health lifestyles in deprived communities: economic evaluation of lay health trainers. *Journal of Evaluation in Clinical Practice*. 2012; 18: 835-40.
4. Yusuf S, Hawken S, Ôunpuu S, et al. Effect of potentially modifiable risk factors associated with myocardial infarction in 52 countries (the INTERHEART study): case-control study. *The Lancet*. 2004; 364: 937-52.
5. Bajekal M, Scholes S, Love H, et al. Analysing Recent Socioeconomic Trends in Coronary Heart Disease Mortality in England, 2000–2007: A Population Modelling Study. *PLoS Med*. 2012; 9: e1001237.
6. The National Institute for Health and Care Excellence (NICE). Services for the prevention of cardiovascular disease - NICE commissioning guides [CMG45], 2012.

For peer review only - http://bmjopen.bmj.com/site/about/guidelines.xhtml

ttps://www.nice.org.uk/guidance/cmg45/chapter/commissioning-services-for-the-prevention-of-cardiovascular-disease (accessed 12 June 2015)
7. Department of Health. Putting prevention first- vascular checks: risk assessment and management - next steps guidance for primary care trusts, 2009. http://www.dh.gov.uk/en/Publicationsandstatistics/Publications/PublicationsPolicyAndGuidance/DH_090277 (accessed 7 July 2016).
8. Public Health England. NHS Health Check Best Practice Guidance 2015, 2015. http://www.healthcheck.nhs.uk/commissioners_and_healthcare_professionals/national_guidance/ (accessed 6 March 2015)
9. Public Health England. NHS Health Check: Our Approach to the Evidence, 2013. https://www.gov.uk/government/uploads/system/uploads/attachment_data/file/224537/NHS_Health_Check_our_approach_to_the_evidence_v2.pdf;(accessed 12 June 2015).
10. Department of Health. Vascular Checks: Economic Modelling for Vascular Checks, 2008. http://www.dh.gov.uk/en/Publicationsandstatistics/Publications/PublicationsPolicyAndGuidance/DH_085869 (accessed 12 June 2015).
11. Collins GS and Altman DG. Predicting the 10 year risk of cardiovascular disease in the United Kingdom: independent and external validation of an updated version of QRISK2. *BMJ*. 2012; 344.
12. Homko CJ, Santamore WP, Zamora L, et al. Cardiovascular Disease Knowledge and Risk Perception Among Underserved Individuals at Increased Risk of Cardiovascular Disease. *Journal of Cardiovascular Nursing*. 2008; 23: 332-7
13. Webster R and Heeley E. Perceptions of risk: understanding cardiovascular disease. *Risk management and healthcare policy*. 2010; 3: 49-60.
14. Kirkland SA, MacLean DR, Langille DB, Joffres MR, MacPherson KM and Andreou P. Knowledge and awareness of risk factors for cardiovascular disease among Canadians 55 to 74 years of age: results from the Canadian Heart Health Surveys, 1986-1992. *CMAJ : Canadian Medical Association journal = journal de l'Association medicale canadienne*. 1999; 161: S10-6.
15. Potvin L, Richard L and Edwards AC. Knowledge of cardiovascular disease risk factors among the Canadian population: relationships with indicators of socioeconomic status. *CMAJ : Canadian Medical Association journal = journal de l'Association medicale canadienne*. 2000; 162: S5-11.
16. Sheridan SL, Viera AJ, Krantz MJ, et al. The Effect of Giving Global Coronary Risk Information to Adults: A Systematic Review. *Archives of Internal Medicine*. 2010; 170: 230-9.
17. Bergman HE, Reeve BB, Moser RP, Scholl S and Klein WMP. Development of a Comprehensive Heart Disease Knowledge Questionnaire. *American Journal of Health Education*. 2011; 42: 74-87.
18. Ammouri AA and Neuberger G. The Perception of Risk of Heart Disease Scale: development and psychometric analysis. *J Nurs Meas*. 2008; 16: 83-97.
19. Tovar EG, Rayens MK, Clark M and Nguyen H. Development and psychometric testing of the Health Beliefs Related to Cardiovascular Disease Scale: preliminary findings. *Journal of advanced nursing*. 2010; 66: 2772-84.
20. Strecher V.J. R, I.M. *The Health Belief Model. In Health Behavior and Health Education, Theory, Research and Practice (Glanz, K., Lewis, F.M., Rimer, B.K. eds)*. San Francisco: Jossey-Bass, 1997.
21. Prochaska JO, Redding, C.A., Evers, K.E. . *The Transtheoretical Model and Stages of Change. In Health Behavior and Health Education, Theory, Research and Practice (Glanz, K., Lewis, F.M., Rimer, B.K. eds).* . San Francisco: Jossey-Bass, 1997.
22. Hsu HY and Gallinagh R. The relationships between health beliefs and utilization of free health examinations in older people living in a community setting in Taiwan. *Journal of advanced nursing*. 2001; 35: 864-73.
23. Prochaska JO, DiClemente CC and Norcross JC. In search of how people change. Applications to addictive behaviors. *Am Psychol*. 1992; 47: 1102-14.

For peer review only - http://bmjopen.bmj.com/site/about/guidelines.xhtml

24. Prochaska JO, Norcross JC and DiClemente CC. *Changing for Good: A Revolutionary Six-Stage Program for Overcoming Bad Habits and Moving Your Life Positively Forward.* New York, NY Avon Books, 1994.
25. Department of Health. Physical Activity Guidelines for Adults (19-64 Years), 2011. http://www.nhs.uk/Livewell/fitness/Documents/adults-19-64-years.pdf (accessed 23 April 2015).
26. Department of Health. Improve Your NHS Health Check Numbers, 2014. http://www.nhs.uk/Conditions/nhs-health-check/Pages/Take-action-to-improve-your-health.aspx (accessed 23 April 2015).
27. Department of Health. Five a Day, 2013. http://www.nhs.uk/livewell/5aday/Pages/ADAYhome.aspx (accessed 23 April, 2015).
28. Health Do. Putting prevention first. NHS health check: Vascular risk assessment and management - best practice guidance. 2009.
29. Andrew S and Halcomb EJ. *Mixed Methods Research for Nursing and the Health Sciences*. Blackwell Publishing Ltd, 2009.
30. Chan CW. Perceptions of coronary heart disease: the development and psychometric testing of a measurement scale. *Psychol Health Med*. 2014; 19: 159-68.
31. Wells KJ, Arevalo M, Meade CD, et al. Development and validation of the biobanking attitudes and knowledge survey (BANKS). *Cancer Epidemiol Biomarkers Prev*. 2014; 23: 374-82.
32. Waltz CF, Strickland OL and Lenz ER. *Measurement in Nursing Research*. Second Edition ed. Philadelphia: F.A. Davis Company, 1991.
33. Waltz CF, Strickland OL and Lenz ER. *Measurement in Nursing and Health Research* Fourth Edition ed. New York: Springer Publishing Company, 2010.
34. Rust J and Golombok S. *Modern psychometrics: The science of psychological assessment.* Second Edition ed. London: Routledge, 1999.
35. JBS 3 Joint British Societies for the Prevention of Cardiovascular Disease. Lifetime Risk, 2014. http://www.jbs3risk.com/pages/lifetime_risk.htm (accessed 9 August 2016).
36. Nunnally JC and Bernstein IH. *Psychometric theory*. Third Edition ed. New York: McGraw-Hill Higher, INC, 1994.
37. DeVellis RF. *Scale Development Theory and Applications*. Third Edition ed. Los Angelos / London / New Delhi / Singapore / Washington DC: SAGE, 2012.
38. Aldridge V, Wade A and Koutoumanou E. Validity and Reliability University College London, Institute of Child Health2015.
39. Ferguson E and Cox T. Exploratory Factor Analysis: A Users'Guide. *International Journal of Selection and Assessment*. 1993; 1: 84-94.
40. Zwick WR and Velicer WF. Comparison of five rules for determining the number of components to retain. *Psychological Bulletin*. 1986; 99: 432-42.
41. Department for Communities and Local Government. The English Indices of Deprivation 2010 - Neighborhoods Statistical Release, 2011. https://www.gov.uk/government/uploads/system/uploads/attachment_data/file/6871/1871208.pdf (accessed 16 December 2014).
42. Krogsbøll Lasse T, Jørgensen Karsten J, Grønhøj Larsen C and Gøtzsche Peter C. General health checks in adults for reducing morbidity and mortality from disease. *Cochrane Database of Systematic Reviews*: (2012).
43. Capewell S and Graham H. Will cardiovascular disease prevention widen health inequalities? *PLoS Med*. 2010; 7: e1000320.
44. Chang KC-M, Soljak M, Lee JT, et al. Coverage of a national cardiovascular risk assessment and management programme (NHS Health Check): Retrospective database study. *Preventive Medicine*.
45. Artac M, Dalton ARH, Majeed A, Car J and Millett C. Effectiveness of a national cardiovascular disease risk assessment program (NHS Health Check): Results after one year. *Preventive Medicine*. 2013.

For peer review only - http://bmjopen.bmj.com/site/about/guidelines.xhtml

46. Artac M, Dalton ARH, Majeed A, Car J, Huckvale K and Millett C. Uptake of the NHS Health Check programme in an urban setting. *Family Practice*. 2013; 30: 426-35.
47. Dalton ARH, Bottle A, Okoro C, Majeed A and Millett C. Uptake of the NHS Health Checks programme in a deprived, culturally diverse setting: cross-sectional study. *J Public Health (Oxf)*. 2011; 33: 422-9.
48. Chang KC, Lee JT, Vamos EP, et al. Impact of the National Health Service Health Check on cardiovascular disease risk: a difference-in-differences matching analysis. *CMAJ : Canadian Medical Association journal = journal de l'Association medicale canadienne*. 2016.
49. Robson J, Dostal I, Sheikh A, et al. The NHS Health Check in England: an evaluation of the first 4 years. *BMJ Open*. 2016; 6: e008840.
50. NHS Choices. Eating a Balanced Diet. 2016. http://www.nhs.uk/Livewell/Goodfood/Pages/Healthyeating.aspx (accessed 27 April 2017).
51. Public Health England. NHS Health Check Best practice guidance. 2017. http://www.healthcheck.nhs.uk/commissioners_and_providers/guidance/national_guidance1/ (accessed 27 April 2017).
52. Grayland J and Wilson R. Improving male life expectancy in Birmingham. *Public Health*. 2009; 123: e50-e6.
53. Hunt BD, Hiles SL, Chauhan A, et al. Evaluation of the Healthy LifeCheck programme: a vascular risk assessment service for community pharmacies in Leicester city, UK. *J Public Health (Oxf)*. 2013; 35: 440-6.
54. Visram S, Carr SM and Geddes L. Can lay health trainers increase uptake of NHS health checks in hard-to-reach populations? A mixed-method pilot evaluation. *J Public Health (Oxf)*. 2014.

For peer review only

For peer review only - http://bmjopen.bmj.com/site/about/guidelines.xhtml

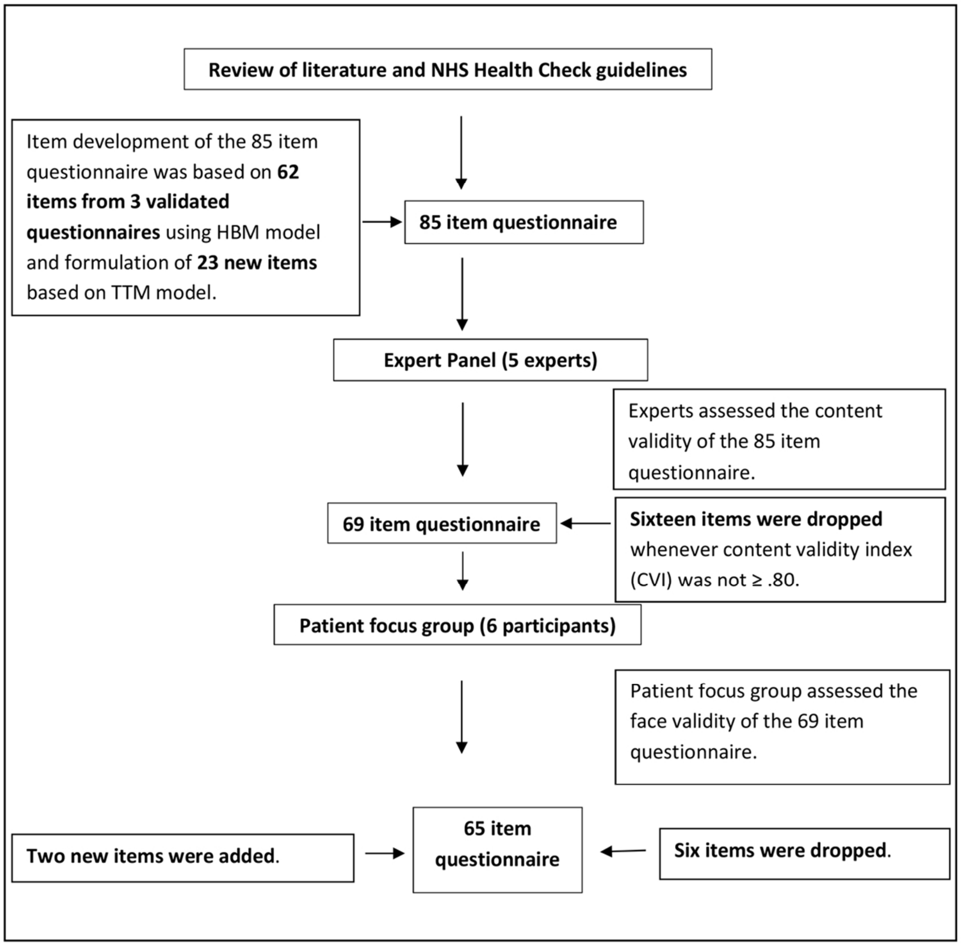

Figure 1

112x112mm (300 x 300 DPI)

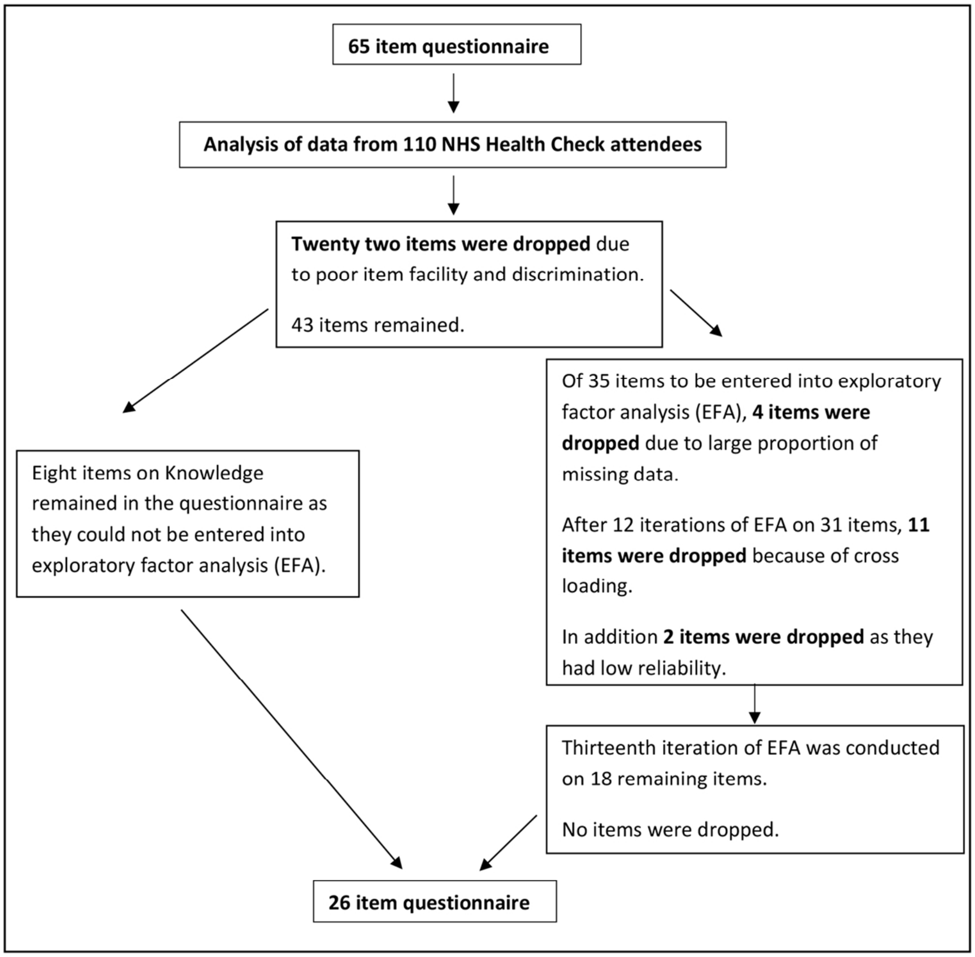

Figure 2

112x112mm (300 x 300 DPI)

Appendix A. 85 Item Questionnaire

Subscale	Items	Answers
Knowledge of CVD Risk and Prevention	1. Eating a lot of red meat increases heart attack and stroke risk.	True, False, Don't Know<br>T=True F=False<br>Correct Answers<br>Q1=T Q2=F Q3=T Q4=F Q5=T Q6=F Q7=F Q8=T Q9=T Q10=T Q11=F Q12=T Q13=T Q14=T Q15=T Q16=F Q17=T Q18=F
	2. Most people can tell whether or not they have high blood pressure.	
	3. You can reduce your risk of heart attack or stroke by being physically active.	
	4. 'High' blood pressure is defined as 110/80 (systolic/diastolic) or higher.	
	5. Dietary fibre lowers blood cholesterol.	
	6. The most important cause of heart attack and stroke is stress.	
	7. Trans-fats are healthier for the heart than most other kinds of fats.	
	8. Walking and gardening are considered types of exercise that can lower the risk of having a heart attack or stroke.	
	9. You can reduce your chance of developing a heart attack or stroke by eating five-a-day diet of fruits and vegetables.	
	10. Moderate physical activity of 150 minutes a week will reduce your chances of developing a heart attack or stroke.	
	11. People who quit smoking by 60 add five years to their life.	
	12. People who have diabetes are at higher risk having a heart attack or stroke.	
	13. Managing your stress levels will help you to manage your blood pressure.	
	14. HDL refers to 'good' cholesterol, and LDL refers to 'bad' cholesterol.	
	15. The healthiest exercise for the heart involves rapid breathing for a sustained period of time.	
	16. Many vegetables are high in cholesterol.	
	17. You are more likely to have a heart attack or stroke if you're overweight or obese.	
	18. Drinking alcohol has nothing to do with reducing the risk of heart attack or stroke.	
Perceived Risk and Vulnerability of CVD	19. There is a possibility that I will have a heart attack or stroke.	1 = Strongly disagree; 2 = disagree; 3 = agree; 4 = strongly agree
	20. There is a good chance I will experience a heart attack or stroke during the next 10 years.	
	21. A person who gets a heart attack or stroke has no chance of recovering.	
	22. I have a high chance of getting a heart attack or stroke because of my past behaviours.	
	23. I feel sure that I will have a heart attack or stroke.	
	24. Healthy lifestyle habits are unattainable.	
	25. It is likely that I will get a heart attack or stroke.	
	26. I am at risk for having a heart attack or stroke.	
	27. It is possible that I will have a heart attack or stroke.	
	28. I am not doing anything now that is unhealthy to my heart.	
	29. I am too young to have a heart attack or stroke.	
	30. People like me do not get a heart attack or stroke.	
	31. I am very healthy so I will not have a heart attack or stroke.	
	32. I am not worried that I might have a heart attack or stroke.	
	33. People my age are too young to have a heart attack or stroke.	

For peer review only

For peer review only - http://bmjopen.bmj.com/site/about/guidelines.xhtml

	34. People my age do not have a heart attack or stroke.	
	35. My lifestyle habits do not put me at risk for having a heart attack or stroke.	
	36. No matter what I do, if I am going to have a heart attack or stroke, I will have one.	
	37. People who do not have a heart attack or stroke are just plain lucky.	
	38. The causes of a heart attack or stroke are unknown.	
Perceived Susceptibility	39. It is likely that I will suffer from a heart attack or stroke in the future.	
	40. My chances of suffering from a heart attack or stroke in the next few years are great.	
	41. Having a heart attack or stroke is currently a possibility for me.	
	42. I feel I will suffer from a heart attack or stroke sometime during my life.	
	43. I am concerned about the likelihood of having a heart attack or stroke in the near future.	
Perceived Severity	44. Heart attacks and strokes are always fatal.	
	45. Having a heart attack or stroke will threaten my relationship with my significant other.	
	46. My whole life would change if I had a heart attack or stroke.	
	47. Having a heart attack or stroke would have a very bad effect on my sex life.	
	48. If I have a heart attack or stroke I will die within 10 years.	
Perceived Benefits	49. Increasing my exercise will decrease my chances of having a heart attack or stroke.	
	50. Eating a healthy diet will decrease my chance of having a heart attack or stroke.	
	51. Stopping smoking will reduce my chance of having a heart attack or stroke.	
	52. When I exercise I am doing something good for myself.	
	53. When I eat healthy I am doing something good for myself.	
	54. Cutting down on alcohol will decrease my chances of having a heart attack or stroke.	
Perceived Barriers	55. I do not know appropriate exercises to perform to reduce my risk of developing cardiovascular disease.	
	56. I do not know the recommended drinking limits for men or women.	
	57. I do not have time to exercise for 30 minutes a day on most days of the week.	
	58. I do not know what is considered a healthy diet that would prevent me from developing cardiovascular disease.	
	59. I will not have energy if I stop smoking.	
	60. I cannot afford to buy healthy foods.	
	61. I have other problems more important than worrying about diet and exercise.	
Self Efficacy	62. How confident are you that you know or can control the risks of having a heart attack or stroke?	1= not at all confident, 2=somewhat confident, 3= moderately confident, 4=very confident,
	63. How confident are you that you know or can maintain a healthy weight by exercising regularly?	
	64. How confident are you that you know or can stop smoking if you want to?	
	65. How confident are you that you know or can consume less alcohol?	

For peer review only

For peer review only - http://bmjopen.bmj.com/site/about/guidelines.xhtml

	66. How confident are you that you know or can control your blood pressure and/or cholesterol levels by taking your prescribed medications?	5=completely confident
	67. How confident are you that you know or can eat a healthy and balanced diet?	
Intention to Change Behaviour or Cues to Action	68. I want to stop smoking (if you do smoke).	1 = Strongly disagree; 2 = disagree; 3 = agree; 4 = strongly agree
	69. I intend to maintain a healthy weight.	
	70. I intend to be physically active within two months.	
	71. I expect to maintain a healthy weight.	
	72. I want to be physically active.	
	73. I intend to eat a healthy and balanced diet within two months.	
	74. I expect to stop smoking (if you do smoke).	
	75. I want to cut down on alcohol.	
	76. I want to maintain a healthy and balanced diet.	
	77. I intend to stop smoking within two months (if you do smoke).	
	78. I expect to eat a healthy and balanced diet.	
	79. I intend to cut down on alcohol in the next two months.	
	80. I expect to be physically active.	
	81. I expect to cut down on alcohol.	
	82. I want to eat a healthy and balanced diet.	
	83. I expect to take my medication to control my blood pressure and/or cholesterol	
	84. I want to take my medication to control my blood pressure and/or cholesterol	
	85. I intend to take my medication to control my blood pressure and/or cholesterol within two months	

For peer review only

Appendix B. 65 Item Questionnaire

Scale	Subscale	Items	Coding of Answers
**Knowledge**<br><br>(15 items)<br><br>Higher sum score = more knowledgeable / more correct	**CVD Risk Knowledge – Risk of having a heart attack / stroke**<br><br>(15 items)	1. Eating a lot of red meat increases heart attack and stroke risk.	Correct Answers<br><br>Q1-T Q6-T Q11-F<br>Q2-F Q7-T Q12-T<br>Q3-T Q8-T Q13-T<br>Q4-T Q9-T Q14-T<br>Q5-T Q10-T Q15-F<br><br>T= True<br>F= False<br><br>Correct: Score = 1, Incorrect or Don't Know Score = 0.
		2. Most people can tell whether or not they have high blood pressure.	
		3. You can reduce your risk of heart attack or stroke by being physically active.	
		4. One of the main causes of heart attack and stroke is stress.	
		5. Walking and gardening are considered types of exercise that can lower the risk of having a heart attack or stroke.	
		6. You can reduce your chance of developing a heart attack or stroke by eating at least five portions of fruit and vegetables a day.	
		7. Moderate intensity activity of 2 ½ hours a week will reduce your chances of developing a heart attack or stroke.	
		8. People who have diabetes are at higher risk of having a heart attack or stroke.	
		9. Managing your stress levels will help you to manage your blood pressure.	
		10. The healthiest exercise for the heart involves rapid breathing for 10 minutes or more.	
		11. Many vegetables are high in cholesterol.	
		12. You are more likely to have a heart attack or stroke if you're overweight or obese.	
		13. Drinking high levels of alcohol can increase your cholesterol and triglyceride levels.	
		14. HDL refers to 'good' cholesterol, and LDL refers to 'bad' cholesterol.	
		15. Family history of heart disease is not a risk	

For peer review only - http://bmjopen.bmj.com/site/about/guidelines.xhtml

		factor for high blood pressure.	
**Perceived CVD Risk** (15 items)<br><br>Composite score = sum across subscales. Higher score = higher perception of risk of having a heart attack or stroke	**Dread Risk** (7 items)	16. There is a possibility that I will have a heart attack or stroke.	Higher sum score = Higher perceived lack of control, dread, catastrophic potential and fatal consequences<br><br>1 = Strongly disagree; 2 = disagree; 3 = agree; 4 = strongly agree; N/A = 0
		17. There is a good chance I will experience a heart attack or stroke in the next 10 years.	
		18. It is likely I will have a heart attack or stroke because of my past and/or present behaviours.	
		19. I feel sure that I will have a heart attack or stroke.	
		20. It is likely that I will have a heart attack or stroke some time during my life.	
		21. I am at risk for having a heart attack or stroke some time during my life.	
		22. It is possible that I will have a heart attack or stroke within the next 10 years.	
	**Risk** (3 items)	23. I am too young to have a heart attack or stroke.	Higher sum score = Higher perceived hazards that has few, moderate, known outcomes and consequences<br><br>**Reverse coded**<br>4 = Strongly disagree; 3 = disagree; 2 = agree; 1 = strongly agree; N/A = 0
		24. People like me do not have a heart attack or stroke.	
		25. I am not worried that I might have a heart attack or stroke.	
	**Unknown Risk** (5 items)	26. I am not doing anything now that is unhealthy to my heart.	Higher sum score = Higher perceived hazards judged to be unobservable, unknown, new, and delayed in their manifestation of harm<br><br>**Reverse coded**<br>4 = Strongly disagree; 3 = disagree; 2 = agree; 1 = strongly agree; N/A = 0
		27. I am very healthy so I will not have a heart attack or stroke.	
		28. My lifestyle habits do not put me at risk for having a heart attack or stroke.	
		29. No matter what I do, if I am going to have a heart attack or stroke, I will have one.	
		30. People who do not have a heart attack or stroke are just plain lucky.	

For peer review only - http://bmjopen.bmj.com/site/about/guidelines.xhtml

**CVD Health Beliefs** (13 items)	**Susceptibility** (4 items)	31. It is likely that I will suffer from a heart attack or stroke in the future.	Higher average score = Higher perceived personal risk of heart attack and stroke<br><br>1 = Strongly disagree; 2 = disagree; 3 = agree; 4 = strongly agree; N/A = 0
		32. My chances of suffering from a heart attack or stroke in the next 10 years are great.	
		33. I feel I will suffer from a heart attack or stroke sometime during my life.	
		34. I am concerned about the likelihood of having a heart attack or stroke in the near future.	
	**Severity** (3 items)	35. Heart attacks and strokes are always fatal.	Higher average score = Higher perceived severity of heart attack and stroke<br><br>1 = Strongly disagree; 2 = disagree; 3 = agree; 4 = strongly agree; N/A = 0
		36. My whole life would change if I had a heart attack or stroke.	
		37. If I have a heart attack or stroke I will die within 10 years.	
	**Benefits** (4 items)	38. Eating at least five portions of fruit and vegetables a day will decrease my chances of having a heart attack or stroke.	Higher average score = Higher perceived benefits of diet, exercise, consuming less alcohol and smoking cessation for reducing risk for heart attack and stroke<br><br>**Reverse coded**<br>4 = Strongly disagree; 3 = disagree; 2 = agree; 1 = strongly agree; N/A = 0
		39. Increasing my exercise to at least 2 ½ hours a week will decrease my chances of having a heart attack or stroke.	
		40. When I exercise for at least 2 ½ hours a week I am doing something good for the health of my heart.	
		41. When I eat at least five portions of fruit and vegetables a day I am doing something good for the health of my heart.	
	**Barriers** (2 items)	42. I do not have time to exercise on most days of the week.	Higher average score = Higher perception of select barriers to engaging in heart attack and stroke risk reducing behaviours<br><br>1 = Strongly disagree; 2 = disagree; 3 = agree; 4 = strongly agree; N/A = 0
		43. I cannot afford to buy healthy foods.	
**Self**	**CVD risk**	How confident are you that you know how to or can…	Higher average score = higher perceived

For peer review only - http://bmjopen.bmj.com/site/about/guidelines.xhtml

**Efficacy**<br><br>(5 items)	**reduction**<br><br>**self efficacy**<br><br>(5 items)	44. Control the risks of having a heart attack or stroke.	confidence<br><br>1 = Not at all confident; 2 = somewhat confident; 3 = very confident; 4 = completely confident; N/A = 0
		45. Maintain a healthy weight by exercising at least 2½ hours a week within the next two months.	
		46. Stop smoking if you want to.	
		47. Drink within the recommended levels of alcohol.	
		48. Eat at least five portions of fruit and vegetables per day within the next two months.	
**Intention /**<br><br>**Readiness**<br><br>**to Change**<br><br>(17 items)	**Exercise**<br><br>(4 items)	49. I am not thinking about exercising for 2 ½ hours a week.	Higher average score = Higher perceived readiness for change with regard to exercise behaviour<br><br>1 = Strongly disagree; 2 = disagree; 3 = agree; 4 = strongly agree; N/A = 0
		50. I am thinking about exercising at least 2 ½ hours a week.	
		51. I intend or want to exercise at least 2 ½ hours a week.	
		52. I am ready or have started to exercise 2 ½ hours a week.	
	**Diet**<br><br>(4 items)	53. I am not thinking about eating at least five portions of fruit and vegetables a day.	Higher average score = Higher perceived readiness for change with regard to health dietary behaviour<br><br>1 = Strongly disagree; 2 = disagree; 3 = agree; 4 = strongly agree; N/A = 0
		54. I am thinking about eating at least five portions of fruit and vegetables a day.	
		55. I intend or want to eat at least five portions of fruit and vegetables a day.	
		56. I am ready or started to eat at least five portions of fruit and vegetables a day.	
	**Alcohol**<br><br>(4 items)	57. I am thinking about cutting down on alcohol.	Higher average score = Higher perceived readiness for change with regard to alcohol consumption behaviour<br><br>1 = Strongly disagree; 2 = disagree; 3 = agree; 4 = strongly agree; N/A = 0
		58. I intend or want to cut down on alcohol.	
		59. I have been cutting down on alcohol.	
		60. I am not thinking about cutting down on alcohol.	
	**Smoking**<br><br>(5 items)	61. I am thinking of stopping smoking within two months.	Higher average score = Higher perceived readiness for change with regard to smoking cessation

		62. I have reduced or stopped smoking.	behaviour<br><br>1 = Strongly disagree; 2 = disagree; 3 = agree; 4 = strongly agree; N/A = 0
		63. I intend or want to stop smoking.	
		64. If I stop smoking it will reduce my chances of having a heart attack or stroke.	
		65. I am not thinking about stopping smoking.	

For peer review only

For peer review only - http://bmjopen.bmj.com/site/about/guidelines.xhtml

Appendix C. Population characteristics of 110 NHS Health Check attendees

Population Characteristics		n	% Total
Gender	Male	51	46.4%
	Female	56	50.9%
Age group	40-49	45	40.9%
	50-59	40	36.4%
	60-74	14	12.7%
Ethnicity	White	93	84.5%
	Mixed	2	1.8%
	Asian	2	1.8%
	Black	4	3.6%
	Other	4	3.6%
*Deprivation**	IMD 1 - least deprived	14	12.7%
	IMD 2	30	27.3%
	IMD 3	12	10.9%
	IMD 4	31	28.2%
	IMD 5 - most deprived	14	12.7%
Cholesterol	Raised total cholesterol TC≥ 5 mmol/l	66	60.0%
Blood pressure	High blood pressure BP ≥ 140/90 mm Hg	28	25.5%
Body Mass Index (BMI)	Obese (BMI>=30)	26	23.6%
Physical activity	Physically inactive	22	20.0%
Smoking status	Smokers	20	18.2%
Alcohol consumption	Excessive drinkers	13	11.8%
*10 year predicted risk of CVD***	High CVD Risk (QRisk2>=20%)	5	4.5%
	Medium CVD Risk (10%=<Qrisk2<20%)	21	19.1%
	Low CVD Risk (QRisk2<10%)	85	77.3%
Mean Values & 95% Confidence Intervals	Age (95% CI)	51.52	(49.93, 53.12)
	Total Cholesterol (95% CI)	5.42	(5.19, 5.64)
	HDL Cholesterol (95% CI)	1.44	(1.36, 1.53)
	Cholesterol Ratio (TC/HDL) (95% CI)	4.12	(3.73, 4.52)
	SBP (95% CI)	129.60	(125.76, 133.44)
	DBP (95% CI)	81.63	(79.62, 83.63)
	BMI (95% CI)	27.24	(26.17, 28.31)
	Q-Risk 2 (95% CI)	6.27	(5.19, 7.34)

*Deprivation was measured using the Index of Multiple Deprivation (IMD).

**Ten year predicted risk of CVD was estimated using the Q-Risk 2 algorithm[1]

Notes: SBP = systolic blood pressure; DBP = diastolic blood pressure; CI = confidence interval; HDL = high density lipoprotein; CVD = cardiovascular disease

References

1. Hippisley-Cox J, Coupland C, Vinogradova Y, et al. Predicting cardiovascular risk in England and Wales: prospective derivation and validation of QRISK2. *BMJ*. 2008; 336.

For peer review only - http://bmjopen.bmj.com/site/about/guidelines.xhtml

Appendix D. The ABCD Risk Questionnaire and scoring guide

Scale	Items	Coding
**Knowledge**<br><br>Higher sum score = more knowledgeable / more correct about having a heart attack or stroke	1. One of the main causes of heart attack and stroke is stress.<br><br>2. Walking and gardening are considered types of exercise that can lower the risk of having a heart attack or stroke.<br><br>3. Moderately intense activity of 2 ½ hours a week will reduce your chances of having a heart attack or stroke.<br><br>4. People who have diabetes are at higher risk of having a heart attack or stroke.<br><br>5. Managing your stress levels will help you to manage your blood pressure.<br><br>6. Drinking high levels of alcohol can increase your cholesterol and triglyceride levels.<br><br>7. HDL refers to 'good' cholesterol, and LDL refers to 'bad' cholesterol.<br><br>8. A family history of heart disease is not a risk factor for high blood pressure.	Correct Answers:<br><br>Q1-T<br><br>Q2-T<br><br>Q3-T<br><br>Q4-T<br><br>Q5-T<br><br>Q6-T<br><br>Q7-T<br><br>Q8-F<br><br>T= True<br>F= False<br><br>Correct: Score = 1,<br>Incorrect or Don't Know: Score = 0.
**Perceived Risk of**<br><br>**Heart**<br><br>**Attack/Stroke**<br><br>Higher sum score = higher perception of risk of having a heart attack or stroke	9. I feel I will suffer from a heart attack or stroke sometime during my life.	1=Strongly disagree; 2 = disagree; 3 = agree; 4 = strongly agree; N/A = 0
	10. It is likely that I will suffer from a heart attack or stroke in the future.	1=Strongly disagree; 2 = disagree; 3 = agree; 4 = strongly agree; N/A = 0
	11. It is likely that I will have a heart attack or stroke some time during my life.	1=Strongly disagree; 2 = disagree; 3 = agree; 4 = strongly agree; N/A = 0
	12. There is a good chance I will experience a heart attack or stroke in the next 10 years.	1=Strongly disagree; 2 = disagree; 3 = agree; 4 = strongly agree; N/A = 0
	13. My chances of suffering from a heart attack or stroke in the next 10 years are great.	1=Strongly disagree; 2 = disagree; 3 = agree; 4 = strongly agree; N/A = 0
**Perceived Risk of**	14. It is likely I will have a heart attack or stroke because of my past and/or present behaviours.	1=Strongly disagree; 2 = disagree; 3 = agree; 4 = strongly agree; N/A = 0

For peer review only - http://bmjopen.bmj.com/site/about/guidelines.xhtml

Scale	Items	Coding
**Heart Attack/Stroke**	15. I am not worried that I might have a heart attack or stroke.	**Reverse coded** 4=Strongly disagree; 3 = disagree; 2 = agree; 1 = strongly agree; N/A = 0
	16. I am concerned about the likelihood of having a heart attack or stroke in the near future.	1=Strongly disagree; 2 = disagree; 3 = agree; 4 = strongly agree; N/A = 0
**Perceived Benefits and Intentions to Change** Higher average score = Higher perceived benefits of diet and exercise and higher perceived readiness for change in regards to exercise behaviour	17. I am thinking about exercising at least 2½ hours a week.	1=Strongly disagree; 2 = disagree; 3 = agree; 4 = strongly agree; N/A = 0
	18. I intend or want to exercise at least 2½ hours a week.	1=Strongly disagree; 2 = disagree; 3 = agree; 4 = strongly agree; N/A = 0
	19. When I exercise for at least 2½ hours a week I am doing something good for the health of my heart.	1=Strongly disagree; 2 = disagree; 3 = agree; 4 = strongly agree; N/A = 0
	20. I am confident that I can maintain a healthy weight by exercising at least 2½ hours a week within the next two months.	1=Strongly disagree; 2 = disagree; 3 = agree; 4 = strongly agree; N/A = 0
	21. I am not thinking about exercising for 2 ½ hours a week.	**Reverse coded** 4=Strongly disagree; 3 = disagree; 2 = agree; 1 = strongly agree; N/A = 0
	22. When I eat at least five portions of fruit and vegetables a day I am doing something good for the health of my heart.	1=Strongly disagree; 2 = disagree; 3 = agree; 4 = strongly agree; N/A = 0
	23. Increasing my exercise to at least 2½ hours a week will decrease my chances of having a heart attack or stroke.	1=Strongly disagree; 2 = disagree; 3 = agree; 4 = strongly agree; N/A = 0
**Healthy Eating Intentions** Higher average score = Higher perceived readiness for change with regard to health dietary behaviour	24. I am confident that I can eat at least five portions of fruit and vegetables per day within the next two months.	1=Strongly disagree; 2 = disagree; 3 = agree; 4 = strongly agree; N/A = 0
	25. I am thinking about eating at least five portions of fruit and vegetables a day.	1=Strongly disagree; 2 = disagree; 3 = agree; 4 = strongly agree; N/A = 0
	26. I am not thinking about eating at least five portions of fruit and vegetables a day.	**Reverse coded** 4=Strongly disagree; 3 = disagree; 2 = agree; 1 = strongly agree; N/A = 0

The Attitudes and Beliefs about Cardiovascular Disease (ABCD) Risk Questionnaire is licensed under the Creative Commons Attribution-NonCommercial-NoDerivatives 4.0 International License

For peer review only - http://bmjopen.bmj.com/site/about/guidelines.xhtml

Appendix E. Correlations of Factors of the ABCD Risk Questionnaire with Predicted CVD Risk using Spearman's Rho among 110 NHS Health Check Attendees

		KNOWLEDGE	PERCEIVED RISK	PERCEIVED BENEFITS	HEALTHY INTENTIONS	IMD2010_Q uintile	BMI	QRISK2	QRISK2_L_M_H
KNOWLEDGE	Correlation Coefficient	1.000	-.124	-.148	-.106	-.002	-.225*	-.007	-.063
	Sig. (2-tailed)	.	.236	.175	.319	.986	.021	.941	.522
	N	107	93	86	91	99	105	104	104
PERCEIVED RISK	Correlation Coefficient	-.124	1.000	-.195	-.188	.239*	.389**	.220*	.173
	Sig. (2-tailed)	.236	.	.080	.088	.025	.000	.036	.102
	N	93	95	82	84	87	92	91	91
PERCEIVED BENEFITS	Correlation Coefficient	-.148	-.195	1.000	.533**	-.287**	-.068	-.118	-.232*
	Sig. (2-tailed)	.175	.080	.	.000	.009	.538	.284	.033
	N	86	82	88	83	81	85	84	84
HEALTHY INTENTIONS	Correlation Coefficient	-.106	-.188	.533**	1.000	-.261*	.084	-.072	-.116
	Sig. (2-tailed)	.319	.088	.000	.	.016	.430	.504	.279
	N	91	84	83	93	85	90	89	89

12

		KNOWLEDGE	PERCEIVED RISK	PERCEIVED BENEFITS	HEALTHY INTENTIONS	IMD2010_Quintile	BMI	QRISK2	QRISK2_L_M_H
IMD2010_Quintile	Correlation Coefficient	-.002	.239*	-.287**	-.261*	1.000	-.008	.009	.017
	Sig. (2-tailed)	.986	.025	.009	.016	.	.938	.931	.870
	N	99	87	81	85	101	101	100	100
BMI	Correlation Coefficient	-.225*	.389**	-.068	.084	-.008	1.000	.020	.028
	Sig. (2-tailed)	.021	.000	.538	.430	.938	.	.839	.777
	N	105	92	85	90	101	107	106	106
QRISK2	Correlation Coefficient	-.007	.220*	-.118	-.072	.009	.020	1.000	.694**
	Sig. (2-tailed)	.941	.036	.284	.504	.931	.839	.	.000
	N	104	91	84	89	100	106	106	106
QRISK2_L_M_H	Correlation Coefficient	-.063	.173	-.232*	-.116	.017	.028	.694**	1.000
	Sig. (2-tailed)	.522	.102	.033	.279	.870	.777	.000	.
	N	104	91	84	89	100	106	106	106

* Correlation is significant at the 0.05 level (2-tailed). ** Correlation is significant at the 0.01 level (2-tailed).

QRisk2_L_M_H categorizes predicted CVD risk from Low CVD Risk (QRISK2<10%), to Medium CVD Risk (10%<=QRISK2<20%), to High CVD Risk (QRISK2>=20%).

IMD2010Quintile categorizes deprivation from 1=least deprived to 5=most deprived.
